# *SlGID1a* Is a Putative Candidate Gene for *qtph1.1*, a Major-Effect Quantitative Trait Locus Controlling Tomato Plant Height

**DOI:** 10.3389/fgene.2020.00881

**Published:** 2020-08-07

**Authors:** Xiaolin Liu, Wencai Yang, Jing Wang, Mengxia Yang, Kai Wei, Xiaoyan Liu, Zhengkun Qiu, Tong van Giang, Xiaoxuan Wang, Yanmei Guo, Junming Li, Lei Liu, Jinshuai Shu, Yongchen Du, Zejun Huang

**Affiliations:** ^1^Key Laboratory of Biology and Genetic Improvement of Horticultural Crops of the Ministry of Agriculture, Institute of Vegetables and Flowers, Chinese Academy of Agricultural Sciences, Beijing, China; ^2^Department of Vegetable Science, College of Horticulture, China Agricultural University, Beijing, China; ^3^Key Laboratory of Horticultural Crop Biology and Germplasm Innovation in South China, Ministry of Agriculture, College of Horticulture, South China Agricultural University, Guangzhou, China

**Keywords:** tomato (*Solanum lycopersicum*), plant height, quantitative trait locus, fine mapping, *SlGID1a*, transcriptome analysis

## Abstract

Plant height is an important agronomic trait in crops. Several genes underlying tomato (*Solanum lycopersicum*) plant height mutants have been cloned. However, few quantitative trait genes for plant height have been identified in tomato. In this study, seven quantitative trait loci (QTLs) controlling plant height were identified in tomato. Of which, *qtph1.1* (*QTL for tomato plant height 1.1*), *qtph3.1* and *qtph12.1* were major QTLs and explained 15, 16, and 12% of phenotypic variation (R^2^), respectively. The *qtph1.1* was further mapped to an 18.9-kb interval on chromosome 1. Based on the annotated tomato genome (version SL2.50, annotation ITAG2.40), *Solyc01g098390* encoding GA receptor SlGID1a was the putative candidate gene. The *SlGID1a* gene underlying the *qtph1.1* locus contained a single nucleotide polymorphism (SNP) that resulted in an amino acid alteration in protein sequence. The near-isogenic line containing the *qtph1.1* locus (NIL-*qtph1.1*) exhibited shorter internode length and cell length than the wild type (NIL-WT). The dwarf phenotype of NIL-*qtph1.1* could not be rescued by exogenous GA_3_ treatment. Transcriptome analysis and real-time quantitative reverse transcription PCR (qPCR) showed that several genes related to biosynthesis and signaling of GA and auxin were differentially expressed in stems between NIL-*qtph1.1* and NIL-WT. These findings might pave the road for understanding the molecular regulation mechanism of tomato plant height.

## Introduction

Plant height is an important agronomic trait in crops. It can affect crop architecture, crowding tolerance, water and fertilizer management, and mechanical harvesting, which in turn affect economic benefits and yield of crops. The success of the “green revolution” in the 1960s is mainly due to the introduction of high-yielding semi-dwarf varieties of wheat and rice combining with the application of agricultural mechanization, irrigation and agrochemical/fertilizer. Plant height is controlled by many genes, of which most are related to biosynthesis or signaling of plant hormones, such as auxin, brassinosteroids (BRs), gibberellins (GAs), and strigolactones (SLs) (Sakamoto and Matsuoka, 2004; Salas Fernandez et al., 2009; Liu et al., 2018).

Tomato (*Solanum lycopersicum*) is one of the most important vegetable crops worldwide. Processing tomatoes are cultivated in open fields and are adapted for farm machinery throughout nearly the whole process of production. However, fresh-market tomatoes are usually grown on stakes or with strings in open fields or in protected agricultural areas (greenhouse, shade-house, and tunnel), requiring intensive labor for harvesting, staking, and tying. The compact growth habit (CGH) tomato with a lower plant height is an ideal fresh-market tomato architecture for mechanical harvest and reduces manual dependence (Frasca et al., 2014; Lee et al., 2018).

Tomato plant height is mainly determined by the number and length of internodes. Several mutants related to plant height have been reported and some genes responsible for the phenotypes have been cloned in tomato. Mutants *self-pruning* (*sp*) (MacArthur, 1932), *semideterminate* (*sdt*) (Elkind et al., 1991), and *suppressor of sp* (*ssp*) (Park et al., 2014) affect the number of internodes, while mutants *brachytic* (*br*) (Lee et al., 2018), *dwarf* (*d*) (Bishop et al., 1996), *Elongated Internode* (*EI*) (Sun et al., 2019), *gibberellin deficient-1* (*gib-1*), *gib-2*, *gib-3* (Koornneef et al., 1990), *procera* (*pro*) (Jupe et al., 1988), *short internode* (*si*) (Kwon et al., 2020), and *tomato internode elongated -1* (*tie-1*) (Schrager-Lavelle et al., 2019) affect internode length. The *sp* mutant shows determinate growth habit. Its sympodial units are terminated by inflorescences with average one to two vegetative nodes between inflorescences. While, indeterminate plants (wild type, WT) can continuously produce inflorescences that are separated by three vegetative nodes (MacArthur, 1932; Pnueli et al., 1998). Semideterminate plants exhibit sympodial units that are also terminated by inflorescences. However, they produce more inflorescences on the main stem than determinate plants, and their inflorescences are usually separated by two vegetative nodes (Elkind et al., 1991). *SP* gene is the ortholog of *CENTRORADIALIS* (*CEN*) from *Antirrhinum* and *TERMINAL FLOWER 1* (*TFL1*) from *Arabidopsis* (Pnueli et al., 1998), which belongs to the *CETS* (*CENTRORADIALIS*/*TERMINAL FLOWER 1/SELF-PRUNING*) gene family (McGarry and Ayre, 2012). Several members of *CETS* gene family in tomato are also related to plant growth habits. For example, *Solanum pennellii* allele of *SP5G* or *SP9D* combined with *sp* results in semideterminate growth (Fridman et al., 2002; Carmel-Goren et al., 2003; Jones et al., 2007). However, CR-*sp5g sp* double mutant exhibits more compactness than *sp* mutant (Soyk et al., 2017). The phenotype of *sp* plants can also be restored by mutation in the genes that are not members of the *CETS* gene family. For example, double mutant *ssp-610 sp* or *ssp-2129 sp* exhibits usually two vegetative nodes between inflorescences. Mutants *ssp-610* and *ssp-2129* both contain mutations in *SP-interacting G-BOX* (*SPGB*) gene (Park et al., 2014). Regardless of the growth habits, internode length can also affect plant height. The *d* locus reduces internode length and makes plants shorter. The *D* gene encodes a P450 that is involved in brassinolide synthesis (Bishop et al., 1996, 1999; Marti et al., 2006). *EI* and *tie-1* exhibit elongated internode. Both of them result from the loss function of the *GA 2-beta-dioxygenase 7* (*SlGA2ox7*) gene, which converts bioactive GAs to inactive GAs (Schrager-Lavelle et al., 2019; Sun et al., 2019). The *pro* mutant displays higher plant height than the wild type (WT), which is similar to the phenotype of the WT treated with exogenous GA. The *PRO* gene encodes a SlDELLA protein that is a negative regulator in GA signaling (Bassel et al., 2008). A mild hypomorphic allele of the *SlDELLA* gene, the *pro-2* mutant, is intermediate in plant height between the WT and *pro* mutant. The *pro-2* mutant produces more fruit than the WT and *pro* mutant, but most fruits are smaller and seedless (Shinozaki et al., 2018). The *si* mutant displays shortened internodes and flower/fruit stems. It contains a mutation in a gene homolog to *Arabidopsis ERECTA* (*ER*) (Kwon et al., 2020). The *br* locus can reduce plant height and has been narrowed down to an interval of 763.1 kb on chromosome 1 (Lee et al., 2018), but the gene has not been cloned.

Several quantitative trait loci (QTLs) for plant height have also been identified. deVicente and Tanksley (1993) identified 9 QTLs controlling plant height by using a population developed from the cross between cultivated tomato Vendor TM2a and *Solanum pennellii* LA716. They are *ht1*, *ht3*, *ht5a*, *ht5b*, *ht6*, *ht7*, *ht9*, *ht10*, and *ht11*. Grandillo and Tanksley (1996) identified a major-effect QTL controlling plant height on chromosome 2. Paran et al. (1997) identified several loci controlling plant height on chromosomes 2, 3, 4, 6, and 7. Prudent et al. (2009) discovered several loci related to plant height on chromosomes 3, 4, 9, 11, and 12. Zhou et al. (2016) found three QTLs related to plant height: *h4t2a*, *h4t3a*, and *h4t7a*. However, the plant height QTL has not been fine mapped in tomato, which limits the understanding of molecular mechanisms of tomato plant height regulation.

In this study, seven QTLs controlling tomato plant height were identified. *qtph1.1* (*QTL for tomato plant height 1.1*) was a major-effect QTL. It was further narrowed down to an interval of 18.9-kb on chromosome 1, and GA receptor gene *SlGID1a* was identified as the putative candidate gene. The *SlGID1a* gene underlying the *qtph1.1* locus contained a single nucleotide polymorphism (SNP) that resulted in an amino acid alteration in the protein sequence, and the near-isogenic line containing the *qtph1.1* locus (NIL-*qtph1.1*) reduced the effect of exogenous GA_3_ on plant height. Transcriptome analysis and real-time quantitative reverse transcription PCR (qPCR) showed that several genes, which are related to biosynthesis and signaling of GA and auxin, were differentially expressed between NIL-*qtph1.1* and NIL-WT. These findings may facilitate understanding the genetic basis and the molecular regulation mechanism of tomato plant height.

## Materials and Methods

### Plant Materials

SG-7 is a fresh-market tomato inbred line developed by our group. Seeds of cherry tomato LA1218 [accession number, syn. TS-165 (Lin et al., 2014)] were obtained from the Tomato Genetics Resource Center (TGRC, Davis, CA, United States). Both SG-7 and TS-165 have an indeterminate growth habit. Two hundred ninety-nine F_2_ plants derived from a cross between SG-7 and TS-165 were grown in soil in a glass greenhouse under natural day-length conditions and managed routinely in Haidian District, Beijing, China, in the spring and summer of 2015. The recombinants, heterozygous plants and progeny test populations for fine-mapping of the *qtph1.1* locus were grown in soil in a plastic greenhouse under natural day-length conditions and managed routinely in Shunyi District, Beijing, China, from 2016 to 2018. Plant height, defined as the height of the fourth truss in this study, was measured according to the method described in a previous study (Zhou et al., 2016). The near-isogenic lines NIL-WT (its *SlGID1a* gene was homologous for SG-7 allele) and NIL-*qtph1.1* (its *SlGID1a* gene was homologous for TS-165 allele) for GA_3_ treatment experiment were grown in pots containing the mixed peat-vermiculite (1:1, v/v) substrate in a glass greenhouse under natural day-length conditions and standard water and fertilizer regimes in Haidian District, Beijing, China, during the winter of 2018. The pedigrees of the materials used in this study were displayed in Supplementary Figure S1.

### Molecular Marker Development

Tomato lines SG-7 and TS-165 were re-sequenced on an Illumina Hiseq 2500 PE150 platform (Illumina, San Diego, CA, United States) with 11 × genome coverage. The paired-end reads were aligned to the tomato reference genome (version SL2.50) using BWA version 0.7.17 (BWA-MEM algorithm) (Li, 2013), and sorted and indexed using SAMtools version 1.6 (Li et al., 2009). The variants were called with the Genome Analysis ToolKit version 4.0.4.0 (McKenna et al., 2010). The insertion and deletion (InDel) markers were designed using the Primer-BLAST tool available through the National Center for Biotechnology Information (NCBI^1^). The cleaved amplified polymorphic sequence (CAPS) and derived CAPS (dCAPS) markers were designed using dCAPS Finder 2.0 (Neff et al., 2002). General information regarding the DNA markers used in this study was given in Supplementary Table S1.

### QTL Mapping

The QTL-seq approach was applied to identify loci controlling tomato plant height (Takagi et al., 2013). From 299 individuals in the F_2_ population, two pools comprising 25 plants/pool were generated. Pools PHH and PHS consisted of pooled DNA from plants featuring the tallest and shortest plant height, respectively. The two pools were re-sequenced on an Illumina Hiseq 2500 PE150 platform (Illumina, San Diego, CA, United States) with 11 × genome coverage. Using the SNPs of line SG-7 as a reference, an SNP-index was calculated for each SNP for each pool. Sliding window analysis was applied to calculate the average SNP-index across the genome with a 1 Mb window size and 10 kb step increment (Illa-Berenguer et al., 2015). Δ(SNP-index) was calculated by the SNP-index (PHH) subtracted by the SNP-index (PHS). The threshold line for the | Δ(SNP-index)| plot was set at 0.3 (Takagi et al., 2013) to identify candidate QTLs for tomato plant height.

InDel markers in the region of the candidate QTLs were used to genotype the whole F_2_ population. One-way analysis of variance (ANOVA) was used to test the significant association between markers and plant height. The degree of dominance or gene action was calculated as the d/a ratio, where d = Aa - (AA + aa)/2 and a = (AA - aa)/2, where AA was the mean value for the homozygous SG-7 allele, aa was the mean value for the homozygous TS-165 allele, and Aa was the mean heterozygous value. The percentage of phenotypic variation explained by each QTL (R^2^) was estimated using multiple-regression analysis, using as explanatory variables the most significant markers for each QTL (Illa-Berenguer et al., 2015).

### Recombinant Plant Selection and Progeny Test

Five recombinants (15N63-23, 15N63-49, 15N63-197, 15N63-277, 15N63-339), whose crossover sites were around the marker HP3809 on chromosome 1 and the intervals for *qtph3.1* and *qtph12.1* were homozygous, were selected from the F_2_ population to perform progeny tests in the spring of 2016. For the progeny test of each recombinant, 94 offspring seedlings were genotyped usually using two markers in the heterozygous region around the *qtph1.1* locus of their parent. A set of homozygous plants carrying the SG-7 allele (score 1) or the TS-165 allele (score 3) were selected to grow in the greenhouse and evaluate the plant height. At the same time, several recombinants and heterozygous plants (if no recombinants were found) detected from these offspring seedlings were selected to grow in the greenhouse and selfed for next generation progeny test. This strategy was used from F_3_ generation to F_7_ generation. Furthermore, lots of offspring seedlings were only used to get more recombinants. For fine-mapping of the *qtph1.1* locus, a total of 4,192 seedlings were genotyped from F_3_ generation to F_7_ generation. The pedigrees of the recombinants, heterozygous plants, and progeny test populations used in this study were exhibited in Supplementary Figure S1.

### Sequence Polymorphism Analysis

The genomic DNA fragments of the *qtph1.1* locus in SG-7 and TS-165 were obtained by overlapping PCR amplification using 2 × Taq PCR mix (Cat. No. M7122, Promega, Fitchburg, WI, United States) and sequencing the PCR products using specific primers (Supplementary Table S1). The amplified fragments were sequenced at the Beijing Genomics Institute (Beijing, China). The *SlGID1a* cDNAs of SG-7 and TS-165 were obtained by reverse-transcription PCR (RT-PCR) using Phusion High-Fidelity DNA polymerase (Cat. No. M0530L, New England Biolabs, Ipswich, MA, United States) with specific primers (Supplementary Table S1). The amplified fragments were cloned using the pEASY-Blunt Zero Cloning Kit (Cat. No. CB501-2, TransGen Biotech, Beijing, China). The cDNA clones were sequenced at the Beijing Genomics Institute (Beijing, China). Nucleotide sequence polymorphisms were identified by using BLASTn in the NCBI and multiple protein sequences were aligned by using Clustal X version 2.0 (Larkin et al., 2007) with default settings.

### Exogenous GA_3_ Treatment

The seeds of NIL-WT and NIL-*qtph1.1* were germinated on the filter paper moistened with deionized water in culture plates and were sown in plastic pots (one seed per pot) containing the mixed peat-vermiculite (1:1, v/v) substrate in a glasshouse under natural day-length conditions and standard water and fertilizer regimes in Haidian District, Beijing, China, in the winter of 2018. One hundred twenty pots were placed in the greenhouse evenly and made sure that they would not significantly impede each other’s growth. The positions of all the pots were changed every other day to reduce the influence of environmental factors on plant growth. After 4 weeks, the tomato plants were measured to determine the height and were then treated by spraying to runoff with 50 μM GA_3_ (Cat. No. G7645; Sigma, St Louis, MO, United States). The GA_3_ treatment was performed once every 2 days for a total of 10 times. The GA_3_ stock solution consisted of 50 mM GA_3_ and 70% ethanol used as the solvent. One milliliter GA_3_ stock solution was added into water to make the GA_3_ working solution with a final concentration of 50 μM GA_3_. For the control solution, 1 mL 70% ethanol was added to 999 mL water to achieve the equivalent amount of ethanol with working solution (Tomlinson et al., 2018). The tomato plants were divided into four groups: NIL-WT-GA_3_, NIL-*qtph1.1*-GA_3_, NIL-WT-Control, and NIL-*qtph1.1*-Control. Each group had 30 plants. During the GA_3_ treatment, we changed the positions of all plants in each group every 2 days, and changed the positions of four different groups every 4 days. After the third GA_3_ treatment, the sixth internodes of 15 plants per group were collected for RNA extraction. Two days after the last GA_3_ treatment on the remaining plants, the plant architecture parameters were measured and all of the plants were photographed using a camera (Canon EOS 70D, Canon Inc., Japan).

### Measurement of Plant Architecture Parameters

To compare the differences in plant morphology among the four groups, plant height (here it means the distance from the base of the plant to the top of the main stem) and internode length were measured. The plant height was measured during the period of GA_3_ treatment every 2 days, and the length of internodes was measured when the GA_3_ treatment was finished. For numbering of internodes, from the cotyledon to the first true leaf was designated as the first internode. A total of 12 internodes length were measured in this study. For all indexes of the tomato plants, at least 14 plants of each group were recorded.

### Histological Analysis of Stem Cells

The longitudinal sections of the eighth internodes were obtained using a free-hand method. The internodes were cut by a razor blade into approximately 1-mm-thick sections. The sections were put on glass slides and stained with 0.1% toluidine blue (w/v, dissolved in 1 × PBS solution, pH: 7.2-7.4; Cat. No. 89640; Sigma, St Louis, MO, United States). The stained sections were observed under a stereomicroscope (Carl Zeiss MicroImaging GmbH, Göttingen, Germany) and photographed. For each group, five sections from five different seedings were selected for further measuring the cell length. At least two hundred and forty-two cell’s lengths were measured from each section using Image version 1.52a (Collins, 2018).

### RNA Extraction

The sixth internodes were collected from plants of four groups after the third GA_3_ treatment. Each group comprised three biological replications, and each replication contained samples from five plants. A total of 12 samples were immediately frozen in liquid nitrogen and then stored at −80°C until RNA extraction. Total RNA was isolated using the Quick RNA Isolation Kit (Cat. No. BC1803, Huayueyang Biotech Co., Ltd., Beijing, China) according to the manufacturer’s instructions.

### RNA-Seq and Analysis of the Differentially Expressed Genes (DEGs)

Twelve libraries were constructed using the TruSeq RNA Library Prep Kit (Illumina Inc.) and sequenced on an Illumina platform by Beijing Nuohe Zhiyuan Company. DEGs were analyzed using edgeR (version 3.8.6) with the exact test method described by Lamarre et al. (2018). The versions of tomato reference genome and annotation database were SL2.50 and ITAG release 2.40 respectively. The criterion for DEGs was a false discovery rate (FDR) <0.05. The RNA-seq data have been deposited in the Genome Sequence Archive in BIG Data Center (Beijing Institute of Genomics, Chinese Academy of Sciences) under the accession number PRJCA002406.

### cDNA Synthesis and qPCR Analysis

cDNA was synthesized from 2 μg total RNA using GoScript^TM^ Reverse Transcriptase (Cat. No. A5003; Promega, Madison, WI, United States). The qPCR reactions were conducted using the GoScript^TM^ qPCR Master Mix (Cat. No. A6002; Promega, Madison, WI, United States) and the LightCycler 480 Detection System (Roche Diagnostics GmbH, Mannheim, Germany). The primers for qPCR were provided in Supplementary Table S1. qPCR and data analysis were performed using methods previously described (Cao et al., 2017). The tomato housekeeping gene *SlCAC* (*Solyc08g006960*) was used as an internal control (Exposito-Rodriguez et al., 2008). All qPCR analyses were conducted with three biological replications and three technical replications. The 2^–ΔCT^ method was used to calculate relative gene expression (Livak and Schmittgen, 2001) and the differences between the four groups were tested using the Tukey’s honestly significant difference test (*P* < 0.05).

## Results

### Plant Height Variation in the Segregating Population

**FIGURE 1:**
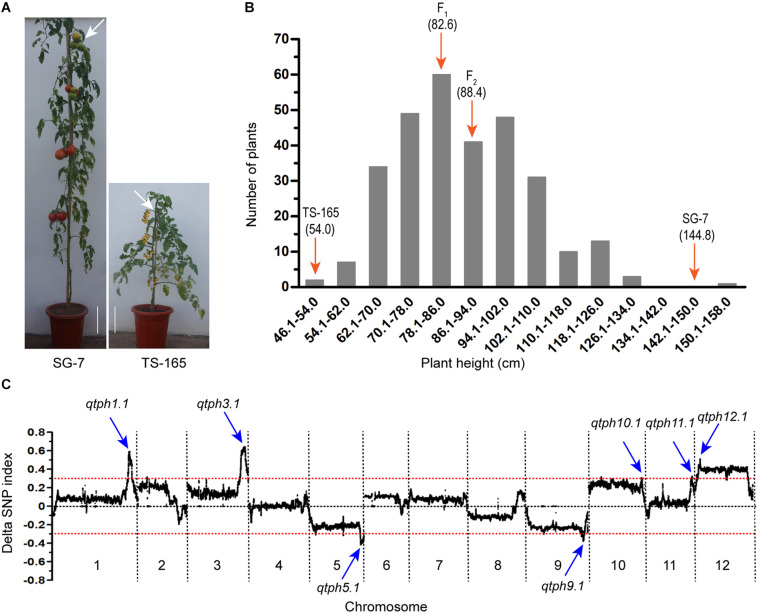
Identification of tomato plant height QTLs. **(A)** Plant height phenotype of tomato lines SG-7 and TS-165. Scale bars indicate 10 cm and white arrows point to the fourth truss. **(B)** Plant height distribution in the F_2_ population derived from the cross between SG-7 and TS-165. **(C)** Average values of the ΔSNP-index calculated by a sliding window from QTL-seq analysis.

The tomato line SG-7 was tall and TS-165 was short (Figure 1A). The plant heights of SG-7, TS-165 and F_1_ plants were 144.8, 54.0, and 82.6 cm, respectively. The internode length of SG-7, TS-165 and F_1_ plants were 7.2, 3.1, and 4.9 cm, respectively. In the F_2_ population, the correlation coefficient between plant height and average internode length was 0.96, suggesting that the difference of plant height in the two tomato lines was mostly determined by internode length. The frequency distribution of plant height in the F_2_ population showed continuous variation with the range of 48.0–158.0 cm (Figure 1B), suggesting that plant height in the two tomato lines was quantitatively inherited.

### QTL Analysis of Tomato Plant Height

Based on the plant height data, two extreme pools from the F_2_ population were prepared and subjected to QTL-seq. Pool PHH consisted of 25 tallest plants with plant height at the range of 111.0–158.0 cm, while pool PHS consisted of 25 shortest plants with plant height at the range of 48.0–66.0 cm. The two pools and two parental lines were re-sequenced, and a total of 1,285,779 SNPs were identified between the two parental lines. A graph of Δ(SNP-index) was generated by subtracting the SNP-index value of the pool PHS from the pool PHH (Figure 1C). Seven candidate intervals controlling tomato plant height were identified. They were located on chromosomes 1, 3, 5, 9, 10, 11, and 12 and were accordingly named *QTL for tomato plant height 1.1* (*qtph1.1*), *qtph3.1*, *qtph5.1*, *qtph9.1*, *qtph10.1*, *qtph11.1*, and *qtph12.1* (Figure 1C).

To confirm the QTLs for tomato plant height detected by QTL-seq, markers (Supplementary Table S1) within the seven candidate intervals controlling tomato plant height were used to genotype 299 F_2_ plants. One-way analysis of variance showed that the markers within the seven intervals were significantly associated with plant height (Supplementary Table S2). Among the seven QTLs, *qtph5.1* and *qtph9.1* contained the alleles for shorter plant height from SG-7, while the others from TS-165. *qtph1.1*, *qtph3.1*, and *qtph12.1* were major-effect QTLs (*R*^2^ ≥ 0.1). Phenotypic variation (*R*^2^) explained by these three QTLs were 15, 16, and 12%, respectively (Supplementary Table S2).

### Fine-Mapping of the *qtph1.1* Locus

**FIGURE 2:**
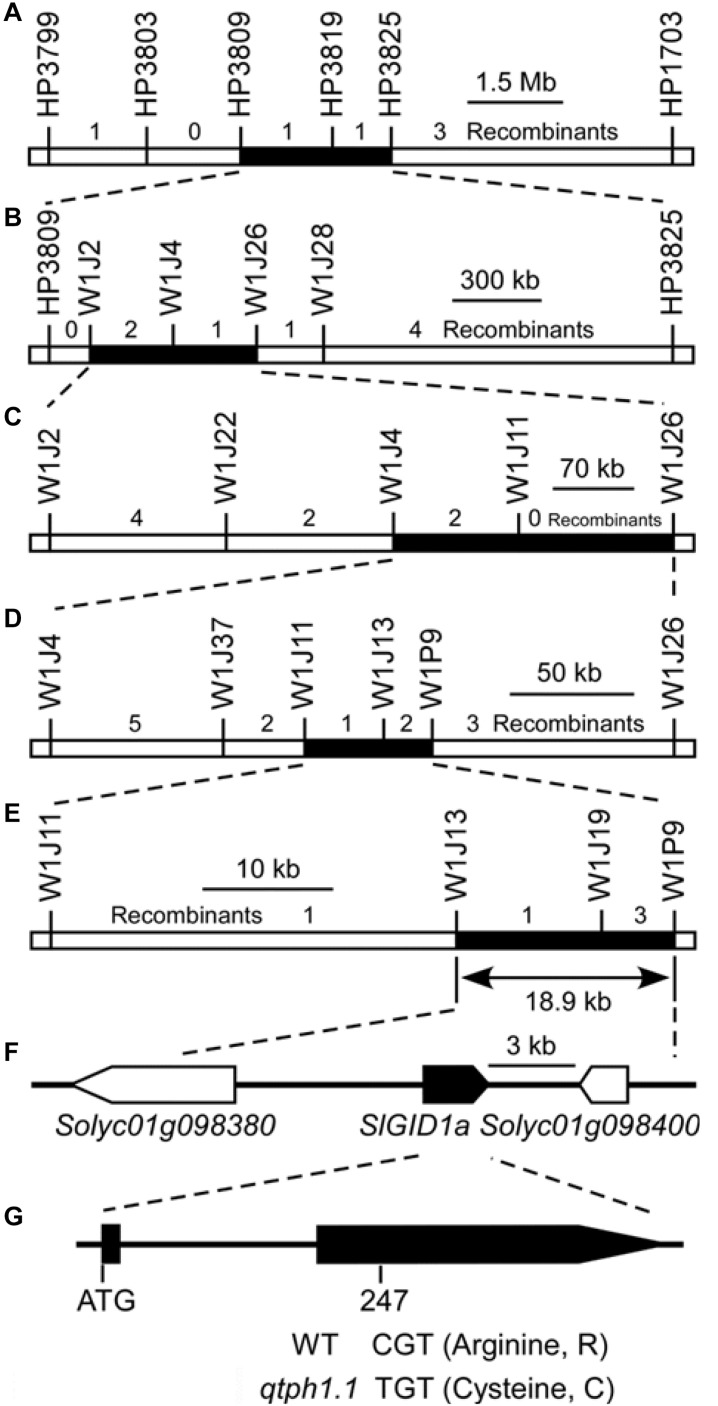
Fine mapping of the *qtph1.1* locus. **(A–E)** The results of five cycles of recombinant progeny tests. **(F)** The annotated genes of ITAG release 2.40. Arrows indicate the direction of transcription, and the solid arrow presents the putative candidate gene for *qTPH1.1*. **(G)** The exon-intron structure of the coding region of *SlGID1a* and the sequence polymorphism between *qtph1.1* (from TS-165) and the wild type (WT, from SG-7). The 247th nucleotide of the coding sequence of *SlGID1a* was C in SG-7 and T in TS-165, which led to the 83rd amino acid residue in the predicted protein sequence being Arginine (R) in SG-7 and Cysteine (C) in TS-165.

Five recombinants, whose crossover sites were around the marker HP3809 on chromosome 1 and the intervals for the loci *qtph3.1* and *qtph12.1* were homozygous, were selected from the F_2_ population to perform progeny tests in the spring of 2016. The progeny test showed that the *qtph1.1* locus was located between markers HP3809 and HP3825, a 2.3 Mb region on chromosome 1 (Figure 2A, Supplementary Figure S1 and Supplementary Table S3). In the autumn of 2016, spring and autumn of 2017, the *qtph1.1* locus was narrowed down to the regions between markers W1J2 and W1J26 (Figure 2B; Supplementary Figure S1 and Supplementary Table S3), between markers W1J4 and W1J26 (Figure 2C; Supplementary Figure S1 and Supplementary Table S3), and between markers W1J11 and W1P9 (Figure 2D; Supplementary Figure S1 and Table S3), respectively. Finally, the *qtph1.1* locus was fine-mapped to the 18.9-kb interval between markers W1J13 and W1P9 in the spring of 2018 (Figure 2E; Supplementary Figure S1 and Supplementary Table S3).

### Candidate Gene Analysis of *qTPH1.1*

Three putative genes were in the 18.9-kb region corresponding to the *qtph1.1* locus by searching the tomato genome annotation database (ITAG release 2.40) in SGN^2^ (Figure 2F and Supplementary Table S4). Of which, *Solyc01g098390* encoded Gibberellin receptor GIBBERELLIN INSENSITIVE DWARF1 A (SlGID1a). The 247th nucleotide of the coding sequence of *SlGID1a* was C in tomato line SG-7 and T in TS-165 (Figure 2G and Supplementary Figure S2), which led to the 83rd amino acid residue in the predicted protein sequence being Arginine (R) in SG-7 and Cysteine (C) in TS-165 (Figure 2G and Supplementary Figure S3). The allele of SlGID1a in TS-165 was named SlGID1a^R83C^ (Supplementary Figure S3). It has been known that the *GID1* gene plays an important role in gibberellin signaling in plants (Hirano et al., 2008). Loss-of-function mutation of the *SlGID1a* gene resulted in typical GA-insensitive dwarfism (Illouz-Eliaz et al., 2019). The amino acid residue R was fixed in all 169 GID1s (Yoshida et al., 2018), indicated that it was important structurally and functionally. Therefore, *SlGID1a* was the putative candidate gene for *qTPH1.1*.

### Response to GA_3_ Treatment

**FIGURE 3:**
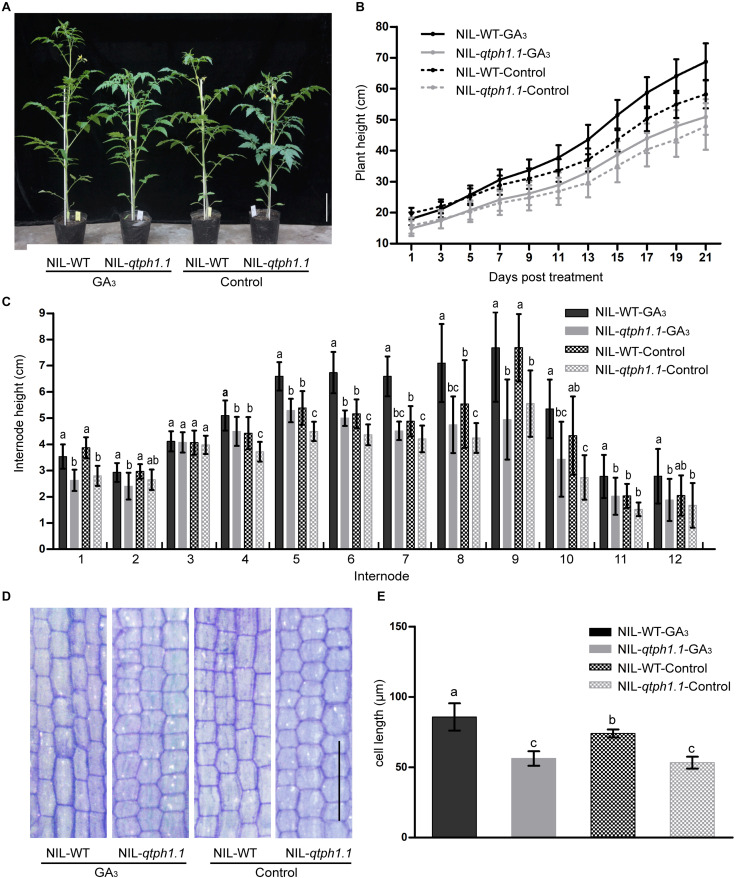
Response of the NIL-*qtph1.1* plants to GA_3_ treatment. **(A)** Plant height phenotype of NIL-WT and NIL-*qtph1.1* plants after GA_3_ treatment. Scale bar indicates 10 cm. **(B)** Plant height of NIL-WT and NIL-*qtph1.1* plants during GA_3_ treatment (*n* > 14). **(C)** Internode length of NIL-WT and NIL-*qtph1.1* plants after GA_3_ treatment (*n* > 14). **(D)** Histological analysis of the longitudinal section of the 8th internode of NIL-WT and NIL-*qtph1.1* plants after GA_3_ treatment. Scale bar indicates 200 μm. **(E)** Cell length of the longitudinal section of the 8th internode of NIL-WT and NIL-*qtph1.1* plants after GA_3_ treatment. Each group consists of five replications and each replication includes at least two hundred and forty-two cells. Values represent the mean ± standard deviation (SD). Different letters above the columns indicate statistically significant differences among groups (Tukey’s honestly significant difference test, *P* < 0.05).

Given that the GA receptor *SlGID1a* was the putative candidate gene for *qTPH1.1*, the sensitivity of the *qtph1.1* locus to exogenous GA_3_ was investigated. Near isogenic lines NIL-WT and NIL-*qtph1.1* plants were sprayed with 50 μM GA_3_ or ethanol solution as the control. Thereby, these plants were divided into four groups: NIL-WT-GA_3_, NIL-*qtph1.1*-GA_3_, NIL-WT-Control, and NIL-*qtph1.1*-Control. The NIL-WT-Control plants were significantly taller than the NIL-*qtph1.1*-Control plants, and the NIL-WT-GA_3_ plants were also significantly taller than the NIL-*qtph1.1*-GA_3_ plants during the GA_3_ treatment experiment. The NIL-*qtph1.1*-GA_3_ plants were a slightly taller than the NIL-*qtph1.1*-Control plants, but the difference was not statistically significant during the whole treatment period (Figures 3A,B). However, the NIL-WT-GA_3_ plants were significantly taller than the NIL-WT-Control plants since the third treatment (Figures 3A,B). These results suggested that the NIL-*qtph1.1* plants were insensitive to exogenous GA_3_ stimulation.

### Analysis of Internode Length and Cell Length

The length of each internode of the above mentioned plants was measured after treatment. Except for the 3rd and 12th internodes, the length of internodes of the NIL-*qtph1.1* plants was significantly shorter than that of the NIL-WT plants (Figure 3C), irrespective of the control or treatment with GA_3_. Histological analysis of the longitude section of the 8th internode also showed that the cell length of the NIL-*qtph1.1* plants was significantly shorter than that of the NIL-WT plants (Figures 3D,E) in both control and under GA_3_ treatment. Application of GA_3_ to the NIL-WT plants significantly increased internode cell length. However, the cellular effect of GA_3_ on the NIL-*qtph1.1* plants was not significant (Figures 3D,E). These results suggested that short cell length was the important reason underlying the reduced internode length, which resulted in lower plant height of the NIL-*qtph1.1* plants.

### Comparative RNA-Seq Analysis of the *qtph1.1* NILs

To dissect the molecular mechanisms underlying these phenotypic differences between the NIL-WT and NIL-*qtph1.1* plants, especially the expression pattern of GA or other hormone related genes, the total transcriptome of young stems of these two lines treated with GA_3_ or ethanol (control) was analyzed using RNA-seq. Each line and each treatment comprised three biological replications, and a total of 12 cDNA libraries were constructed. Approximately 6.0 Gb clean data were generated for each replication. A total of 1,393 significant DEGs were discovered, comprised 753 up-regulated and 640 down-regulated DEGs in the NIL-*qtph1.1*-GA_3_ plants compared to the NIL-WT-GA_3_ plants. A total of 97 significant DEGs were discovered, comprised 52 up-regulated and 45 down-regulated DEGs in the NIL-*qtph1.1*-Control plants compared to the NIL-WT-Control plants. There were 63 overlapping DEGs between NIL-*qtph1.1* and NIL-WT after two treatments, of which 42 were up-regulated and 21 were down-regulated in NIL-*qtph1.1*. The detailed information of all significant DEGs was listed in Supplementary Tables S5, S6. Among the 1,393 significant DEGs between the NIL-*qtph1.1*-GA_3_ plants and the NIL-WT-GA_3_ plants, 12 genes were related to GA biosynthesis and signaling, and 23 genes were related to auxin biosynthesis, transport, and signaling (Table 1).

**TABLE 1:** Differentially expressed genes related to GA and auxin biosynthesis and signaling in NIL-WT and NIL-*qtph1.1* after GA_3_ treatment.

Gene ID^a^	Log_2_FC^b^	FDR	Description
**Gibberellin**			
Solyc11g072310.1	5.85	1.15E-16	Gibberellin 20-oxidase-3, SlGA20ox3
Solyc03g119910.2	1.87	2.26E-13	Gibberellin 3-beta-hydroxylase, SlGA3ox2
Solyc09g074270.2	1.32	6.36E-04	Acetyl esterase, SlGID1b1
Solyc06g008870.2	1.12	6.91E-04	GID1-like gibberellin receptor, SlGID1b2
Solyc01g080900.2	0.74	8.41E-07	Cytochrome P450, SlKAO
Solyc03g113910.2	0.46	1.34E-05	Gibberellin-regulated protein 2
Solyc04g078390.1	0.39	1.89E-05	SlGID2/SlSLY1, F-box protein
Solyc11g011260.1	−0.15	4.37E-02	SlDELLA/PRO, GAI-like protein 1
Solyc03g120970.2	−0.33	6.19E-03	Gibberellin 2-beta-dioxygenase 2
Solyc02g089350.2	−0.37	4.54E-03	Gibberellin regulated protein, SlGAST1
Solyc03g116060.2	−0.57	3.55E-07	Gibberellin-regulated protein
Solyc11g011210.1	−0.60	9.27E-04	Gibberellin regulated protein
**Auxin**			
Solyc07g066560.1	1.35	1.48E-02	Auxin responsive SAUR protein, Small auxin up-regulated RNA65
Solyc01g091030.2	1.22	5.53E-04	Auxin-responsive family protein, Small auxin up-regulated RNA1
Solyc07g063850.2	0.92	5.39E-03	Indole-3-acetic acid-amido synthetase GH3.8
Solyc10g008520.2	0.72	2.55E-03	Auxin-responsive GH3-like
Solyc02g077880.2	0.41	6.36E-04	Auxin-repressed protein
Solyc07g016180.2	0.38	1.67E-04	Auxin response factor 19, Auxin Response Factor 7A
Solyc01g099840.2	0.28	2.68E-03	Auxin-repressed protein
Solyc09g007810.2	−0.24	1.25E-02	Auxin response factor 3, Auxin Response Factor 16A
Solyc06g053840.2	−0.27	1.01E-02	Auxin responsive protein, auxin-regulated IAA1
Solyc05g047460.2	−0.29	3.29E-03	Auxin response factor 19, Auxin Response Factor 7B
Solyc01g110660.2	−0.40	3.96E-02	Auxin-induced SAUR-like protein, Small auxin up-regulated RNA9
Solyc06g008590.2	−0.41	3.40E-03	Auxin responsive protein, auxin-regulated IAA10
Solyc06g053830.2	−0.47	9.59E-05	Auxin responsive protein, auxin-regulated IAA7
Solyc09g083280.2	−0.51	1.46E-08	Auxin responsive protein, auxin-regulated IAA23
Solyc01g110680.2	−0.60	8.36E-04	Auxin-induced SAUR-like protein
Solyc01g110630.2	−0.69	1.24E-02	Auxin-induced SAUR-like protein
Solyc04g007690.2	−0.71	1.01E-06	SlPIN3, Auxin efflux carrier
Solyc11g011710.1	−0.79	1.35E-02	Auxin-responsive protein, Small auxin up-regulated RNA95
Solyc01g110790.2	−0.79	5.10E-04	Auxin-induced SAUR-like protein
Solyc06g008580.2	−1.00	7.46E-07	Auxin responsive protein
Solyc08g021820.2	−1.15	1.11E-02	Auxin responsive protein, auxin-regulated IAA21
Solyc01g110730.2	−1.29	1.57E-02	Auxin-induced SAUR-like protein, Small auxin up-regulated RNA10
Solyc01g110770.2	−1.40	1.06E-03	Auxin-induced SAUR-like protein

*^*a*^According to the annotated tomato genome (version SL2.50, annotation ITAG2.40). ^*b*^log_2_FC means log_2_FC(NIL-*qtph1.1*-GA_3_/NIL-WT-GA_3_).*

### qPCR Verification of the DEGs Related to GA and Auxin Biosynthesis and Signaling

**FIGURE 4:**
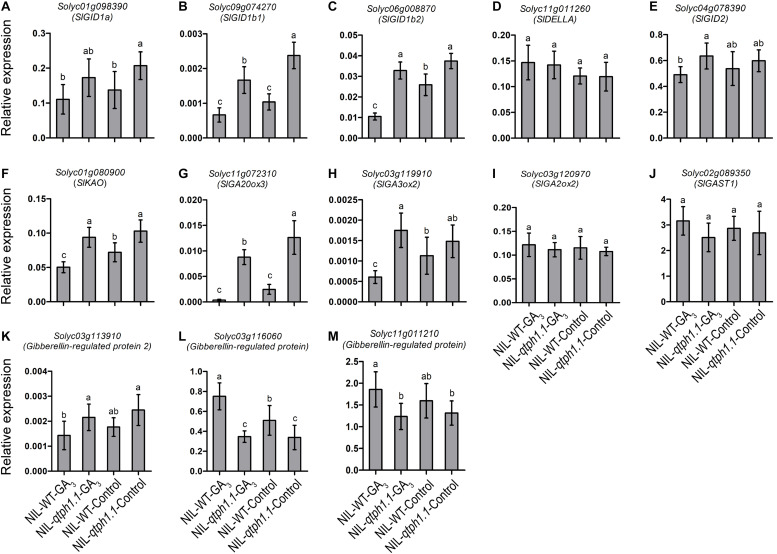
Relative expression of the genes related to GA biosynthesis and signaling in NIL-WT and NIL-*qtph1.1*. **(A–C)** The expression levels of three GA receptor genes, *Solyc01g098390*, *Solyc09g074270* and *Solyc06g008870*. **(D,E)** The expression levels of two GA signaling genes, *Solyc11g011260* and *Solyc04g078390*. **(F–I)** The expression levels of four GA biosynthesis genes, *Solyc01g080900*, *Solyc11g072310*, *Solyc03g119910* and *Solyc03g120970*. **(J–M)** The expression levels of four GA-regulated protein genes, *Solyc02g089350*, *Solyc03g113910*, *Solyc03g116060* and *Solyc11g011210* in NIL-WT and NIL-*qtph1.1* after treatment with GA_3_ and control solution. The *SlCAC* (*Solyc08g006960*) gene was used as a reference. Values represent the mean ± standard deviation (SD) based on the data of 3 biological replications and 3 technical replications. Different letters above the columns indicate statistically significant differences among groups (Tukey’s honestly significant difference test, *P* < 0.05).

To validate the DEGs identified by RNA-seq, the transcription expression of several genes related to GA and auxin biosynthesis and signaling was verified by qPCR (Table 1). *SlGID1a*, the putative candidate gene of *qTPH1.1*, was highly expressed in the NIL-*qtph1.1* plants compared to the NIL-WT plants (Figure 4A). The expression of the two other GA receptor genes, *SlGID1b1* (*Solyc09g074270*) and *SlGID1b2* (*Solyc06g008870*), was also up-regulated in the NIL-*qtph1.1* plants (Figures 4B,C). The suppressor gene in GA signaling, *SlDELLA* (*PRO*, *Solyc11g011260*), did not show significantly different expression between the two lines (Figure 4D). The F-box gene *SlGID2* (*SlSLY1*, *Solyc04g078390*) was only more highly expressed in the NIL-*qtph1.1* plants when treated with GA_3_ (Figure 4E). Three GA biosynthesis genes, *SlKAO* (*Solyc01g080900*), *SlGA20ox3* (*Solyc11g072310*), and *SlGA3ox2* (*Solyc03g119910*), were up-regulated in the NIL-*qtph1.1* plants (Figures 4F–H), whereas *SlGA2ox2* (*Solyc03g120970*) was almost expressed equally between the two lines (Figure 4I). Among the four GA-regulated protein genes, *SlGAST1* (*Solyc02g089350*) was almost expressed equally between lines (Figure 4J), *Solyc03g113910* was up-regulated in the NIL-*qtph1.1* plants (Figure 4K), but *Solyc03g116060* and *Solyc11g011210* were down-regulated in the NIL-*qtph1.1* plants (Figures 4L,M).

**FIGURE 5:**
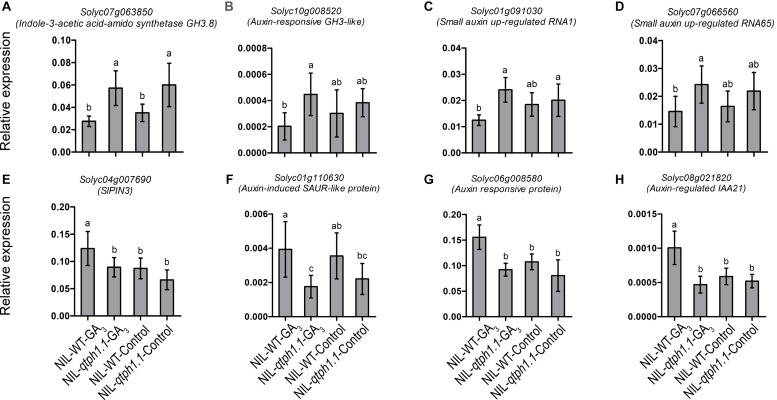
Relative expression of the genes related to auxin homeostasis, transporting and signaling in NIL-WT and NIL-*qtph1.1*. **(A,B)** The expression levels of two genes which prevent free IAA accumulation, *Solyc07g063850* and *Solyc10g008520*. **(C,D)** The expression levels of two small auxin up-regulated genes, *Solyc01g091030* and *Solyc07g066560*. **(E,F)** The expression levels of auxin efflux carrier *Solyc04g007690* and auxin-induced SAUR-like protein gene *Solyc01g110630*. **(G,H)** The expression levels of two auxin responsive protein (Aux/IAA like) genes, *Solyc06g008580* and *Solyc08g021820* in NIL-WT and NIL-*qtph1.1* after treatment with GA_3_ and control solution. The *SlCAC* (*Solyc08g006960*) gene was used as a reference. Values represent the mean ± standard deviation (SD) based on the data of 3 biological replications and 3 technical replications. Different letters above the columns indicate statistically significant differences among groups (Tukey’s honestly significant difference test, *P* < 0.05).

Four genes related to auxin biosynthesis and signaling were verified to be up-regulated in the NIL-*qtph1.1* plants, which included two genes that might prevent free IAA accumulation, *Solyc07g063850* (*GH3.8*) and *Solyc10g008520* (*GH3-like*) (Figures 5A,B), and two small auxin up-regulated (SAUR) genes, *Solyc01g091030* (*SAUR1*) and *Solyc07g066560* (*SAUR65*) (Figures 5C,D). Four genes related to auxin transportation and signaling were verified to be down-regulated in the NIL-*qtph1.1* plants, which included auxin efflux carrier *SlPIN3* (*Solyc04g007690*) (Figure 5E), auxin-induced SAUR-like protein gene *Solyc01g110630* (Figure 5F), and two auxin responsive protein (Aux/IAA like) genes, *Solyc06g008580* and *Solyc08g021820* (Figures 5G,H).

## Discussion

Tomato plant height is an important trait related to plant architecture. Several tomato plant height mutants have been identified, and their genetic basis has been discovered. However, only *br*, *d* and *sp* have been used in breeding because they have no or less negative effects on fruit size and yield (Scott and Harbaugh, 1989; Lukyanenko, 1990; Panthee and Gardner, 2013; Frasca et al., 2014). The variation of plant height in natural tomato lines is relatively large. QTL mapping is helpful to the analysis of the genetic basis of tomato plant height and the improvement of this trait. Approximately 20 QTLs for tomato plant height have been identified (Weller, 1987; deVicente and Tanksley, 1993; Jansen and Stam, 1994; Grandillo and Tanksley, 1996; Paran et al., 1997; Prudent et al., 2009; Zhou et al., 2016), but none of them have been finely mapped. In this study, seven QTLs controlling tomato plant height were identified through QTL-seq and single marker analysis (Figure 1 and Supplementary Table S2). Among them, *qtph1.1*, *qtph10.1*, *qtph11.1*, and *qtph12.1* seemed to be novel QTLs for tomato plant height. *qtph1.1*, *qtph3.1*, and *qtph12.1* were major-effect QTLs, and phenotypic variation explained by them were 15, 16, and 12%, respectively (Supplementary Table S2). These results laid the foundation for fine mapping of tomato plant height QTLs.

A new putative candidate quantitative trait gene for tomato plant height was identified in this study. The *qtph1.1* locus was further fine mapped to an 18.9-kb region that contained three putative genes (Figure 2 and Supplementary Table S3, Supplementary Table S4). *Solyc01g098390* encoded Gibberellin receptor SlGID1a. The *SlGID1a* gene in the NIL-*qtph1.1* plants contained an SNP, which resulted in conversion of the 83rd amino acid Arginine (R) to Cysteine (C; this allele was named SlGID1a^R83C^) (Supplementary Figure S3). The amino acid R is completely conservative in GID1s from plant species (Yoshida et al., 2018), suggesting it is important structurally and functionally. A previous study showed that the loss-of-function mutation of the *SlGID1a* gene by gene editing resulted in typical GA-insensitive dwarfism (Illouz-Eliaz et al., 2019). The NIL-*qtph1.1* plants also showed lower plant height and insensitivity to exogenous GA_3_ stimulation compared to NIL-WT plants (Figure 3). Similar to a previous study (Illouz-Eliaz et al., 2019), several genes related to GA biosynthesis and signaling were up-regulated in NIL-*qtph1.1* plants (Figure 4 and Table 1). These findings suggested that *SlGID1a* is the putative candidate gene of *qTPH1.1*.

*SlGID1a* might be a good target gene for the improvement of tomato plant height. GID1s play important roles in GA signaling (Sun, 2011), which usually causes significant changes in plant phenotype, especially plant height (Cantin et al., 2018; Cheng et al., 2019; Illouz-Eliaz et al., 2019). Several GID1 mutants have been utilized in agriculture. The recessive brachytic dwarfism trait (*dw*) in peach has little or no effect on fruit development. It contains a nonsense mutation in GID1c (Hollender et al., 2016). Two alleles of non-synonymous single nucleotide mutation, GID1c^S178F^ and GID1c^S191F^, have also been found in dwarf peach (Cantin et al., 2018; Cheng et al., 2019). Three *GID1* genes, *SlGID1a*, *SlGID1b1*, and *SlGID1b2*, are encoded in tomato genome. Among them, *SlGID1a* has the strongest effect on stem elongation (Illouz-Eliaz et al., 2019). In this study, a preliminarily phenotypic evaluation showed that the allele SlGID1a^R83C^ in NIL-*qtph1.1* plants had very little effect on flowering time and fruit weight (Supplementary Table S7), which suggested that it might has potential application in dwarf tomato breeding. To achieve the breeding goal, more field experiments need to be conducted to evaluate the effect of allele SlGID1a^R83C^ in NIL-*qtph1.1* plants on other important agronomic traits in the future. Furthermore, the *SlGID1a* gene may also be a good target for optimization of plant height using base editing system (Shimatani et al., 2017).

Plant height is usually controlled by phytohormones and their interaction (Wang et al., 2017). In this study, the SlGID1a^R83C^ allele in NIL-*qtph1.1* plants affected the expression of the genes not only related to GA biosynthesis and signaling (Table 1 and Figure 4) but also those related to auxin homeostasis, transporting and signaling (Table 1 and Figure 5). The homologs of these genes have been reported to control plant height. Overexpression of rice Indole-3-acetic acid-amido synthetase GH3.8 leads to lower free IAA accumulation and shorter plant height (Ding et al., 2008). Mutation of the transcription repressors in auxin signaling, BnaA3.IAA7 and BnaC05.iaa7, results in dwarf phenotypes (Li et al., 2019; Zhao et al., 2019). The small auxin up-regulated RNA (SAUR) genes in *Arabidopsis* play important roles in auxin-induced growth (Stortenbeker and Bemer, 2019). However, further study is required to determine whether these tomato genes related to auxin homeostasis, transporting and signaling also regulate plant height and the molecular mechanism of their transcriptional expression regulated by GA signaling.

## Data Availability Statement

The datasets presented in this study can be found in online repositories. The names of the repository/repositories and accession number(s) can be found in the article/ Supplementary Material.

## Author Contributions

XLL, JW, MY, KW, XYL, TG, XW, YG, JL, LL, and JS performed the experiments. ZQ helped analyze the data. WY, YD, and ZH conceived and supervised the study. XLL, WY, and ZH wrote the manuscript. All of the authors read and approved the final manuscript.

## Conflict of Interest

The authors declare that the research was conducted in the absence of any commercial or financial relationships that could be construed as a potential conflict of interest.

## Footnotes

1 http://www.ncbi.nlm.nih.gov/tools/primer-blast/

2 https://solgenomics.net/
