# Supplementary material for: SlGID1a Is a Putative Candidate Gene for qtph1.1, a Major-Effect Quantitative Trait Locus Controlling Tomato Plant Height
Source: Front Genet. 2020 Aug 7;11:881. doi: 10.3389/fgene.2020.00881 (PMC7427465; doi:10.3389/fgene.2020.00881)
Supplement: Supplementary file 2 [file Data_Sheet_1.docx]

Supplementary Figures

**Supplementary Figure 1** Pedigrees of the materials used in this study.

**Supplementary Figure 2** Sequence information of the *qtph1.1* locus and SlGID1a.

**Supplementary Figure 3** Multiple sequence alignment of GID1s based on full-length protein.





**Supplementary Figure 1.** Pedigrees of the materials used in this study.

**Supplementary Figure 2.** Sequence information of the *qtph1.1* locus and SlGID1a.

**DNA sequence of the *qtph1.1* locus in SG-7**

The exon and intron of *SlGID1a* gene were highlighted in purple and grey color respectively, the yellow highlighted 247th nucleotide of the *SlGID1a* coding sequence was C in tomato line SG-7

GTGATATAAGCATGAGGTTTGTCCACTGTATCTCAAATTAGAAAATAGTAAAAGAAACAATCCTAAGAAGATGGTGAATTTGCTATGTTTAATCCAGGAGCATGCAAATAGCCAAACCATAACCTAAAACACAGGCACGATACCTTGTGGACACTAAATGATCTAGTAGGAGGCATTTGGACAATATCTGGTTGAAGTTGAACAAAAAAGAATTTGAAGATGAAGTTGAAAAGGAGTATTTTGAAGTTCAGACATTTGCAAGTCATTTAAGACCCCTTAATGCTCTGCCTATGTCTCTCCATCCATGAACTGCAAGAGGCAAACTGAAACCCCAGTTGTAAAAGGAACAGCTGCTTAAGAGCTCATAATTGAGTGAGAAACAGCCATTTTATTCGCACAGCAGTCTAGATTTAAAAACAACTTCTAGTGGCCCACTGGATCAATCACACAAGCAAACAACTGGTGGTTGGTCAGTCCATTTCTACTGTCCAATTTGCCCAATCACACAAAAAGTCCAATTCTGCTGTCCGCCAAAAAAACTGATTTTGTAGAGCACTTCAAAGAAACAATCAACGAGTTCCAGTTCTTCCTGGTAAAGTGTACTTCAGAAAGTAATTCCCAATAGCTTTAACATCTTCTACAGCAACATTAAATATCAAAAGCCAATATGCAAATATCCGGATGAACAGAATTTATGTTAACCTAGTATACATGTCAAGGAAAACATAAACTGACATATCAATGCAAGTAATGCAACTAAGAGCATTTTAAGGTAAAAGAATGGAAGCATGCACAGAATATAGTCAGCATTTCAAATCACTATGAGATACAAGAGTTTGGAAACCAACGGTCAACTAGCATGGCAAAGGAGACAAACCTGTAAACTGTACCCAGAGAAAGCTCCAGGGAATTGTTCTGCCATGATTTCCATGGCTGCGAGAAAAGCTACCACCTAAAATAGAGCCAATCATACAGGTATACCTCCTGTTAAACTACAGTCTGGCCCACAATAACAACAACAACATACCCAGTGACAAAAGAAGAGCAAAATGTCTAAACCTGCTTTCCCATTTGTGGGGAGATCACAGCATAAACTTTTGAGTCTACCACTGTTTTGTACAGCTTCTCCCTATCTCCACCAGTTGTTCCCATTACAAAAGGCACTCCAACTTTACAGTATAATTCAGCATTATCTGCAGGTTCATGGTGGAAACAAGAACATTACATGGCTATGTCAATGGAATCTCCAGCAAAGAATGCAATATATATCATTTTGGTGCTTTCGTATTTCTATATCTCATTGCACACCCTCTACTGTCCTCCACTAAAGACATGTGTAAAAAATTATCACAGAAGCCTACCTATGCTGGCTAAATTTTATTTATAAGAACCTCAGAATCAAGATGATTGAATTTACAGACAAACTTAAAATTTTAGCATTTTACAGAAAAACATTATAAAACTTCACCGGACACCAAAAATTTTAAGTTGCAAGGAAATCTACATTTTACAAGAGAAATAGTCTATGTTTTCCTCTCAACACAACTGTGGTTTCTTTCTAACAAAATCATCCAAAATATTAATAAAGGAATGGTTCTCCATTTCTGCCCATAATACTTAGCTTCTGTTGGTTCCAGCTAGCCACTGCTTGCTCACAAACATCATTCACTGAATACCATAAAGCTTCCAAAACATACACACAATTTGCTAAGTTACAATGTCGCAAGATATATCATACAGACCCAGCAGAAGCTTCACAAAAGAGTTGCGCAAGCTGCTTTATGTTATTAACCATGCCTCCTCCAAAAGCTAGCCATTAAAAACATCATCTCTGTTAGGGATTAGGCTCTCCAAAGAATTTTCAGTATCCGCTGAGTAGATTGGCCAATTTCGAGTTTCTGCTCTTGTCCACATCATTCAGCCCCCCTCGCCTATTCTTGTGAAGCCACAGTTCTTGTAATTTGTCTACTTCCATTCCCAAGACTAAATATCACTTTGAAAGGATATGTTAAGACTGCACTTGCTTCTTCTAATAAGCTCAACCAATGTTGCTAATCTTTTTTTTTCTCTCTTTTGATAACAGATAAATCCCGAGTCTAGTAGCACACGCTTTGAAACTTGGTGGATAGTGTACCCACTACCTTTCTCCACTTAAATATCATGATTTTGGTTGAGGCAGCGTTCAAAGATGTGATTCGTGTCTAACCAACATACACGTGATGAACGCTGCCACTAGATCAAAGCCCAGGGGCAATGTTGCTAACCTACTGTTTATGATAACACGTGGATATGCTTCTGTCAAGATTGTGATTCCACACCATTAATAACGAAACAACATTTATAAGTAACAAATCCCTCTCGGAAGGTCTAAAGTAGTTCATAGAGAATGGTTGACCTTATTTTCATATGATATGTCCAAAAGTATATGTGCATTGACATCAAACACGAAATATCAAAACAATAAGATCCATAAATCTATAGCATATCATAACAGCCTAAAGATATTGGAAAATGACAAAACTCAGCAGATTCAAACTGAATTGCTGTATGTCATTGGCATATCGCCGTAAAGTATCGAATCCTTGTCTTACCATTCACTGCAGCAGGCAATGTGTAGTCCACTACAATTAGATTAGGGTGTTCCTCAAAGACAGAGGACAAGAGGTTTTCTCTATCCGAAGGACCATGCACAATTATCTCTTTTCCACCCGCCTCCACAGTTTTTCCTTCATCTTGTGGACCACCAAAACATATAGGCACAGGTTGAAGTCCCGCAGCGATGGATGCTTCAAGAACTGCCCTCCCCATTTTGCCAGTACAACCATTCACCTTTACATCAAAATAAAACAAACCTAACTCAAACAGTATTACCTTGATAAAACTAGAAATCAATTAACAACAGTGTAATTTAAAATCATTGGCAAACATATTAATTAAAAAAAAGGAAGGAACCTACCATTATAGGGAGTGGCTCGATTTTTGATTTAACTAAAACCTCAACATTTTGTGCTGGAGCAGAACACATCTTCACCGAATATTTTCGACCTCGAACAAAACTATTGCAGCCATTTTTGCTGCTTGAACTGTAACGCATTCCTCCTCCATTAATGTAAAGTGGAACTTTGATGATAGCCCACATTGTTTTACTGCAATACAATGAGAAAGCGCACACAAATTATAAAGATTTTGACTCGTCAGCAAAGACACTCTAGGGTTTTAAAAATTATCGGCCAAGTGTAGGTGTTATTTTAGTTTTTAGGACATTGCTATAAAATTTGTATAAATGTTGTACTTCATTAAGTTTAATTTTACAAAATGAATAGTATTATGGAAATTGTAAAATATTTTGTGTAAATCAAGTTCTGTAATAAACGATAGTAATAGAATTATTGGAGAAAATTAAATTGATTTTTTTAAAATAATAATATAACAGTCCTTTTGATACTTTAATTTTAGAGATTATAAAAATAAGTACCTTGATTTTATAATCTCAAAGACTTGCTCTTTGATTTATGAATCAATAAACGTAATAAAGATAAAATAATTAGTTTAAAGAAAAGGAAGATGTAAAAGAGGATCTCGAATCATTGTTCTTTAAAATAAAATTAAAATTCATTTTTAAAAAAATGAGTGTGTGTATATATATATATATGGAAAGTGAGAATAAGTATAAAAAGGGAGAGATAAATGCTTTAATTAATTAACAAAAAAAAGGGAAATTTATCTAATTTGCTTATCCACTCTGAAAAATAAACCAAATCCAATTTTTTTTTAAAAAAAAAACAACATAAAATTAGTAGTAGTAACTTAGATTCGTGGGCCTTAATATATTGGGGCCTAAACCCAAGGTTTTAACAGCGTCGTGGTCTGGTTGGCCCGCTATACTTTATAACGGTATATTTTATTTTATTTTTATATGAGATTTTTTTTATAAAAAAAATATATGAGATTTAAAATACTAATTAACAAAAAATATATATAGATAATGTCTCCTTTTAATGAAGATTATCAAAATGCTACATTATATTAATAGTATTCAATATAATTTTTAAAAACAAGGGCTCTAATCATTGATTAGAGCCCTTGTTTTTAAAACTTGTGTTTATTTTGATTCGTGATTAATTCATACAATAGCGATCAAAAACAAATAGGTATTCAAAACAAGTAAATGTTTAATTCAGTTATATTTTTAATTATGCTATTTTTATTCATGAAGTTTGACCTTCTCTAATTTATCTGTTTTCTTTATTATCTTTGCATTTTCTTTGAAGACTTAAATTCAAATTTAATTTGTCTGAAGATCAGAAAAAATTATATCAAATACAGTATTTGAAATGCTGAATTTCATACGAAACAACCAACAAACGGAAACTATTCAACCCTACTCATAATCAACAACCCAAACGACCCCAAAATTGTTGTTTGATATATAGTGATCTTCTTAACATTTTGCAAACAAAAATATGGAATAAATATAAATAAATATGCGACTAATAATTGTAACAAGATTTAATCCCTAATTTATATGGTCTTTCACCATTGGATGTGGTCATTATGATCAAGCACTATTACAAGCTGGGAAAGGGAAAGGGACAATCGATGGATATTTAGTGAAAATATTACTATAAAAAAATATTTTGATATGACAAAAGAAACAACTGGCTAGCCTTATAGTATAATAAAGAAAGTAGATGATTTGACCCTTTCGTCATTTGTTAGCTAGCCCCACAAAGTTACGTAGATATTAATAATGTAGTAATGATAAAAGAAATAACTAGCTAGCCTTATAGTATCATAATAAAAATAGATGATTTGATCTTTCGTCATTCGGTAGTTAATCATATAAATTATGCATATATTAGTAATGTATATTAAGTATTAGTTATATAGGGTTTAATTATGTAAGATTTATTTATACATACATATTATATAGATATTAGTTATCTAGAGTTTAATTATATGAAATTATTATACATTATTGAGCAATTTTTATTTCTTAATTCCAAAAAAAATTAGCAAGCTAATATTTATTATGACCATTAAAATAATCAACTATATGAATAACACGTTAAAAAAAAGGCAATTTTCACATATAGCAAACAAAAAAATCATATTTGTATAATATAACAAACTTTGCATAATTGCGCTCCATAGCAAAAATAAAAACTGTATAATTCGCTATACATATAAAAGTGTATAATTCGCTGGCCTAAATTGTATAATTCGCTGGCCTATTTCGCTGCAATTGTATAATTAGCTTTGCATACAGTTAAATCGAATTAAAATGTATGTATATTGCATAATTATAAGTGTATAGCAAGAAGATATATGTTTCACAATATATACACTTCTGTTGTATAAAGCTAGAAAAAATTGTATTTCACTGCAATTGTATAATTCTTTGCCCTTTTTCTCTGCAATATTTGAAGTAAAATGTTTGTAAATTATATAATTAAGTGTATAACACGAAGATATACATTTTTGCATGTGGATATACAATTTTCTCTCGCTTTATACAAAAACAGAAACAGAAATTATACACTTCTGTGTATAAAGCGAGAGAGGCGAGAATGGGAGAGTGGCGAGCGAGACTTCTGGGAGAGAGACGCCTGACAAATTTTTGCCAACGTTTGCTATGGGGCACAATTAAATCAAACCCTAACTATTCCATTTAATTTAGGTTATTAGTTTGCTATTTTATACAATTTTCCCTTAAAAAAATACCAATAGCTAAAAATAATAAAAATAAAATGATAAAGGTAATTAAGTAATTGGTATTCAATATGATTAAGTATTTAAGTAATAGGTTATCTAAGTTATTTTTTTTAAAAAAAAAAAGAAACTAAAAAAATTGAAATGTGTATTGATGGTGCGTTGTAAAAAAACTATGCAGAGAAATTAATTACAAATAATAATCACTGCCTTGCCACCAAAGATAACAATTAACAAGAATTTAAGGAGATAAAATTATCAAGCAGAAAAACTCACAGTGCTTTTTTTTTTTACATTCATTAAATATTTGATTTGTTGTAGATTAATACATATTTAAAAGTAAATCGATAGTGATAGATATATTATTTACTATTTTAACACATATATAATTAATACACCATAACTTATTCCATTTTTCATCCCGCATAAGTTATATATAAATTTTTCATAAGTTATACAAATATTAAATATTGATTATGTAGAATTACAAAAAGCGCAAATAAACACCGTATGAAATTAATACATGAATAATTTTTATATAATATTTAAAAAACTACCAACCAAACATTGTATAAAATTAATACATAAATAACTTTTATATAATATTTGAAAACTTACCAACCAAACGTTAAAATTAATTCATGAATAACTTTTGATTCTAGTTGCAAAACAAACATTATATAAAAATAACACATAAATAACAAAGTAATTCATATATTAGATGTAGCTTAAGCCTGCTATCAAATGGCCCTTAATGTCTCCTTAAAATAAAGATATGAACCTTAAAATGGCCTCATTTTATATATATATATATATATATATATGTTGGAACCAATAAATTTGTGGTGGAGTGATATATTTTTCTTTTATGTGTAATTAAAGATCTCAAGTTTGAGTTCAAGTTTCTCGATGTATGAAGTTGTAGTTACTTGTTTCAATTTTTGCCATTAAATCAATGACTCACCTTGTTAGAAATTTTCAATAAATATCTTTTATATATTTGATGAAATTTTCAATATAAATATAAAATCTACAGAAAAGACATTAATTTTCCAAAAATTCATAACCACTTGGATCCGCCCTTGCCTCATAGTTTTGCTAAGTTATTATAATAGTATTATTAACTTATATCTATACTTAATAAAAATTCATAAAATTCAAACTCCAAATCCATCCTAAAATCATATGCTAAATGGAGATCATAGAGGAGGAAACTTGGTGAGGAGACTAATAATTGGGAAGAAGGCAAGATTGTTTTAGAATGAATGAATGAAGTAAAAAGGATTAGGTGTTTAGTTTTTGTCCACAACATGAAAGACCAATATGAAGTACAGTGAGGTGCAATGCCACAAAAAAAATAAATTGAGTACTCACTTATTTCCAGCTAGGAGCTTTAATGCCATTTGTACTTATATTCCATCAATCATTTCTACCTACTTCAACACTTATAATGAAACTTGCAACCTGTTTTCTTGTCTACAGGATGTTTCTTTGGCTTTTCCAATTCATTTATATGACATTTTGTTACAAAAAACTACACAAGAATCATATGCACAGAGTAAAAGAACTAGGTCATTTGCAAACAACTGAAAAGATAATTGTGAAGCAAGTTTCAAGTGAATTTACTATGAGATAAACACATGAATTGATAAATACAAATTCATCATGGCAATATTGTTATGCTTGCACAAAAGGAAAGAACTTTGAAATTAGGGAAAATTGTGTGAAAAATCGAAGTAAATAAAGGAGTCTTAGTCTTATTAAATTCGTTGACCAAATTTCAATGAGAGTTGGAGCAAGTCAACAGAATCACAATCATCACTACTCCTATATAAAACATCATTTTGATGCTACTAAACACATGTTATTTCACACAAACAAGGACTTGATCTTGGCAATAACAATTATTGTGAGAGATAGAGCATAATAATTCAAACTCATCAACAAGTTGTTGAGGTTGTTGAATCCATCCCATTTTTTAGTCAACTTTAACTATTCAATTGGCATAAACAATTGTTAAAAAGGAATAGTAATTGGAGTTGAAAAATAAAATATTTGGTAGTTGACAAATTGGTAAGGTTTGCAACAATTCTTGACTTTGTTATATTTACATATGTCATTAGTGACATAAGCATGGTTTTATTCTTCTATAAATAGAGCATTCTTGCTTATTTGTAGAACACACCAAGTTAGAGAGAAAAATCATTTTGAAAACAAAGTGAGGTATTCCATAGATTATACAAATAAATAGTCTATGAAAAAAAATAAAATGTGAGCGATATTTTAGTAAGATGGAAATCAAAAGAGTACTGAACTTTTTGAGAGTCTTACCTTCTAGAATAGGAGGAATCATGTTGTCTTGAAAAAGGTTGATGCAATTGGTTAAATTGTAAAGGTAGTCTCTTTGATTATAAAGTTAATGACTTCAATTTAAGTGAATTTTAGAAGTAAATCTTAAGAAAGTAAGTTGTAATTTATACTTTCATAAGTAAAGTATTTTTTTTATCATAAAGTTTCTTGTGTTATTTATATTTCATAATTTATTTATTATTTTGTGACGGAAGAGTCCAATTTTTTTTTTTAGCCCACTGAGTGGTTGCTAAAATAACAATATGTTTCCCAACTTGTTGATTAATTTTATTTTCATTTCTAGAGTTGTTAATGTGAACTATCATATTTTATCCAAGTTAGTTTATACATGTTTTAAAATATGATGATTATGTCAAGCTGGTTCATGTTTTTGTATGTCAGAAAATAGTCGGTCTATCTCATCAGTATGTGGATTGTGGGCTATGTCATTTTTTTAAAAAAACATATATTTTTTAATTAAATTATTAACGTAAATATCGATAAGATATTTTCATTTTAATCTATATTTTATATGCAAATTCAATTATTTAAACATTAAAAATACCTAAATTTAAGAACTACATATAATAATTAAATGTACACAACAAATATTAATAAGTTTGAGAAAAATCATATCACCGGTTCCGAGTACCTACAAATATTTGATTATTTTCATGATGAATGCATGGTAAGAACACATCTACCAATGCTGAATGATGATTCTGATGATACAATAGTAATAGAAATAGGTACATCACGTTTGATGATTTTAAATTTCAATATTTCAAACTTTTAATAGCGGTTGAGTTTGAACTTTTGCAATTTTTAATAGTTTATTGTATTTTTAATTTTAATCATCTCTTTATTTAGCTCACAGACCAACTCAACTCATATTACTTAACGCACACACACCAACATAATTATTTGGGTTGAATTAAAAAGTTTCTTTTTTTAAATGAATTCCCAAATTTTAGATCAATCCTATTGAATCACGAGTTAGATCAGATCAATCCAACATATCTTTCCTTTCATATTTATACTGACATATTTCCCCTCCACTTTAACTTAATTATAAAATTAAATTTTGTTAAATGACATAATCATAAAAGACCTATATCGGTCGGCCCCATACGAAGACATATTTAATGCCACACGCATACCCACCCCTCCGTTTCTTGTTTGCGGAAAGGCAAATATTTTTGGTAAAAAACGTAACTAATTTTCTCCCTTTCTTTATCCAATTTAGGCCACACCCACATTTAAGAAAACTCCATGGGTATATGTACTTCATTTTTCTCAAAAGGGATACGTGTACAATAACATACCAAATAATGCATAAAATGTATTGATAAAAATAGTATTTTAAGCCAAAATAACATAAACGTAACAAAAATAAGAAAATAACAAGATGATACACACGTGTCGCATCCGTAGGTCCGTTTGTCGTCACGTGCCTTATGGCGTTCCCCCCACGCTTTTGTCCCTCAAAGGACGCCGTTCACTACTTTTTGTCTTTTTTTTCCAACAATTTTGGCAATAAATCAACACTCTCGGGGCCATCTCATGAATACCCCATACTTTAAGTCCTCATCCCCGAAACCTAGCAATTTGCTAAATATTACTATCTGGGTCCTACATTCCTTACCCAAAACTTTATTACTCCTTTAACCCCTCAATTTGTCGTTTATCTACTCTACCACCCTGTGTGTACTTTAGTGTTCGCCCCTTTCTCATTGCTATTCTGCGCCTCGAATCCTTATCCTTCACCTCCCCCTTCATTTTCTTGTTGGTTCATACCCATTTATTTCTCTCCTTGTTGTTAATGAGCTGAAGGAACAGTGTGTCGAGAGGGAGATGAAAGTACTACTGACAAAGAAAGAAGCTTTATTGTTGTTGGGGTTTTTCATGGAAATGTTCTTTCTTGGTTTTGTTTAATCGCTTGTGCTTTTTTTTTTTTTTTGCTTTTCACCAAATCAAGAAAAAGGAGAAGCGGCTGTCT**ATG**GCAAGAAATAATGAAGCTGTTGCGAATGAATCCAAGAGTGAATCTAAGGTACTCCGCTTTTTTCCTGTTTTCTTTCTCTGATCATCAATTTGATTTCTGTTGATTTATGGGTTGGCTTCAAGATTTTGGGTTTTACAGTATAGCTATGGTTTGGAGTGATTTTGTCATGCAGATTTTGAGGGGTAGGGTTGTGTTTTACTCTGAATTTTGAAATTTGGTACTTTTGAAAGGATGTTTTTTTTTGCTTCTATTTTTGGTCTATCAGTGCCCTGTACCCCACAAATTTTACTTGTTTTTACAGGAAAAATGAGTGCCTATTTGTGTAGATTTTGGATATGTATACCTTGGTGGTGTGGGTCTGGAGTTGCTCTAATTGTTCCCCTAAAATTTGAAAACTTGCCCTTTTGTATGATGGGTCTGAATTATGTTTAGCTATTCTTCTTTTAGTATGCTGCACTGTCTTGTGTTTCATGGCATATGTATAATAGTTTGGCTGATAGCAGCACGTTCTAGTGTTATTTACAGTAGTTCTGCTGTGTGTGTTAGATGTAATTATGTAAATTAAGGTTATCGTTATGCATATATCATGGCTTTTATCACTTCAAATTGTACAATTTCTGAAATTTTACCTCTGTTTTCTTATAGAGAGTGGTTCCGCTCAATACATGGATCCTAATTTCAAACTTCAAGTTGTCTTACAATCTTCTCCGTCGCCCTGATGGGACTTTCAATCGTCACTTGGCAGAGTTCCTTGACCGCAAGGTTCCAGCGAATGCAAATCCAGTTGATGGAGTTTTCTCTTTTGATGTTCTCATTGATCGTGAAATAGGCCTACTTAGCCGTGTCTATCGGCCTTCTTTTGAGGATGGAGCTTCACCGAATATGGCTGAACTTGAAAAGCCTGTGACTGCTGATGTTGTACCTGTCATAATTTTCTTCCATGGTGGAAGTTTTGCACACTCTTCTTTCAATAGTGCCATCTATGACACACTTTGTCGCCGCCTTGTTGGCATTTGCAAGGCAGTTGTTGTGTCAGTTAATTACAGGCGAGCTCCTGAAAACCGTTATCCTTGTGCTTATAATGATGGATGGACAGTTCTTGAGTGGGTTAACTCAAGGGAATGGCTGCGGAGCAAAAAGGACTCGAAGGCTCACATATACTTAGCTGGAGATAGCTCTGGTGGTAATATTGTTCATAATGTGGCTTTCAGGGCAGTAGAATCCAACATAGAAGTGTTGGGAAATATACTGCTGAACCCTATGTTTGGTGGACAAGAGAGAACAGAATCAGAGAAGCGATTGGATGGCAAATATTTTGTCACACTTCAAGACCGAGACTGGTATTGGAGAGCTTATCTTCCTGAAGATTCAGACAGGGACCATCCTGCATGCAACCCTTTTGGTCCAAATGGTATAAACCTCAAAGGCGTCAAGTTCCCAAAGAATCTTGTTGTTGTCGCAGGTTTGGACCTTGTTCAGGATTGGCAGTTGGCTTATGCTGATGGGCTTAAGAAGGCTGGACAAGAGGTTAACCTGATATATTTGGAGAAGGCAACAATAGGGTTCTACCTGTTGCCAAATAATGAACACTTCTACACTGTCATGGATGAGATAAGTAGCTTCGTGAGTTCTGACTCTCAG**TAG**ATTTAACCTTGTCAAAAGTAGGATATGCTTTGAAGACGTTTGATGTTTTGTTGAAGTTAGTTCCTAGCCTGTCAACCGTTTGAAAGATTGTATAGCATCATCAATTACTTCCTTATTGATCATGCTATTGCTTGGATTCTGCTCCATTGGCTGGGTTGGTTATTGGTGGCGGAAAACCTCAATCATGTAGCTGGATCTGTGTTATATTTATTCCAGGTACAGGGAGTGTCTGGATATTGGTTGTATATTTTGGTAGCTTGCGCCAAAGGTTATAATCATCTTGTCTTCGTCTAGCAGATATGCTGATTTACAGCTAACCTTTCCCATGTTAGCCTCCGTAGTAGAGGGGGTAAGTCTGGTGCTGATCCGTCAGGGGACCAGTGCCTTGCTAATGTTATATAGCTGATCATACTATTATGCTTCACGGATGAACATCCTAAATTGTGAAGTTGTATAATATATCTATAATTATATAGAACTATGTTTGCTTCCTCGCGTTATCATGGTGTGTCGCCCTTTGGTTTGTTTTCTTTTTTATGTTCTTTGTCACTTCCTTGACCTTCAATATGTACATCCTTAGATCACTTATATTTAGAAGATATGTTTGCAAGTACTGTTTCTTTTCTCAGGGATGCACAATATGAACATATGTAATTCAACATGCTTCAATTGTTATAAGTGGAATTGTTGTTCTCTTGCACATGAACTGATGTGCTATGAGGTCTTTCTATATTGCAATGTTGGCACTGTCTAATACATGAGGTGACTTTTTTGTTGGTCAACTGGAAAGTGTTACTTATTATGATTAGTGGAGTGTAGTTCTGAAAAAATTATAGTATTTGAATGTAAGTAGCGCGAAGGTGGAACCTATTCATCAATTAAATGAGTGAAAATCATACTAAACTATATGTTTGGCCACAGATTTTGCCTCAACCAAAAAGATCTCAAAGAAGTGTTTGTCTATCAAATTAAACCATTATTTCAAAATATCGTTGGACAGGTTCAAGTCGAGGTTTGTGTAAGCTGATATGTCCTCAACAGAAAAAAGAAAAAGAAAAAAAAGAAGCTTAAATACATGTGTAGAAGCAGTGGTGATTTGAGAATAGAGACAAAATAAGCTGTCTGTCTTTTGGTGACCTTTATGAATCTAGTCCTATAGTGTCAAACTAGTGAAGAACTCACTGGCAAAACTGCTTCTTTGCTCCCTTCCCCTTTCTATTTAGTTTAAATTTAAATAAATTATATATGGCTCAAGATAGTGGCGTTTTCTTTTATATAATTTGAGTAAAACAAAAAGATTTTGCAAATATGTCATGTTTAAAAAATGCTTAAGAGGGATTTAACAAAAAAAAAACAAAAACGAATTGACTGGTCACAGACATAGATTTTGGAAATATGGATACAGATATTGACTTGTTAGGAGAAAGAGTCCAGTTGGCGGTGATTGCTATTTGGATGTATTGAAGGGGACCATTTTTCAAAACCAACAAAATCAATCAGGTGAACTTTTGTTTTTTCTTTTATTTTCTTAGAGACCAAAGTGGGTGAGCTCACTATTTCTTTAAGAAAAAAAAAAACGTGAATCTGTACGCGAATCTTACTCTTATCTTAGTGAAGCTAGAGAGGTGATTAAGAAAAATCATGCATCAAAACTCGTATTTGTGTCTAGTCAAACAATGTCATAACACATAATACTCATATTTGTTTTGTCTATTTTTGTGGGTGTCATTTCATGAGGTAAATGTAAATGAATGAAAATTACAGTTTGTAAGAGTGGAAGTCATTGTCATATGGAAGAGAAAAGGTGTCTAATCATGGATAAGGGAATTCGAGTGCTTTTGCGTTAAAATAAAGGGAATAAATAATATTCTGACTATAGAAAAATGTTTGAAATTAGAAATTGAAAAACAGAATAATATTACTATAAAAATAAAAAAAGAACAATATTTGCTACTTCATGAATCATGAACAATAATATATATAACATATAGTATCAAGTTCATTTGATTGAATTGTACAATTTGCCCTTCAAATTGGTTGATGTTTAATTCTTGCCCTTAGCAATCGAGACATAAGTTCTTTAAAAGCGCGCAATAGAACAAGTGGGGAATGACTTTACAAAATCGTGACTATTTTTCAATTATAGACACGTATCGTATTTTTACCCTTTTTCTGGAATGTGGAGTATTTGGGCTGTGAAGATGTGGCCCAAGTATCCTCCACTACATGGTCCTAACTAGATCTAGGCCCAATTCAAAACGCATGAATAAAGGAGAAAATTGTGTATATATAATCAATGAAAAATAAATCAATTTATAATGAATTTTCATAATACATATTAATATACTCATATATCACATGTTATACCAACATGCATGCAGGTATGACGAAAATGACAAAAGGGTTCCATCAATATTCACACTTATATGTATAAAAAATAAATAAATAAGATAATTTCACTAACATCAATGTTCAGATGTATACAAAATAAAAAAAAAGGCCAACAAAGTCATCATTGTTTTGTTATTATTGTTGTTGTTATCGTTCTCCCAGAAAATTATACTAATAGTATAATAGGTAATGGATCACATGCAATTAATATTAGAAAATATATTGTTTGGCTGTACTTTGCACACACACCTAATAGTAGAGTAAGAAATCATCAATAGCCAAATCATAACTTAGTTGACCTTCAATTTTGAAACCACATATGTTCAATGAACCCACAAATAAATAAATAAATAAATATCAACTTTATTTATTTACTTTTAAATAATTTGGAGTAGCAAAATTCAAAGATAAAGAAAAAGAAAGTAGTGTTTCCATGAGTACAAGTGGTCCTAAGAAAAAGACAACATAACTCATAATCGTAAAGTAGAAGAATTAAATTTTATAGTAATGCCAAGGAGCAAGGACGGTGCATCAAATCGAGAATATATCTAATTTATAGCTTGATTTCTCACATTTTAACTGTAATTTTAGCTTAAAAAACTTATTTATAAGTTGATAGTTGTTTAATTATATGCTTAGCACTACAGATTTACAGACAATATTTAAAGAAGATAAAATAATCTCTCTTTAGACTTTAATAAATAAGATTACTGAAGACATATACTATATCTTATATTTGTCTTCGAACTTGGTTAAGGATATGGTGCACACAATTTTATATTTTGTGTTTAACTATTTTTTTAGAGATGCGGTTTGTTTCTTGATAGAATAATTCGAAAGATTATTATACATCTATTTTAATTTGACATCTCTAACATTTGAAACTATTCAAAAATTTTAGTGACATCTTAATTCGATATTAAATGTACATTGATCATGCATATAATCAAGCATTATTAAGTAGAGATTTAACTTATTTACAATAATTCTAACAAAATTAAAGATGATAGCTACACATAAACTACATTAATGAAGTCCTTCTCATCTTCACTTTCATTGCTTATACATTTGAAGCCAATATCATGAACACAATCTAAATTAACATATCCAAATAACTTCTAATTAAAGTTGCCATTAATTAAACATAATTAAATTCCATATATCTCATCAATCAATATTCACACCACTGGAAATGATCCTCCGGCATCCACGAATTGTTTCTCCAGCTACAAATTAATCAATTAATTGCAAAAATGAGTCCAAAACACCTCTTCCTCACTTGTTATACAATTAATTAATCATATATAGATGAAAAATGAAAATCACCTTAAATAAAGTTTCAAGCTTGTTCTTGACAAGGGTGTACTCAATTTTCACTTGTTGCAAACATTTTAAGCACCTGCATTCAAATGATACTATTTAGCTTTATTAATTTCAGTAACAACGTATCTAATACAATTTCATAAATTGAGTCCGCAGAGTGCGATATAAACAGATTTTACTTTTAGATTATATAAAAAATTGAAACGAAAACACCAAAAAAAAAAATTAATTTAAAACCTACCCTTCAACATTGCGTTCATCACAATAATTACGGAAGGAAATGCACACTCTTTGAACTCTTTGTGCCCCAATGCTGTAAATTAAAAATAAGAGTTTAAATTATGTACCCTCAACTAATTAGTTAATATATAAAAAAATTATATTATCAAATTTTATCTAATAGATAATTAGAGATACCGTGAGTTAAAATAGGTTTAATAATAGTAAAAAAAAAAAAAGAAAATCTTAATATACAAAAAAAAATATTTACCTAGAGCTGCTACCTTTCAGCTGATGAACATGAGCATCCAGTTTCTTGAAGTCTACATTAGACTGATTGCTATTTTTACAAACAAAAATTGAAATATTATGTGAGCTTGCTTATATTGTCGATCTTAAATACAAATAAATGCGAAAGCTTACATCTAGCGTAAAACTTGTCAGTTCATAATAAAACAGAGATAATAAGTTCATAAATATGTTTTTCCACCTACATCTCTTCCAGATTTGTATGTAAAAAGGGGAAATTAAACAAATTAATTACTCACAGAGCTTTTGCAAGTTCATTAAGAAGCCTTTCAGAATCTTCAAAGAAAAGGGATACAACTTCCACCACAAAATCAGGATTGCTCTCATCTTGCAGTTGCTGAAGTTGTATAAACTGTTCATCCAGAATTTTCTTCTCACACCCAAAAAATTAAAAAAAAAAAATCAACAAATCATAATAAAACAAAAAATTCAATGCACAGAACATTTCGCACATTGTTTTTTGAAAAGGGAAGTAGTTAAAACAATTTATTTTACTGTTCGAACTCGTAACCTATAGTTCACTCAATCATATGATAAAAAGTTTTATTTACAAATTTAGAGGTTAAGATTATTTTACCTCATTGTACAAGGAAGCAGTGTACTCAGCTAGTTTTCTTTGGATTTGTTCCATGATTTTGCTAAATTCAAGAGTGACTTTCACAAATTAACAGCTTGCTATGCTATTAATGAAAATTGAGAAGTTATATATACTCCTTATTTGTGCATGGAGTAAATTTTTTTTTCCTTGGAAAAGCTACAGCAGGGGCACTCTTTTCTATAACAGAGAAGACTAATATTCAATGAATTTTGCGCATTGATTCACTGTATTTATATTGTCACACTTTATTTTAGTAATTTTTTTTCACCCCCTCACTTTCTGCAATTGTGCTTTTGTTCCTTGTGCATCCTAAATGTCTCCCCTTATTGTTGTTTTTTTGGCAGTTATCTATTTCATTAAAAATATAGTAAAAGCTGTCACAAGATCCAAAATATTTTTTACCATACCCTATTTATTTGTTTCTTCTTTAGTCCTTTTTGTTTTATACTAATTCAATCTCGTATTTCACAGCTTAACTTATTTGAAGTTGAAATTAAAAAAAAAATCGAGAGTACGTCAAGTGAAACATAGTGAGATAATTTAATATTATTAAGTGAAAATCTACAATCAAATACAAATTTCAGATTTTTTATTCTTTTTTCTATTTCAATTTTAAGAGGGTTGATCCTGTTTGTTATTAATTTCTTTATAATTTTAATTAACTATCGAACAACTATCGAACAACTTCTATGTATTTATTTATGTGTATGTTTAGTATTGCTCGATTTGAGACTAATGAGAACGTCTTATATGTACCATCCAAAATTATGGTGAAATAGACAGAATCCCATTTATAACTATCTTTTTTGATGAATCTGAACAATTTGTAAAACATGTACATAGTAAACTATGGATGAATAAATTATTGAAATATGATAAAAGGAAAACTTATTTATTACATATGAAAGTGTAGCAGAATCCAATGTTGCACTGCAAATTGGAATATGTTTAGTTTTTAAGATGGAAAGGTTGCAAGTATGGGCAGGTCAGTATAAGGGGTTGGGGACAGAGAGGTGAAGCTGACCACCCATGTTACATCAATATCTCCAAGATTTAGTTGGTAGATTCTCTCTGCATATTCTCAACTACTATCAATTCTTCGATATTTGAAATTTACTGATTTGATTAATAAAAATATATTATATTAAATTTATTTACATTCAAATAAAAAATTTATGATTTATGATCCTAATCTGCGTCATTTGAAATAATATACTATATTGTGATGATATTGAAAGTTTAAAGTAGCGTGAAATAGCGTACTGTGACAACAAATGTGTCGTGTTTGACATGTGAAAGTGAAAGCCAAATGAAACCATCTTATCTTGGGATCAACTTGTTTCTAGCTAGGTAAAAGTTAAACAACAATCTTATCTAAATCCAAATGAAATATCCCTTACCTACTCTTTTCTTTTTTGTTCTTTTTTAATTATTAATATTCCCTCCGTCCAATATTATTTATCATGTTACGCTTTTTAAAAGTTAATTTGATTAATTTTCACGTTAAATTAGATTGCATTAATTCGATATTTTAGATAAAAAAAAATAGACATTATATGAAAAATACTATAAATTACAAATTTTTTACATGTTAATATGATAAAAAAATACATCTTAAAATATTAATTAAAGTTTTTATAATTTCACTCTAAAAATGAAAATCATGACAAACAATATCAGACGGATAGAATACTAAATAGAGGTGGTGATATAACTATATTAACCTTTGATATATTTCCTGGGCGCAATTTTACTTATTCAATATTGATCCAGCACATTCTCTGAGGAGTAACAAATAGTAGAATGGCAATTGTATTAACTAATGTTTGAGTAGAAAAAGATCAATATATTAAGAAAATAGTATAATTAAGGGATAATGCATAAGTATCCCCTCGACCTATACCCGAAATCTCAGAGACACACTTATATAATACTAACGTCCTATTACCCTCCTGAACTTATTTTATTAATATTTTTCTACCCCTTTTCGACTTACATGGCACTATCTTGTGGGTCCAATGCTAGTTGACTTTTTTTTCAAGTTAGTGCCACGTAGGACGAAAAGGGTAGAAAATTACTTCTAAAATAAGTTCAGGGGGAGAATAAGACCTTAGTATAGTATAAGTGTGTCTCTGAAATTTCGAACACAGATTGAGGGGGTAATTGTGCATTTTCCTATAATTAATAATAAGAATAAATTAAAAACAAAGTAATAAATTTAATTATCACTAGATTTTTTGAACTGGATAAGTAAAAGACAAATATTTATTTTTGGAATATAAAAGATTGGGAGTAGTACATTCGTATTTTTGGAATGATAATGGTAAATAATAAAGATAAATTGAAAAGTAAATGATGATTAAACTATTTCTTATTTTTAATTAAATAATAATAAATTATAATATATCAACAACTATATAAGGATTAAAAGGGAATCTGTTTCTGGTCTTTTTGTTTTGTTATGTTGTTGATATTTGGTTGGTTGGATCATTGTTTACTAACTTATTGTTATTAAAATTATCTTCGAAAGGGATGATTATAATTGGTCCCATATAACTATATGATTGTTGCCTCCACAACAATAAGAAGTTAAAATAATTACCTCTCTTTCTAATATAGTAACCCTCCCCACATAT

**DNA sequence of the *qtph1.1* locus in TS-165**

The exon and intron of *SlGID1a* gene were highlighted in purple and grey color respectively, the yellow highlighted 247th nucleotide of the *SlGID1a* coding sequence was T in tomato line TS-165

TTCTTCGCAGACCAGTTACTTCATATTTCAGTGATATAAGCATGAGGTTTGTCCACTGTATCTCAAATTAGAAAATAGTAAAAGAAACAATCCTAAGAAGACGGTGAATTTGCTATGTTTAATCCAGGAGCATGCAAATAGCCAAACCATAACCTAAAACACAGGCACGATACCTTGTGGACACTAAATGATCTAGTAGGAGGCATTTGGACAATATCTGGTTGAAGTTGAACAAAAAAGAATTTGAAGATGAAGTTGAAAAGGAGTATTTTGAAGTTCAGACATTTGCAAGTCATTTAAGACCCCTTAATGCTCTGCCTATGTCTCTCCATCCATGAACTGCAAGAGGCAAACTGAAACCCCAGTTGTAAAAGGAACAGCTGCTTAAGAGCTCATAATTGAGTGAGAAACAGCCATTTTATTCGCACAGCAGTCTAGATTTAAAAACAACTTCTAGTGGCCCACTGGATCAATCACACAAGCAAACAACTGGTGGTTGGTCAGTCCATTTCTACTGTCCAATTTGCCCAATCACACAAAAAGTCCAATTCTGCTGTCCGCCAAAAAAACTGATTTTGTAGAGCACTTCAAAGAAACAATCAACGAGTTCCAGTTCTTCCTGGTAAAGTGTACTTCAGAAAGTAATTCCCAATAGCTTTAACATCTTCTACAGCAACATTAAATATCAAAAGCCAATATGCAAATATCCGGATGAACAGAATTTATGTTAACCTAGTATACATGTCAAGGAAAACATAAACTGACATATCAATGCAAGTAATGCAACTAAGAGCATTTTAAGGTAAAAGAATGGAAGCATGCACAGAATATAGTCAGCATTTCAAATCACTATGAGATACAAGAGTTTGGAAACCAACGGTCAACTAGCATGGCAAAGGAGACAAACCTGTAAACTGTACCCAGAGAAAGCTCCAGGGAATTGTTCTGCCATGATTTCCATGGCTGCGAGAAAAGCTACCACCTAAAATAGAGCCAATCATACAGGTATACCTCCTGTTAAACTACAGTCTGGCCCACAATAACAACAACAACATACCCAGTGACAAAAGAAGAGCAAAATGTCTAAACCTGCTTTCCCATTTGTGGGGAGATCACAGCATAAACTTTTGAGTCTACCACTGTTTTGTACAGCTTCTCCCTATCTCCACCAGTTGTTCCCATTACAAAAGGCACTCCAACTTTACAGTATAATTCAGCATTATCTGCAGGTTCATGGTGGAAACAAGAACATTACATGGCTATGTCAATGGAATCTCCAGCAAAGAATGCAATATATATCATTTTGGTGCTTTCGTATTTCTATATCTCATTGCACACCCTCTACTGTCCTCCACTAAAGACATGTGTAAAAAATTATCACAGAAGCCTACCTATGCTGGCTAAATTTTATTTATAAGAACCTCAGAATCAAGATGATTGAATTTACAGACAAACTTAAAATTTTAGCATTTTACAGAAAAACATTATAAAACTTCACCGGACACCAAAAATTTTAAGTTGCAAGGAAATCTACATTTTACAAGAGAAATAGTCTATGTTTTCCTCTCAACACAACTGTGGTTTCTTTCTAACAAAATCATCCAAAATATTAATAAAGGAATGGTTCTCCATTTCTGCCCATAATACTTAGCTTCTGTTGGTTCCAGCTAGCCACTGCTTGCTCACAAACATCATTCACTGAATACCATAAAGCTTCCAAAACATACACACAATTTGCTAAGTTACAATGTCGCAAGATATATCATACAGACCCAGCAGAAGCTTCACAAAAGAGTTGCGCAAGCTGCTTTATGTTATTAACCATGCCTCCTCCAAAAGCTAGCCATTAAAAACATCATCTCTGTTAGGGATTAGGCTCTCCAAAGAATTTTCAGTATCCGCTGAGTAGATTGGCCAATTTCGAGTTTCTGCTCTTGTCCACATCATTCAGCCCCCCTCGCCTATTCTTGTGAAGCCACAGTTCTTGTAATTTGTCTACTTCCATTCCCAAGACTAAATATCACTTTGAAAGGATATGTTAAGACTGCACTTGCTTCTTCTAATAAGCTCAACCAATGTTGCTAATCTTTTTTTTTCTCTCTTTTGATAACAGATAAATCCCGAGTCTAGTAGCACACGCTTTGAAACTTGGTGGATAGTGTACCCACTACCTTTCTCCACTTAAATATCATGATTTTGGTTGAGGCAGCGTTCAAAGATGTGATTCGTGTCTAACCAACATACACGTGATGAACGCTGCCACTAGATCAAAGCCCAGGGGCAATGTTGCTAACCTACTGTTTATGATAACACGTGGATATGCTTCTGTCAAGATTGTGATTCCACACCATTAATAACGAAACAACATTTATAAGTAACAAATCCCTCTCGGAAGGTCTAAAGTAGTTCATAGAGAATGGTTGACCTTATTTTCATATGATATGTCCAAAAGTATATGTGCATTGACATCAAACACGAAATATCAAAACAATAAGATCCATAAATCTATAGCATATCATAACAGCCTAAAGATATTGGAAAATGACAAAACTCAGCAGATTCAAACTGAATTGCTGTATGTCATTGGCATATCGCCGTAAAGTATCGAATCCTTGTCTTACCATTCACTGCAGCAGGCAATGTGTAGTCCACTACAATTAGATTAGGGTGTTCCTCAAAGACAGAGGACAAGAGGTTTTCTCTATCCGAAGGACCATGCACAATTATCTCTTTTCCACCCGCCTCCACAGTTTTTCCTTCATCTTGTGGACCACCAAAACATATAGGCACAGGTTGAAGTCCCGCAGCGATGGATGCTTCAAGAACTGCCCTCCCCATTTTGCCAGTACAACCATTCACCTTTACATCAAAATAAAACAAACCTAACTCAAACAGTATTACCTTGATAAAACTAGAAATCAATTAACAACAGTGTAATTTAAAATCATTGGCAAACATATTAATTAAAAAAAAGGAAGGAACCTACCATTATAGGGAGTGGCTCGATTTTTGATTTAACTAAAACCTCAACATTTTGTGCTGGAGCAGAACACATCTTCACCGAATATTTTCGACCTCGAACAAAACTATTGCAGCCATTTTTGCTGCTTGAACTGTAACGCATTCCTCCTCCATTAATGTAAAGTGGAACTTTGATGATAGCCCACATTGTTTTACTGCAATACAATGAGAAAGCGCACACAAATTATAAAGATTTTGACTCGTCAGCAAAGACACTCTAGGGTTTTAAAAATTATCGGCCAAGTGTAGGTGTTATTTTAGTTTTTAGGACATTGCTATAAAATTTGTATAAATGTTGTACTTCATTAAGTTTAATTTTACAAAATGAATAGTATTATGGAAATTGTAAAATATTTTGTGTAAATCAAGTTCTGTAATAAACGATAGTAATAGAATTATTGGAGAAAATTAAATTGATTTTTTTAAAATAATAATATAACAGTCCTTTTGATACTTTAATTTTAGAGATTATAAAAATAAGTACCTTGATTTTATAATCTCAAAGACTTGCTCTTTGATTTATGAATCAATAAACGTAATAAAGATAAAATAATTAGTTTAAAGAAAAGGAAGATGTAAAAGAGGATCTCGAATCATTGTTCTTTAAAATAAAATTAAAATTCATTTTTAAAAAAATGAGTGTGTGTATATATATATATATGGAAAGTGAGAATAAGTATAAAAAGGGAGAGATAAATGCTTTAATTAATTAACAAAAAAAAGGGAAATTTATCTAATTTGCTTATCCACTCTGAAAAATAAACCAAATCCAATTTTTTTTTAAAAAAAAAACAACATAAAATTAGTAGTAGTAACTTAGATTCGTGGGCCTTAATATATTGGGGCCTAAACCCAAGGTTTTAACAGCGTCGTGGTCTGGTTGGCCCGCTATACTTTATAACGGTATATTTTATTTTATTTTTATATGAGATTTTTTTTATAAAAAAAATATATGAGATTTAAAATACTAATTAACAAAAAATATATATAGATAATGTCTCCTTTTAATGAAGATTATCAAAATGCTACATTATATTAATAGTATTCAATATAATTTTTAAAAACAAGGGCTCTAATCATTGATTAGAGCCCTTGTTTTTAAAACTTGTGTTTATTTTGATTCGTGATTAATTCATACAATAGCGATCAAAAACAAATAGGTATTCAAAACAAGTAAATGTTTAATTCAGTTATATTTTTAATTATGCTATTTTTATTCATGAAGTTTGACCTTCTCTAATTTATCTGTTTTCTTTATTATCTTTGCATTTTCTTTGAAGACTTAAATTCAAATTTAATTTGTCTGAAGATCAGAAAAAATTATATCAAATACAGTATTTGAAATGCTGAATTTCATACGAAACAACCAACAAACGGAAACTATTCAACCCTACTCATAATCAACAACCCAAACGACCCCAAAATTGTTGTTTGATATATAGTGATCTTCTTAACATTTTGCAAACAAAAATATGGAATAAATATAAATAAATATGCGACTAATAATTGTAACAAGATTTAATCCCTAATTTATATGGTCTTTCACCATTGGATGTGGTCATTATGATCAAGCACTATTACAAGCTGGGAAAGGGAAAGGGACAATCGATGGATATTTAGTGAAAATATTACTATAAAAAAATATTTTGATATGACAAAAGAAACAACTGGCTAGCCTTATAGTATAATAAAGAAAGTAGATGATTTGACCCTTTCGTCATTTGTTAGCTAGCCCCACAAAGTTACGTAGATATTAATAATGTAGTAATGATAAAAGAAATAACTAGCTAGCCTTATAGTATCATAATAAAAATAGATGATTTGATCTTTCGTCATTCGGTAGTTAATCATATAAATTATGCATATATTAGTAATGTATATTAAGTATTAGTTATATAGGGTTTAATTATGTAAGATTTATTTATACATACATATTATATAGATATTAGTTATCTAGAGTTTAATTATATGAAATTATTATACATTATTGAGCAATTTTTATTTCTTAATTCCAAAAAAAATTAGCAAGCTAATATTTATTATGACCATTAAAATAATCAACTATATGAATAACACGTTAAAAAAAAGGCAATTTTCACATATAGCAAACAAAAAAATCATATTTGTATAATATAACAAACTTTGCATAATTGCGCTCCATAGCAAACATAAAAACTGTATAATTCGCTATACATATAAAAGTGTATAATTCGCTGGCCTAAATTGTATAATTCGCTGGCCTATTTCGCTGCAATTGTATAATTAGCTTTGCATACAGTTAAATCGAATTAAAATGTATGTATATTGCATAATTATAAGTGTATAGCAAGAAGATATATGTTTCACAATATATACACTTCTGTTGTATAAAGCTAGAAAAAATTGTATTTCACTGCAATTGTATAATTCTTTGCCCTTTTTCTCTGCAATATTTGAAGTAAAATGTTTGTAAATTATATAATTAAGTGTATAACACGAAGATATACATTTTTGCATGTGGATATACAATTTTCTCTCGCTTTATACAAAAACAGAAACAGAAATTATACACTTCTGTGTATAAAGCGAGAGAGGCGAGAATGGGAGAGTGGCGAGCGAGACTTCTGGGAGAGAGACGCCTGACAAATTTTTGCCAACGTTTGCTATGGGGCACAATTAAATCAAACCCTAACTATTCCATTTAATTTAGGTTATTAGTTTGCTATTTTATACAATTTTCCCTTAAAAAAATACCAATAGCTAAAAATAATAAAAATAAAATGATAAAGGTAATTAAGTAATTGGTATTCAATATGATTAAGTATTTAAGTAATAGGTTATCTAAGTTATTTTTTTAAAAAAAAAAAAGAAACTAAAAAAATTGAAATGTGTATTGATGGTGCGTTGTAAAAAAACTATGCAGAGAAATTAATTACAAATAATAATCACTGCCTTGCCACCAAAGATAACAATTAACAAGAATTTAAGGAGATAAAATTATCAAGCAGAAAAACTCACAGTGCTTTTTTTTTTTACATTCATTAAATATTTGATTTGTTGTAGATTAATACATATTTAAAAGTAAATCGATAGTGATAGATATATTATTTACTATTTTAACACATATATAATTAATACACCATAACTTATTCCATTTTTCATCCCGCATAAGTTATATATAAATTTTTCATAAGTTATACAAATATTAAATATTGATTATGTAGAATTACAAAAAGCGCAAATAAACACCGTATGAAATTAATACATGAATAATTTTTATATAATATTTAAAAAACTACCAACCAAACATTGTATAAAATTAATACATAAATAACTTTTATATAATATTTGAAAACTTACCAACCAAACGTTAAAATTAATTCATGAATAACTTTTGATTCTAGTTGCAAAACAAACATTATATAAAAATAACACATAAATAACAAAGTAATTCATATATTAGATGTAGCTTAAGCCTGCTATCAAATGGCCCTTAATGTCTCCTTAAAATAAAGATATGAACCTTAAAATGGCCTCATTTTATATATATATATATATATATATATGTTGGAACCAATAAATTTGTGGTGGAGTGATATATTTTTCTTTTATGTGTAATTAAAGATCTCAAGTTTGAGTTCAAGTTTCTCGATGTATGAAGTTGTAGTTACTTGTTTCAATTTTTGCCATTAAATCAATGACTCACCTTGTTAGAAATTTTCAATAAATATCTTTTATATATTTGATGAAATTTTCAATATAAATATAAAATCTACAGAAAAGACATTAATTTTCCAAAAATTCATAACCACTTGGATCCGCCCTTGCCTCATAGTTTTGCTAAGTTATTATAATAGTATTATTAACTTATATCTATACTTAATAAAAATTCATAAAATTCAAACTCCAAATCCATCCTAAAATCATATGCTAAATGGAGATCATAGAGGAGGAAACTTGGTGAGGAGACTAATAATTGGGAAGAAGGCAAGATTGTTTTAGAATGAATGAATGAAGTAAAAAGGATTAGGTGTTTAGTTTTTGTCCACAACATGAAAGACCAATATGAAGTACAGTGAGGTGCAATGCCACAAAAAAAATAAATTGAGTACTCACTTATTTCCAGCTAGGAGCTTTAATGCCATTTGTACTTATATTCCATCAATCATTTCTACCTACTTCAACACTTATAATGAAACTTGCAACCTGTTTTCTTGTCTACAGGATGTTTCTTTGGCTTTTCCAATTCATTTATATGACATTTTGTTACAAAAAACTACACAAGAATCATATGCACAGAGTAAAAGAACTAGGTCATTTGCAAACAACTGAAAAGATAATTGTGAAGCAAGTTTCAAGTGAATTTACTATGAGATAAACACATGAATTGATAAATACAAATTCATCATGGCAATATTGTTATGCTTGCACAAAAGGAAAGAACTTTGAAATTAGGGAAAATTGTGTGAAAAATCGAAGTAAATAAAGGAGTCTTAGTCTTATTAAATTCGTTGACCAAATTTCAATGAGAGTTGGAGCAAGTCAACAGAATCACAATCATCACTACTCCTATATAAAACATCATTTTGATGCTACTAAACACATGTTATTTCACACAAACAAGGACTTGATCTTGGCAATAACAATTATTGTGAGAGATAGAGCATAATAATTCAAACTCATCAACAAGTTGTTGAGGTTGTTGAATCCATCCCATTTTTTAGTCAACTTTAACTATTCAATTGGCATAAACAATTGTTAAAAAGGAATAGTAATTGGAGTTGAAAAATAAAATATTTGGTAGTTGACAAATTGGTAAGGTTTGCAACAATTCTTGACTTTGTTATATTTACATATGTCATTAGTGACATAAGCATGGTTTTATTCTTCTATAAATAGAGCATTCTTGCTTATTTGTAGAACACACCAAGTTAGAGAGAAAAATCATTTTGAAAACAAAGTGAGGTATTCCATAGATTATACAAATAAATAGTCTATGAAAAAAAATAAAATGTGAGCGATATTTTAGTAAGATGGAAATCAAAAGAGTACTGAACTTTTTGAGAGTCTTACCTTCTAGAATAGGAGGAATCATGTTGTCTTGAAAAAGGTTGATGCAATTGGTTAAATTGTAAAGGTAGTCTCTTTGATTATAAAGTTAATGACTTCAATTTAAGTGAATTTTAGAAGTAAATCTTAAGAAAGTAAGTTGTAATTTATACTTTCATAAGTAAAGTATTTTTTTTATCATAAAGTTTCTTGTGTTATTTATATTTCATAATTTATTTATTATTTTGTGACGGAAGAGTCCAATTTTTTTTTTTAGCCCACTGAGTGGTTGCTAAAATAACAATATGTTTCCCAACTTGTTGATTAATTTTATTTTCATTTCTAGAGTTGTTAATGTGAACTATCATATTTTATCCAAGTTAGTTTATACATGTTTTAAAATATGATGATTATGTCAAGCTGGTTCATGTTTTTGTATGTCAgAAAATAGTCGGTCTATCTCATCAGTATGTGGATTGTGGGCTATGTCATTTTTTTAAAAAAACATATATTTTTTAATTAAATTATTAACGTAAATATCGATAAGATATTTTCATTTTAATCTATATTTTATATGCAAATTCAATTATTTAAACATTAAAAATACCTAAATTTAAGAACTACATATAATAATTAAATGTACACAACAAATATTAATAAGTTTGAGAAAAATCATATTACCGGTTCCGAGTACCTACAAATATTTGATTATTTTCATGATGAATGCATGGTAAGAACACATCTACCAATGCTGAATGATGATTCTGATGATACAATAGTAATAGAAATAGGTACATCACGTTTGATGATTTTAAATTTCAATATTTCAAACTTTTAATAGCGGTTGAGTTTGAACTTTTGCAATTTTTAATAGTTTATTGTATTTTTAATTTTAATCATCTCTTTATTTAGCTCACAGACCAACTCAACTCATATTACTTAACGCACACACACCAACATAATTATTTGGGTTGAATTAAAAAGTTTCTTTTTTTAAATGAATTCCCAAATTTTAGATCAATCCTATTGAATCACGAGTTAGATCAGATCAATCCAACATATCTTTCCTTTCATATTTATACTGACATATTTCCCCTCCACTTTAACTTAATTATAAAATTAAATTTTGTTAAATGACATAATCATAAAAGACCTATATCGGTCGGCCCCATACGAAGACATATTTAATGCCACACGCATACCCACCCCTCCGTTTCTTGTTTGCGGAAAGGCAAATATTTTTGGTAAAAAACGTAACTAATTTTCTCCCTTTCTTTATCCAATTTAGGCCACACCCACATTTAAGAAAACTCCATGGGTATATGTACTTCATTTTTCTCAAAAGGGATACGTGTACAATAACATACCAAATAATGCATAAAATGTATTGATAAAAATAGTATTTTAAGCCAAAATAACATAAACGTAACAAAAATAAGAAAATAACAAGATGATACACACGTGTCGCATCCGTAGGTCCGTTTGTCGTCACGTGCCTTATGGCGTTCCCCCCACGCTTTTGTCCCTCAAAGGACGCCGTTCACTACTTTTTGTCTTTTTTTTCCAACAATTTTGGCAATAAATCAACACTCTCGGGGCCATCTCATGAATACCCCATACTTTAAGTCCTCATCCCCGAAACCTAGCAATTTGCTAAATATTACTATCTGGGTCCTACATTCCTTACCCAAAACTTTATTACTCCTTTAACCCCTCAATTTGTCGTTTATCTACTCTACCACCCTGTGTGTACTTTAGTGTTCGCCCCTTTCTCATTGCTATTCTGCGCCTCGAATCCTTATCCTTCACCTCCCCCTTCATTTTCTTGTTGGTTCATACCCATTTATTTCTCTCCTTGTTGTTAATGAGCTGAAGGAACAGTGTGTCGAGAGGGAGATGAAAGTACTACTGACAAAGAAAGAAGCTTTATTGTTGTTGGGGTTTTTCATGGAAATGTTCTTTCTTGGTTTTGTTTAATCGCTTGTGCTTTTTTTTTTTTTTTGCTTTTCACCAAATCAAGAAAAAGGAGAAGCGGCTGTCT**ATG**GCAAGAAATAATGAAGCTGTTGCGAATGAATCCAAGAGTGAATCTAAGGTACTCCGCTTTTTTCCTGTTTTCTTTCTCTGATCATCAATTTGATTTCTGTTGATTTATGGGTTGGCTTCAAGATTTTGGGTTTTACAGTATAGCTATGGTTTGGAGTGATTTTGTCATGCAGATTTTGAGGGGTAGGGTTGTGTTTTACTCTGAATTTTGAAATTTGGTACTTTTGAAAGGATGTTTTTTTTTGCTTCTATTTTTGGTCTATCAGTGCCCTGTACCCCACAAATTTTACTTGTTTTTACAGGAAAAATGAGTGCCTATTTGTGTAGATTTTGGATATGTATACCTTGGTGGTGTGGGTCTGGAGTTGCTCTAATTGTTCCCCTAAAATTTGAAAACTTGCCCTTTTGTATGATGGGTCTGAATTATGTTTAGCTATTCTTCTTTTAGTATGCTGCACTGTCTTGTGTTTCATGGCATATGTATAATAGTTTGGCTGATAGCAGCACGTTCTAGTGTTATTTACAGTAGTTCTGCTGTGTGTGTTAGATGTAATTATGTAAATTAAGGTTATCGTTATGCATATATCATGGCTTTTATCACTTCAAATTGTACAATTTCTGAAATTTTACCTCTGTTTTCTTATAGAGAGTGGTTCCGCTCAATACATGGATCCTAATTTCAAACTTCAAGTTGTCTTACAATCTTCTCCGTCGCCCTGATGGGACTTTCAATCGTCACTTGGCAGAGTTCCTTGACCGCAAGGTTCCAGCGAATGCAAATCCAGTTGATGGAGTTTTCTCTTTTGATGTTCTCATTGATCGTGAAATAGGCCTACTTAGCTGTGTCTATCGGCCTTCTTTTGAGGATGGAGCTTCACCGAATATGGCTGAACTTGAAAAGCCTGTGACTGCTGATGTTGTACCTGTCATAATTTTCTTCCATGGTGGAAGTTTTGCACACTCTTCTTTCAATAGTGCCATCTATGACACACTTTGTCGCCGCCTTGTTGGCATTTGCAAGGCAGTTGTTGTGTCAGTTAATTACAGGCGAGCTCCTGAAAACCGTTATCCTTGTGCTTATAATGATGGATGGACAGTTCTTGAGTGGGTTAACTCAAGGGAATGGCTGCGGAGCAAAAAGGACTCGAAGGCTCACATATACTTAGCTGGAGATAGCTCTGGTGGTAATATTGTTCATAATGTGGCTTTCAGGGCAGTAGAATCCAACATAGAAGTGTTGGGAAATATACTGCTGAACCCTATGTTTGGTGGACAAGAGAGAACAGAATCAGAGAAGCGATTGGATGGCAAATATTTTGTCACACTTCAAGACCGAGACTGGTATTGGAGAGCTTATCTTCCTGAAGATTCAGACAGGGACCATCCTGCATGCAACCCTTTTGGTCCAAATGGTATAAACCTCAAAGGCGTCAAGTTCCCAAAGAATCTTGTTGTTGTCGCAGGTTTGGACCTTGTTCAGGATTGGCAGTTGGCTTATGCTGATGGGCTTAAGAAGGCTGGACAAGAGGTTAACCTGATATATTTGGAGAAGGCAACAATAGGGTTCTACCTGTTGCCAAATAATGAACACTTCTACACTGTCATGGATGAGATAAGTAGCTTCGTGAGTTCTGACTCTCAG**TAG**ATTTAACCTTGTCAAAAGTAGGATATGCTTTGAAGACGTTTGATGTTTTGTTGAAGTTAGTTCCTAGCCTGTCAACCGTTTGAAAGATTGTATAGCATCATCAATTACTTCCTTATTGATCATGCTATTGCTTGGATTCTGCTCCATTGGCTGGGTTGGTTATTGGTGGCGGAAAACCTCAATCATGTAGCTGGATCTGTGTTATATTTATTCCAGGTACAGGGAGTGTCTGGATATTGGTTGTATATTTTGGTAGCTTGCGCCAAAGGTTATAATCATCTTGTCTTCGTCTAGCAGATATGCTGATTTACAGCTAACCTTTCCCATGTTAGCCTCCGTAGTAGAGGGGGTAAGTCTGGTGCTGATCCGTCAGGGGACCAGTGCCTTGCTAATGTTATATAGCTGATCATACTATTATGCTTCACGGATGAACATCCTAAATTGTGAAGTTGTATAATATATCTATAATTATATAGAACTATGTTTGCTTCCTCGCGTTATCATGGTGTGTCGCCCTTTGGTTTGTTTTCTTTTTTATGTTCTTTGTCACTTCCTTGACCTTCAATATGTACATCCTTAGATCACTTATATTTAGAAGATATGTTTGCAAGTACTGTTTCTTTTCTCAGGGATGCACAATATGAACATATGTAATTCAACATGCTTCAATTGTTATAAGTGGAATTGTTGTTCTCTTGCACATGAACTGATGTGCTATGAGGTCTTTCTATATTGCAATGTTGGCACTGTCTAATACATGAGGTGACTTTTTTGTTGGTCAACTGGAAAGTGTTACTTATTATGATTAGTGGAGTGTAGTTCTGAAAAAATTATAGTATTTGAATGTAAGTAGCGCGAAGGTGGAACCTATTCATCAATTAAATGAGTGAAAATCATACTAAACTATATGTTTGGCCACAGATTTTGCCTCAACCAAAAAGATCTCAAAGAAGTGTTTGTCTATCAAATTAAACCATTATTTCAAAATATCGTTGGACAGGTTCAAGTCGAGGTTTGTGTAAGCTGATATGTCCTCAACAGAAAAAAGAAAAAGAAAAAAAAGAAGCTTAAATACATGTGTAGAAGCAGTGGTGATTTGAGAATAGAGACAAAATAAGCTGTCTGTCTTTTGGTGACCTTTATGAATCTAGTCCTATAGTGTCAAACTAGTGAAGAACTCACTGGCAAAACTGCTTCTTTGCTCCCTTCCCCTTTCTATTTAGTTTAAATTTAAATAAATTATATATGGCTCAAGATAGTGGCGTTTTCTTTTATATAATTTGAGTAAAACAAAAAGATTTTGCAAATATGTCATGTTTAAAAAATGCTTAAGAGGGATTTAACAAAAAAAAAACAAAAACGAATTGACTGGTCACAGACATAGATTTTGGAAATATGGATACAGATATTGACTTGTTAGGAGAAAGAGTCCAGTTGGCGGTGATTGCTATTTGGATGTATTGAAGGGGACCATTTTTCAAAACCAACAAAATCAATCAGGTGAACTTTTGTTTTTTCTTTTATTTTCTTAGAGACCAAAGTGGGTGAGCTCACTATTTCTTTAAGAAAAAAAAAAACGTGAATCTGTACGCGAATCTTACTCTTATCTTAGTGAAGCTAGAGAGGTGATTAAGAAAAATCATGCATCAAAACTCGTATTTGTGTCTAGTCAAACAATGTCATAACACATAATACTCATATTTGTTTTGTCTATTTTTGTGGGTGTCATTTCATGAGGTAAATGTAAATGAATGAAAATTACAGTTTGTAAGAGTGGAAGTCATTGTCATATGGAAGAGAAAAGGTGTCTAATCATGGATAAGGGAATTCGAGTGCTTTTGCGTTAAAATAAAGGGAATAAATAATATTCTGACTATAGAAAAATGTTTGAAATTAGAAATTGAAAAACAGAATAATATTACTATAAAAATAAAAAAAGAACAATATTTGCTACTTCATGAATCATGAACAATAATATATATAACATATAGTATCAAGTTCATTTGATTGAATTGTACAATTTGCCCTTCAAATTGGTTGATGTTTAATTCTTGCCCTTAGCAATCGAGACATAAGTTCTTTAAAAGCGCGCAATAGAACAAGTGGGGAATGACTTTACAAAATCGTGACTATTTTTCAATTATAGACACGTATCGTATTTTTACCCTTTTTCTGGAATGTGGAGTATTTGGGCTGTGAAGATGTGGCCCAAGTATCCTCCACTACATGGTCCTAACTAGATCTAGGCCCAATTCAAAACGCATGAATAAAGGAGAAAATTGTGTATATATAATCAATGAAAAATAAATCAATTTATAATGAATTTTCATAATACATATTAATATACTCATATATCACATGTTATACCAACATGCATGCAGGTATGACGAAAATGACAAAAGGGTTCCATCAATATTCACACTTATATGTATAAAAAATAAATAAATAAGATAATTTCACTAACATCAATGTTCAGATGTATACAAAATAAAAAAAAAGGCCAACAAAGTCATCATTGTTTTGTTATTATTGTTGTTGTTATCGTTCTCCCAGAAAATTATACTAATAGTATAATAGGTAATGGATCACATGCAATTAATATTAGAAAATATATTGTTTGGCTGTACTTTGCACACACACCTAATAGTAGAGTAAGAAATCATCAATAGCCAAATCATAACTTAGTTGACCTTCAATTTTGAAACCACATATGTTCAATGAACCCACAAATAAATAAATAAATAAATATCAACTTTATTTATTTACTTTTAAATAATTTGGAGTAGCAAAATTCAAAGATAAAGAAAAAGAAAGTAGTGTTTCCATGAGTACAAGTGGTCCTAAGAAAAAGACAACATAACTCATAATCGTAAAGTAGAAGAATTAAATTTTATAGTAATGCCAAGGAGCAAGGACGGTGCATCAAATCGAGAATATATCTAATTTATAGCTTGATTTCTCACATTTTAACTGTAATTTTAGCTTAAAAAACTTATTTATAAGTTGATAGTTGTTTAATTATATGCTTAGCACTACAGATTTACAGACAATATTTAAAGAAGATAAAATAATCTCTCTTTAGACTTTAATAAATAAGATTACTGAAGACATATACTATATCTTATATTTGTCTTCGAACTTGGTTAAGGATATGGTGCACACAATTTTATATTTTGTGTTTAACTATTTTTTTAGAGATGCGGTTTGTTTCTTGATAGAATAATTCGAAAGATTATTATACATCTATTTTAATTTGACATCTCTAACATTTGAAACTATTCAAAAATTTTAGTGACATCTTAATTCGATATTAAATGTACATTGATCATGCATATAATCAAGCATTATTAAGTAGAGATTTAACTTATTTACAATAATTCTAACAAAATTAAAGATGATAGCTACACATAAACTACATTAATGAAGTCCTTCTCATCTTCACTTTCATTGCTTATACATTTGAAGCCAATATCATGAACACAATCTAAATTAACATATCCAAATAACTTCTAATTAAAGTTGCCATTAATTAAACATAATTAAATTCCATATATCTCATCAATCAATATTCACACCACTGGAAATGATCCTCCGGCATCCACGAATTGTTTCTCCAGCTACAAATTAATCAATTAATTGCAAAAATGAGTCCAAAACACCTCTTCCTCACTTGTTATACAATTAATTAATCATATATAGATGAAAAATGAAAATCACCTTAAATAAAGTTTCAAGCTTGTTCTTGACAAGGGTGTACTCAATTTTCACTTGTTGCAAACATTTTAAGCACCTGCATTCAAATGATACTATTTAGCTTTATTAATTTCAGTAACAACGTATCTAATACAATTTCATAAATTGAGTCCGCAGAGTGCGATATAAACAGATTTTACTTTTAGATTATATAAAAAATTGAAACGAAAACACCAAAAAAAAAAATTAATTTAAAACCTACCCTTCAACATTGCGTTCATCACAATAATTACGGAAGGAAATGCACACTCTTTGAACTCTTTGTGCCCCAATGCTGTAAATTAAAAATAAGAGTTTAAATTATGTACCCTCAACTAATTAGTTAATATATAAAAAAATTATATTATCAAATTTTATCTAATAGATAATTAGAGATACCGTGAGTTAAAATAGGTTTAATAATAGTAAAAAGAAAAAAAGAAAATCTTAATATACAAAAAAAAATATTTACCTAGAGCTGCTACCTTTCAGCTGATGAACATGAGCATCCAGTTTCTTGAAGTCTACATTAGACTGATTGCTATTTTTACAAACAAAAATTGAAATATTATGTGAGCTTGCTTATATTGTCGATCTTAAATACAAATAAATGCGAAAGCTTACATCTAGCGTAAAACTTGTCAGTTCATAATAAAACAGAGATAATAAGTTCATAAATATGTTTTTCCACCTACATCTCTTCCAGATTTGTATGTAAAAAGGGGAAATTAAACAAATTAATTACTCACAGAGCTTTTGCAAGTTCATTAAGAAGCCTTTCAGAATCTTCAAAGAAAAGGGATACAACTTCCACCACAAAATCAGGATTGCTCTCATCTTGCAGTTGCTGAAGTTGTATAAACTGTTCATCCAGAATTTTCTTCTCACACCCAAAAAATTAAAAAAAAAAAATCAACAAATCATAATAAAACAAAAAATTCAATGCACAGAACATTTCGCACATTGTTTTTTGAAAAGGGAAGTAGTTAAAACAATTTATTTTACTGTTCGAACTCGTAACCTATAGTTCACTCAATCATATGATAAAAAGTTTTATTTACAAATTTAGAGGTTAAGATTATTTTACCTCATTGTACAAGGAAGCAGTGTACTCAGCTAGTTTTCTTTGGATTTGTTCCATGATTTTGCTAAATTCAAGAGTGACTTTCACAAATTAACAGCTTGCTATGCTATTAATGAAAATTGAGAAGTTATATATACTCCTTATTTGTGCATGGAGTAAATTTTTTTTTCCTTGGAAAAGCTACAGCAGGGGCACTCTTTTCTATAACAGAGAAGACTAATATTCAATGAATTTTGCGCATTGATTCACTGTATTTATATTGTCACACTTTATTTTAGTAATTTTTTTTCACCCCCTCACTTTCTGCAATTGTGCTTTTGTTCCTTGTGCATCCTAAATGTCTCCCCTTATTGTTGTTTTTTTGGCAGTTATCTATTTCATTAAAAATATAGTAAAAGCTGTCACAAGATCCAAAATATTTTTTACCATACCCTATTTATTTGTTTCTTCTTTAGTCCTTTTTGTTTTATACTAATTCAATCTCGTATTTCACAGCTTAACTTATTTGAAGTTGAAATTAAAAAAAAAATCGAGAGTACGTCAAGTGAAACATAGTGAGATAATTTAATATTATTAAGTGAAAATCTACAATCAAATACAAATTTCAGATTTTTTATTCTTTTTTCTATTTCAATTTTAAGAGGGTTGATCCTGTTTGTTATTAATTTCTTTATAATTTTAATTAACTATCGAACAACTATCGAACAACTTCTATGTATTTATTTATGTGTATGTTTAGTATTGCTCGATTTGAGACTAATGAGAACGTCTTATATGTACCATCCAAAATTATGGTGAAATAGACAGAATCCCATTTATAACTATCTTTTTTGATGAATCTGAACAATTTGTAAAACATGTACATAGTAAACTATGGATGAATAAATTATTGAAATATGATAAAAGGAAAACTTATTTATTACATATGAAAGTGTAGCAGAATCCAATGTTGCACTGCAAATTGGAATATGTTTAGTTTTTAAGATGGAAAGGTTGCAAGTATGGGCAGGTCAGTATAAGGGGTTGGGGACAGAGAGGTGAAGCTGACCACCCATGTTACATCAATATCTCCAAGATTTAGTTGGTAGATTCTCTCTGCATATTCTCAACTACTATCAATTCTTCGATATTTGAAATTTACTGATTTGATTAATAAAAATATATTATATTAAATTTATTTACATTCAAATAAAAAATTTATGATTTATGATCCTAATCTGCGTCATTTGAAATAATATACTATATTGTGATGATATTGAAAGTTTAAAGTAGCGTGAAATAGCGTACTGTGACAACAAATGTGTCGTGTTTGACATGTGAAAGTGAAAGCCAAATGAAACCATCTTATCTTGGGATCAACTTGTTTCTAGCTAGGTAAAAGTTAAACAACAATCTTATCTAAATCCAAATGAAATATCCCTTACCTACTCTTTTCTTTTTTGTTCTTTTTTAATTATTAATATTCCCTCCGTCCAATATTATTTATCATGTTACGCTTTTTAAAAGTTAATTTGATTAATTTTCACGTTAAATTAGATTGCATTAATTCGATATTTTAGATAAAAAAAAATAGACATTATATGAAAAATACTATAAATTACAAATTTTTTACATGTTAATATGATAAAAAAATACATCTTAAAATATTAATTAAAGTTTTTATAATTTCACTCTAAAAATGAAAATCATGACAAACAATATCAGACGGATAGAATACTAAATAGAGGTGGTGATATAACTATATTAACCTTTGATATATTTCCTGGGCGCAATTTTACTTATTCAATATTGATCCAGCACATTCTCTGAGGAGTAACAAATAGTAGAATGGCAATTGTATTAACTAATGTTTGAGTAGAAAAAGATCAATATATTAAGAAAATAGTATAATTAAGGGATAATGCATAAGTATCCCCTCGACCTATACCCGAAATCTCAGAGACACACTTATATAATACTAACGTCCTATTACCCTCCTGAACTTATTTTATTAATATTTTTCTACCCCTTTTCGACTTACATGGCACTATCTTGTGGGTCCAATGCTAGTTGACTTTTTTTTCAAGTTAGTGCCACGTAGGACGAAAAGGGTAGAAAATTACTTCTAAAATAAGTTCAGGGGGAGAATAAGACCTTAGTATAGTATAAGTGTGTCTCTGAAATTTCGAACACAGATTGAGAGGGTAATTGTGCATTTTCCTATAATTAATAATAAGAATAAATTAAAAACAAAGTAATAAATTTAATTATCACTAGATTTTTTGAACTGGATAAGTAAAAGACAAATATTTATTTTTGGAATATAAAAGATTGGGAGTAGTACATTCGTATTTTTGGAATGATAATGGTAAATAATAAAGATAAATTGAAAAGTAAATGATGATTAAACTATTTCTTATTTTTAATTAAATAATAATAAATTATAATATATCAACAACTATATAAGGATTAAAAGGGAATCTGCTTCTGGTCTTTTTGTTTTGTTATGTTGTTGATATTTGGTTGGTTGGATCATTGTTTACTAACTTATTGTTATTAAAATTATCTTCGAAAGGGATGATTATAATTGGTCCCATATAACTATATGATTGTTGCCTCCACAACAATAAGAAGTTAAAATAATTACCTCTCTTTCTAATATAGTAACCCTCCCCACATAT

**Alignment of the DNA sequence of the *qtph1.1* locus in SG-7 and TS-165**

The exon and intron of *SlGID1a* gene were highlighted in purple and grey color respectively, the SNPs of *qtph1.1* locus between SG-7 and TS-165 were highlighted in yellow color

SG-7 1 GTGATATAAGCATGAGGTTTGTCCACTGTATCTCAAATTAGAAAATAGTAAAAGAAACAA 60

||||||||||||||||||||||||||||||||||||||||||||||||||||||||||||

TS-165 31 GTGATATAAGCATGAGGTTTGTCCACTGTATCTCAAATTAGAAAATAGTAAAAGAAACAA 90

W1J13

SG-7 61 TCCTAAGAAGATGGTGAATTTGCTATGTTTAATCCAGGAGCATGCAAATAGCCAAACCAT 120

||||||||||| ||||||||||||||||||||||||||||||||||||||||||||||||

TS-165 91 TCCTAAGAAGACGGTGAATTTGCTATGTTTAATCCAGGAGCATGCAAATAGCCAAACCAT 150

SG-7 121 AACCTAAAACACAGGCACGATACCTTGTGGACACTAAATGATCTAGTAGGAGGCATTTGG 180

||||||||||||||||||||||||||||||||||||||||||||||||||||||||||||

TS-165 151 AACCTAAAACACAGGCACGATACCTTGTGGACACTAAATGATCTAGTAGGAGGCATTTGG 210

SG-7 181 ACAATATCTGGTTGAAGTTGAACAAAAAAGAATTTGAAGATGAAGTTGAAAAGGAGTATT 240

||||||||||||||||||||||||||||||||||||||||||||||||||||||||||||

TS-165 211 ACAATATCTGGTTGAAGTTGAACAAAAAAGAATTTGAAGATGAAGTTGAAAAGGAGTATT 270

SG-7 241 TTGAAGTTCAGACATTTGCAAGTCATTTAAGACCCCTTAATGCTCTGCCTATGTCTCTCC 300

||||||||||||||||||||||||||||||||||||||||||||||||||||||||||||

TS-165 271 TTGAAGTTCAGACATTTGCAAGTCATTTAAGACCCCTTAATGCTCTGCCTATGTCTCTCC 330

SG-7 301 ATCCATGAACTGCAAGAGGCAAACTGAAACCCCAGTTGTAAAAGGAACAGCTGCTTAAGA 360

||||||||||||||||||||||||||||||||||||||||||||||||||||||||||||

TS-165 331 ATCCATGAACTGCAAGAGGCAAACTGAAACCCCAGTTGTAAAAGGAACAGCTGCTTAAGA 390

SG-7 361 GCTCATAATTGAGTGAGAAACAGCCATTTTATTCGCACAGCAGTCTAGATTTAAAAACAA 420

||||||||||||||||||||||||||||||||||||||||||||||||||||||||||||

TS-165 391 GCTCATAATTGAGTGAGAAACAGCCATTTTATTCGCACAGCAGTCTAGATTTAAAAACAA 450

SG-7 421 CTTCTAGTGGCCCACTGGATCAATCACACAAGCAAACAACTGGTGGTTGGTCAGTCCATT 480

||||||||||||||||||||||||||||||||||||||||||||||||||||||||||||

TS-165 451 CTTCTAGTGGCCCACTGGATCAATCACACAAGCAAACAACTGGTGGTTGGTCAGTCCATT 510

SG-7 481 TCTACTGTCCAATTTGCCCAATCACACAAAAAGTCCAATTCTGCTGTCCGCCaaaaaaaC 540

||||||||||||||||||||||||||||||||||||||||||||||||||||||||||||

TS-165 511 TCTACTGTCCAATTTGCCCAATCACACAAAAAGTCCAATTCTGCTGTCCGCCAAAAAAAC 570

SG-7 541 TGATTTTGTAGAGCACTTCAAAGAAACAATCAACGAGTTCCAGTTCTTCCTGGTAAAGTG 600

||||||||||||||||||||||||||||||||||||||||||||||||||||||||||||

TS-165 571 TGATTTTGTAGAGCACTTCAAAGAAACAATCAACGAGTTCCAGTTCTTCCTGGTAAAGTG 630

SG-7 601 TACTTCAGAAAGTAATTCCCAATAGCTTTAACATCTTCTACAGCAACATTAAATATCAAA 660

||||||||||||||||||||||||||||||||||||||||||||||||||||||||||||

TS-165 631 TACTTCAGAAAGTAATTCCCAATAGCTTTAACATCTTCTACAGCAACATTAAATATCAAA 690

SG-7 661 AGCCAATATGCAAATATCCGGATGAACAGAATTTATGTTAACCTAGTATACATGTCAAGG 720

||||||||||||||||||||||||||||||||||||||||||||||||||||||||||||

TS-165 691 AGCCAATATGCAAATATCCGGATGAACAGAATTTATGTTAACCTAGTATACATGTCAAGG 750

SG-7 721 AAAACATAAACTGACATATCAATGCAAGTAATGCAACTAAGAGCATTTTAAGGTAAAAGA 780

||||||||||||||||||||||||||||||||||||||||||||||||||||||||||||

TS-165 751 AAAACATAAACTGACATATCAATGCAAGTAATGCAACTAAGAGCATTTTAAGGTAAAAGA 810

SG-7 781 ATGGAAGCATGCACAGAATATAGTCAGCATTTCAAATCACTATGAGATACAAGAGTTTGG 840

||||||||||||||||||||||||||||||||||||||||||||||||||||||||||||

TS-165 811 ATGGAAGCATGCACAGAATATAGTCAGCATTTCAAATCACTATGAGATACAAGAGTTTGG 870

SG-7 841 AAACCAACGGTCAACTAGCATGGCAAAGGAGACAAACCTGTAAACTGTACCCAGAGAAAG 900

||||||||||||||||||||||||||||||||||||||||||||||||||||||||||||

TS-165 871 AAACCAACGGTCAACTAGCATGGCAAAGGAGACAAACCTGTAAACTGTACCCAGAGAAAG 930

SG-7 901 CTCCAGGGAATTGTTCTGCCATGATTTCCATGGCTGCGAGAAAAGCTACCACCTAAAATA 960

||||||||||||||||||||||||||||||||||||||||||||||||||||||||||||

TS-165 931 CTCCAGGGAATTGTTCTGCCATGATTTCCATGGCTGCGAGAAAAGCTACCACCTAAAATA 990

SG-7 961 GAGCCAATCATACAGGTATACCTCCTGTTAAACTACAGTCTGGCCCACAATAACAACAAC 1020

||||||||||||||||||||||||||||||||||||||||||||||||||||||||||||

TS-165 991 GAGCCAATCATACAGGTATACCTCCTGTTAAACTACAGTCTGGCCCACAATAACAACAAC 1050

SG-7 1021 AACATACCCAGTGACAAAAGAAGAGCAAAATGTCTAAACCTGCTTTCCCATTTGTGGGGA 1080

||||||||||||||||||||||||||||||||||||||||||||||||||||||||||||

TS-165 1051 AACATACCCAGTGACAAAAGAAGAGCAAAATGTCTAAACCTGCTTTCCCATTTGTGGGGA 1110

SG-7 1081 GATCACAGCATAAACTTTTGAGTCTACCACTGTTTTGTACAGCTTCTCCCTATCTCCACC 1140

||||||||||||||||||||||||||||||||||||||||||||||||||||||||||||

TS-165 1111 GATCACAGCATAAACTTTTGAGTCTACCACTGTTTTGTACAGCTTCTCCCTATCTCCACC 1170

SG-7 1141 AGTTGTTCCCATTACAAAAGGCACTCCAACTTTACAGTATAATTCAGCATTATCTGCAGG 1200

||||||||||||||||||||||||||||||||||||||||||||||||||||||||||||

TS-165 1171 AGTTGTTCCCATTACAAAAGGCACTCCAACTTTACAGTATAATTCAGCATTATCTGCAGG 1230

SG-7 1201 TTCATGGTGGAAACAAGAACATTACATGGCTATGTCAATGGAATCTCCAGCAAAGAATGC 1260

||||||||||||||||||||||||||||||||||||||||||||||||||||||||||||

TS-165 1231 TTCATGGTGGAAACAAGAACATTACATGGCTATGTCAATGGAATCTCCAGCAAAGAATGC 1290

SG-7 1261 AATATATATCATTTTGGTGCTTTCGTATTTCTATATCTCATTGCACACCCTCTACTGTCC 1320

||||||||||||||||||||||||||||||||||||||||||||||||||||||||||||

TS-165 1291 AATATATATCATTTTGGTGCTTTCGTATTTCTATATCTCATTGCACACCCTCTACTGTCC 1350

SG-7 1321 TCCACTAAAGACATGTGTAAAAAATTATCACAGAAGCCTACCTATGCTGGCTAAATTTTA 1380

||||||||||||||||||||||||||||||||||||||||||||||||||||||||||||

TS-165 1351 TCCACTAAAGACATGTGTAAAAAATTATCACAGAAGCCTACCTATGCTGGCTAAATTTTA 1410

SG-7 1381 TTTATAAGAACCTCAGAATCAAGATGATTGAATTTACAGACAAACTTAAAATTTTAGCAT 1440

||||||||||||||||||||||||||||||||||||||||||||||||||||||||||||

TS-165 1411 TTTATAAGAACCTCAGAATCAAGATGATTGAATTTACAGACAAACTTAAAATTTTAGCAT 1470

SG-7 1441 TTTACAGAAAAACATTATAAAACTTCACCGGACACCAAAAATTTTAAGTTGCAAGGAAAT 1500

||||||||||||||||||||||||||||||||||||||||||||||||||||||||||||

TS-165 1471 TTTACAGAAAAACATTATAAAACTTCACCGGACACCAAAAATTTTAAGTTGCAAGGAAAT 1530

SG-7 1501 CTACATTTTACAAGAGAAATAGTCTATGTTTTCCTCTCAACACAACTGTGGTTTCTTTCT 1560

||||||||||||||||||||||||||||||||||||||||||||||||||||||||||||

TS-165 1531 CTACATTTTACAAGAGAAATAGTCTATGTTTTCCTCTCAACACAACTGTGGTTTCTTTCT 1590

SG-7 1561 AACAAAATCATCCAAAATATTAATAAAGGAATGGTTCTCCATTTCTGCCCATAATACTTA 1620

||||||||||||||||||||||||||||||||||||||||||||||||||||||||||||

TS-165 1591 AACAAAATCATCCAAAATATTAATAAAGGAATGGTTCTCCATTTCTGCCCATAATACTTA 1650

SG-7 1621 GCTTCTGTTGGTTCCAGCTAGCCACTGCTTGCTCACAAACATCATTCACTGAATACCATA 1680

||||||||||||||||||||||||||||||||||||||||||||||||||||||||||||

TS-165 1651 GCTTCTGTTGGTTCCAGCTAGCCACTGCTTGCTCACAAACATCATTCACTGAATACCATA 1710

SG-7 1681 AAGCTTCCAAAACATACACACAATTTGCTAAGTTACAATGTCGCAAGATATATCATACAG 1740

||||||||||||||||||||||||||||||||||||||||||||||||||||||||||||

TS-165 1711 AAGCTTCCAAAACATACACACAATTTGCTAAGTTACAATGTCGCAAGATATATCATACAG 1770

SG-7 1741 ACCCAGCAGAAGCTTCACAAAAGAGTTGCGCAAGCTGCTTTATGTTATTAACCATGCCTC 1800

||||||||||||||||||||||||||||||||||||||||||||||||||||||||||||

TS-165 1771 ACCCAGCAGAAGCTTCACAAAAGAGTTGCGCAAGCTGCTTTATGTTATTAACCATGCCTC 1830

SG-7 1801 CTCCAAAAGCTAGCCATTAAAAACATCATCTCTGTTAGGGATTAGGCTCTCCAAAGAATT 1860

||||||||||||||||||||||||||||||||||||||||||||||||||||||||||||

TS-165 1831 CTCCAAAAGCTAGCCATTAAAAACATCATCTCTGTTAGGGATTAGGCTCTCCAAAGAATT 1890

SG-7 1861 TTCAGTATCCGCTGAGTAGATTGGCCAATTTCGAGTTTCTGCTCTTGTCCACATCATTCA 1920

||||||||||||||||||||||||||||||||||||||||||||||||||||||||||||

TS-165 1891 TTCAGTATCCGCTGAGTAGATTGGCCAATTTCGAGTTTCTGCTCTTGTCCACATCATTCA 1950

SG-7 1921 GCCCCCCTCGCCTATTCTTGTGAAGCCACAGTTCTTGTAATTTGTCTACTTCCATTCCCA 1980

||||||||||||||||||||||||||||||||||||||||||||||||||||||||||||

TS-165 1951 GCCCCCCTCGCCTATTCTTGTGAAGCCACAGTTCTTGTAATTTGTCTACTTCCATTCCCA 2010

SG-7 1981 AGACTAAATATCACTTTGAAAGGATATGTTAAGACTGCACTTGCTTCTTCTAATAAGCTC 2040

||||||||||||||||||||||||||||||||||||||||||||||||||||||||||||

TS-165 2011 AGACTAAATATCACTTTGAAAGGATATGTTAAGACTGCACTTGCTTCTTCTAATAAGCTC 2070

SG-7 2041 AACCAATGTTGCTAATCtttttttttCTCTCTTTTGATAACAGATAAATCCCGAGTCTAG 2100

||||||||||||||||||||||||||||||||||||||||||||||||||||||||||||

TS-165 2071 AACCAATGTTGCTAATCTTTTTTTTTCTCTCTTTTGATAACAGATAAATCCCGAGTCTAG 2130

SG-7 2101 TAGCACACGCTTTGAAACTTGGTGGATAGTGTACCCACTACCTTTCTCCACTTAAATATC 2160

||||||||||||||||||||||||||||||||||||||||||||||||||||||||||||

TS-165 2131 TAGCACACGCTTTGAAACTTGGTGGATAGTGTACCCACTACCTTTCTCCACTTAAATATC 2190

SG-7 2161 ATGATTTTGGTTGAGGCAGCGTTCAAAGATGTGATTCGTGTCTAACCAACATACACGTGA 2220

||||||||||||||||||||||||||||||||||||||||||||||||||||||||||||

TS-165 2191 ATGATTTTGGTTGAGGCAGCGTTCAAAGATGTGATTCGTGTCTAACCAACATACACGTGA 2250

SG-7 2221 TGAACGCTGCCACTAGATCAAAGCCCAGGGGCAATGTTGCTAACCTACTGTTTATGATAA 2280

||||||||||||||||||||||||||||||||||||||||||||||||||||||||||||

TS-165 2251 TGAACGCTGCCACTAGATCAAAGCCCAGGGGCAATGTTGCTAACCTACTGTTTATGATAA 2310

SG-7 2281 CACGTGGATATGCTTCTGTCAAGATTGTGATTCCACACCATTAATAACGAAACAACATTT 2340

||||||||||||||||||||||||||||||||||||||||||||||||||||||||||||

TS-165 2311 CACGTGGATATGCTTCTGTCAAGATTGTGATTCCACACCATTAATAACGAAACAACATTT 2370

SG-7 2341 ATAAGTAACAAATCCCTCTCGGAAGGTCTAAAGTAGTTCATAGAGAATGGTTGACCTTAT 2400

||||||||||||||||||||||||||||||||||||||||||||||||||||||||||||

TS-165 2371 ATAAGTAACAAATCCCTCTCGGAAGGTCTAAAGTAGTTCATAGAGAATGGTTGACCTTAT 2430

SG-7 2401 TTTCATATGATATGTCCAAAAGTATATGTGCATTGACATCAAACACGAAATATCAAAACA 2460

||||||||||||||||||||||||||||||||||||||||||||||||||||||||||||

TS-165 2431 TTTCATATGATATGTCCAAAAGTATATGTGCATTGACATCAAACACGAAATATCAAAACA 2490

SG-7 2461 ATAAGATCCATAAATCTATAGCATATCATAACAGCCTAAAGATATTGGAAAATGACAAAA 2520

||||||||||||||||||||||||||||||||||||||||||||||||||||||||||||

TS-165 2491 ATAAGATCCATAAATCTATAGCATATCATAACAGCCTAAAGATATTGGAAAATGACAAAA 2550

SG-7 2521 CTCAGCAGATTCAAACTGAATTGCTGTATGTCATTGGCATATCGCCGTAAAGTATCGAAT 2580

||||||||||||||||||||||||||||||||||||||||||||||||||||||||||||

TS-165 2551 CTCAGCAGATTCAAACTGAATTGCTGTATGTCATTGGCATATCGCCGTAAAGTATCGAAT 2610

SG-7 2581 CCTTGTCTTACCATTCACTGCAGCAGGCAATGTGTAGTCCACTACAATTAGATTAGGGTG 2640

||||||||||||||||||||||||||||||||||||||||||||||||||||||||||||

TS-165 2611 CCTTGTCTTACCATTCACTGCAGCAGGCAATGTGTAGTCCACTACAATTAGATTAGGGTG 2670

SG-7 2641 TTCCTCAAAGACAGAGGACAAGAGGTTTTCTCTATCCGAAGGACCATGCACAATTATCTC 2700

||||||||||||||||||||||||||||||||||||||||||||||||||||||||||||

TS-165 2671 TTCCTCAAAGACAGAGGACAAGAGGTTTTCTCTATCCGAAGGACCATGCACAATTATCTC 2730

SG-7 2701 TTTTCCACCCGCCTCCACAGTTTTTCCTTCATCTTGTGGACCACCAAAACATATAGGCAC 2760

||||||||||||||||||||||||||||||||||||||||||||||||||||||||||||

TS-165 2731 TTTTCCACCCGCCTCCACAGTTTTTCCTTCATCTTGTGGACCACCAAAACATATAGGCAC 2790

SG-7 2761 AGGTTGAAGTCCCGCAGCGATGGATGCTTCAAGAACTGCCCTCCCCATTTTGCCAGTACA 2820

||||||||||||||||||||||||||||||||||||||||||||||||||||||||||||

TS-165 2791 AGGTTGAAGTCCCGCAGCGATGGATGCTTCAAGAACTGCCCTCCCCATTTTGCCAGTACA 2850

SG-7 2821 ACCATTCACCTTTACATCAAAATAAAACAAACCTAACTCAAACAGTATTACCTTGATAAA 2880

||||||||||||||||||||||||||||||||||||||||||||||||||||||||||||

TS-165 2851 ACCATTCACCTTTACATCAAAATAAAACAAACCTAACTCAAACAGTATTACCTTGATAAA 2910

SG-7 2881 ACTAGAAATCAATTAACAACAGTGTAATTTAAAATCATTGGCAAACATATTAATTaaaaa 2940

||||||||||||||||||||||||||||||||||||||||||||||||||||||||||||

TS-165 2911 ACTAGAAATCAATTAACAACAGTGTAATTTAAAATCATTGGCAAACATATTAATTAAAAA 2970

SG-7 2941 aaaGGAAGGAACCTACCATTATAGGGAGTGGCTCGATTTTTGATTTAACTAAAACCTCAA 3000

||||||||||||||||||||||||||||||||||||||||||||||||||||||||||||

TS-165 2971 AAAGGAAGGAACCTACCATTATAGGGAGTGGCTCGATTTTTGATTTAACTAAAACCTCAA 3030

SG-7 3001 CATTTTGTGCTGGAGCAGAACACATCTTCACCGAATATTTTCGACCTCGAACAAAACTAT 3060

||||||||||||||||||||||||||||||||||||||||||||||||||||||||||||

TS-165 3031 CATTTTGTGCTGGAGCAGAACACATCTTCACCGAATATTTTCGACCTCGAACAAAACTAT 3090

SG-7 3061 TGCAGCCATTTTTGCTGCTTGAACTGTAACGCATTCCTCCTCCATTAATGTAAAGTGGAA 3120

||||||||||||||||||||||||||||||||||||||||||||||||||||||||||||

TS-165 3091 TGCAGCCATTTTTGCTGCTTGAACTGTAACGCATTCCTCCTCCATTAATGTAAAGTGGAA 3150

SG-7 3121 CTTTGATGATAGCCCACATTGTTTTACTGCAATACAATGAGAAAGCGCACACAAATTATA 3180

||||||||||||||||||||||||||||||||||||||||||||||||||||||||||||

TS-165 3151 CTTTGATGATAGCCCACATTGTTTTACTGCAATACAATGAGAAAGCGCACACAAATTATA 3210

SG-7 3181 AAGATTTTGACTCGTCAGCAAAGACACTCTAGGGTTTTAAAAATTATCGGCCAAGTGTAG 3240

||||||||||||||||||||||||||||||||||||||||||||||||||||||||||||

TS-165 3211 AAGATTTTGACTCGTCAGCAAAGACACTCTAGGGTTTTAAAAATTATCGGCCAAGTGTAG 3270

SG-7 3241 GTGTTATTTTAGTTTTTAGGACATTGCTATAAAATTTGTATAAATGTTGTACTTCATTAA 3300

||||||||||||||||||||||||||||||||||||||||||||||||||||||||||||

TS-165 3271 GTGTTATTTTAGTTTTTAGGACATTGCTATAAAATTTGTATAAATGTTGTACTTCATTAA 3330

SG-7 3301 GTTTAATTTTACAAAATGAATAGTATTATGGAAATTGTAAAATATTTTGTGTAAATCAAG 3360

||||||||||||||||||||||||||||||||||||||||||||||||||||||||||||

TS-165 3331 GTTTAATTTTACAAAATGAATAGTATTATGGAAATTGTAAAATATTTTGTGTAAATCAAG 3390

SG-7 3361 TTCTGTAATAAACGATAGTAATAGAATTATTGGAGAAAATTAAATTGAtttttttAAAAT 3420

||||||||||||||||||||||||||||||||||||||||||||||||||||||||||||

TS-165 3391 TTCTGTAATAAACGATAGTAATAGAATTATTGGAGAAAATTAAATTGATTTTTTTAAAAT 3450

SG-7 3421 AATAATATAACAGTCCTTTTGATACTTTAATTTTAGAGATTATAAAAATAAGTACCTTGA 3480

||||||||||||||||||||||||||||||||||||||||||||||||||||||||||||

TS-165 3451 AATAATATAACAGTCCTTTTGATACTTTAATTTTAGAGATTATAAAAATAAGTACCTTGA 3510

SG-7 3481 TTTTATAATCTCAAAGACTTGCTCTTTGATTTATGAATCAATAAACGTAATAAAGATAAA 3540

||||||||||||||||||||||||||||||||||||||||||||||||||||||||||||

TS-165 3511 TTTTATAATCTCAAAGACTTGCTCTTTGATTTATGAATCAATAAACGTAATAAAGATAAA 3570

SG-7 3541 ATAATTAGTTTAAAGAAAAGGAAGATGTAAAAGAGGATCTCGAATCATTGTTCtttaaaa 3600

||||||||||||||||||||||||||||||||||||||||||||||||||||||||||||

TS-165 3571 ATAATTAGTTTAAAGAAAAGGAAGATGTAAAAGAGGATCTCGAATCATTGTTCTTTAAAA 3630

SG-7 3601 taaaattaaaattcatttttaaaaaaaTGAGTGTGTGtatatatatatatatGGAAAGTG 3660

||||||||||||||||||||||||||||||||||||||||||||||||||||||||||||

TS-165 3631 TAAAATTAAAATTCATTTTTAAAAAAATGAGTGTGTGTATATATATATATATGGAAAGTG 3690

SG-7 3661 AGAATAAGTATAAAAAGGGAGAGATAAATGCTTTAATTAATTAACaaaaaaaaGGGAAAT 3720

||||||||||||||||||||||||||||||||||||||||||||||||||||||||||||

TS-165 3691 AGAATAAGTATAAAAAGGGAGAGATAAATGCTTTAATTAATTAACAAAAAAAAGGGAAAT 3750

SG-7 3721 TTATCTAATTTGCTTATCCACTCTGAAAAATAAACCAAATCCAAtttttttttaaaaaaa 3780

||||||||||||||||||||||||||||||||||||||||||||||||||||||||||||

TS-165 3751 TTATCTAATTTGCTTATCCACTCTGAAAAATAAACCAAATCCAATTTTTTTTTAAAAAAA 3810

SG-7 3781 aaaCAACATAAAATTAGTAGTAGTAACTTAGATTCGTGGGCCTTAATATATTGGGGCCTA 3840

||||||||||||||||||||||||||||||||||||||||||||||||||||||||||||

TS-165 3811 AAACAACATAAAATTAGTAGTAGTAACTTAGATTCGTGGGCCTTAATATATTGGGGCCTA 3870

SG-7 3841 AACCCAAGGTTTTAACAGCGTCGTGGTCTGGTTGGCCCGCTATACTTTATAACGGtatat 3900

||||||||||||||||||||||||||||||||||||||||||||||||||||||||||||

TS-165 3871 AACCCAAGGTTTTAACAGCGTCGTGGTCTGGTTGGCCCGCTATACTTTATAACGGTATAT 3930

SG-7 3901 tttattttatttttatatgagattttttttataaaaaaaatatatgagatttaaaatact 3960

||||||||||||||||||||||||||||||||||||||||||||||||||||||||||||

TS-165 3931 TTTATTTTATTTTTATATGAGATTTTTTTTATAAAAAAAATATATGAGATTTAAAATACT 3990

SG-7 3961 aattaacaaaaaatatatatagataatGTCTCCTTTTAATGAAGATTATCAAAATGCTAC 4020

||||||||||||||||||||||||||||||||||||||||||||||||||||||||||||

TS-165 3991 AATTAACAAAAAATATATATAGATAATGTCTCCTTTTAATGAAGATTATCAAAATGCTAC 4050

SG-7 4021 ATTATATTAATAGTATTCAATATAATTTTTAAAAACAAGGGCTCTAATCATTGATTAGAG 4080

||||||||||||||||||||||||||||||||||||||||||||||||||||||||||||

TS-165 4051 ATTATATTAATAGTATTCAATATAATTTTTAAAAACAAGGGCTCTAATCATTGATTAGAG 4110

SG-7 4081 CCCTTGTTTTTAAAACTTGTGTTTATTTTGATTCGTGATTAATTCATACAATAGCGATCA 4140

||||||||||||||||||||||||||||||||||||||||||||||||||||||||||||

TS-165 4111 CCCTTGTTTTTAAAACTTGTGTTTATTTTGATTCGTGATTAATTCATACAATAGCGATCA 4170

SG-7 4141 AAAACAAATAGGTATTCAAAACAAGTAAATGTTTAATTCAGTTATATTTTTAATTATGCT 4200

||||||||||||||||||||||||||||||||||||||||||||||||||||||||||||

TS-165 4171 AAAACAAATAGGTATTCAAAACAAGTAAATGTTTAATTCAGTTATATTTTTAATTATGCT 4230

SG-7 4201 ATTTTTATTCATGAAGTTTGACCTTCTCTAATTTATCTGTTTTCTTTATTATCTTTGCAT 4260

||||||||||||||||||||||||||||||||||||||||||||||||||||||||||||

TS-165 4231 ATTTTTATTCATGAAGTTTGACCTTCTCTAATTTATCTGTTTTCTTTATTATCTTTGCAT 4290

SG-7 4261 TTTCTTTGAAGACTTAAATTCAAATTTAATTTGTCTGAAGATCAGAAAAAATTATATCAA 4320

||||||||||||||||||||||||||||||||||||||||||||||||||||||||||||

TS-165 4291 TTTCTTTGAAGACTTAAATTCAAATTTAATTTGTCTGAAGATCAGAAAAAATTATATCAA 4350

SG-7 4321 ATACAGTATTTGAAATGCTGAATTTCATACGAAACAACCAACAAACGGAAACTATTCAAC 4380

||||||||||||||||||||||||||||||||||||||||||||||||||||||||||||

TS-165 4351 ATACAGTATTTGAAATGCTGAATTTCATACGAAACAACCAACAAACGGAAACTATTCAAC 4410

SG-7 4381 CCTACTCATAATCAACAACCCAAACGACCCCAAAATTGTTGTTTGATATATAGTGATCTT 4440

||||||||||||||||||||||||||||||||||||||||||||||||||||||||||||

TS-165 4411 CCTACTCATAATCAACAACCCAAACGACCCCAAAATTGTTGTTTGATATATAGTGATCTT 4470

SG-7 4441 CTTAACATTTTGCAAACAAAAATATGGAATAAATATAAATAAATATGCGACTAATAATTG 4500

||||||||||||||||||||||||||||||||||||||||||||||||||||||||||||

TS-165 4471 CTTAACATTTTGCAAACAAAAATATGGAATAAATATAAATAAATATGCGACTAATAATTG 4530

SG-7 4501 TAACAAGATTTAATCCCTAATTTATATGGTCTTTCACCATTGGATGTGGTCATTATGATC 4560

||||||||||||||||||||||||||||||||||||||||||||||||||||||||||||

TS-165 4531 TAACAAGATTTAATCCCTAATTTATATGGTCTTTCACCATTGGATGTGGTCATTATGATC 4590

SG-7 4561 AAGCACTATTACAAGCTGGGAAAGGGAAAGGGACAATCGATGGATATTTAGTGAAAATAT 4620

||||||||||||||||||||||||||||||||||||||||||||||||||||||||||||

TS-165 4591 AAGCACTATTACAAGCTGGGAAAGGGAAAGGGACAATCGATGGATATTTAGTGAAAATAT 4650

SG-7 4621 TACTATaaaaaaaTATTTTGATATGACAAAAGAAACAACTGGCTAGCCTTATAGTATAAT 4680

||||||||||||||||||||||||||||||||||||||||||||||||||||||||||||

TS-165 4651 TACTATAAAAAAATATTTTGATATGACAAAAGAAACAACTGGCTAGCCTTATAGTATAAT 4710

SG-7 4681 AAAGAAAGTAGATGATTTGACCCTTTCGTCATTTGTTAGCTAGCCCCACAAAGTTACGTA 4740

||||||||||||||||||||||||||||||||||||||||||||||||||||||||||||

TS-165 4711 AAAGAAAGTAGATGATTTGACCCTTTCGTCATTTGTTAGCTAGCCCCACAAAGTTACGTA 4770

SG-7 4741 GATATTAATAATGTAGTAATGATAAAAGAAATAACTAGCTAGCCTTATAGTATCATAATA 4800

||||||||||||||||||||||||||||||||||||||||||||||||||||||||||||

TS-165 4771 GATATTAATAATGTAGTAATGATAAAAGAAATAACTAGCTAGCCTTATAGTATCATAATA 4830

SG-7 4801 AAAATAGATGATTTGATCTTTCGTCATTCGGTAGTTAATCatataaattatgcatatatt 4860

||||||||||||||||||||||||||||||||||||||||||||||||||||||||||||

TS-165 4831 AAAATAGATGATTTGATCTTTCGTCATTCGGTAGTTAATCATATAAATTATGCATATATT 4890

SG-7 4861 agtaatgtatattaagtattagttatatagggtttaattatgtaagatttatttatacat 4920

||||||||||||||||||||||||||||||||||||||||||||||||||||||||||||

TS-165 4891 AGTAATGTATATTAAGTATTAGTTATATAGGGTTTAATTATGTAAGATTTATTTATACAT 4950

SG-7 4921 acatattatatagatattagttatctagagtttaattatatgaaattattatacattatt 4980

||||||||||||||||||||||||||||||||||||||||||||||||||||||||||||

TS-165 4951 ACATATTATATAGATATTAGTTATCTAGAGTTTAATTATATGAAATTATTATACATTATT 5010

SG-7 4981 GAGCAATTTTTATTTCTTAATTCCaaaaaaaaTTAGCAAGCTAATATTTATTATGACCAT 5040

||||||||||||||||||||||||||||||||||||||||||||||||||||||||||||

TS-165 5011 GAGCAATTTTTATTTCTTAATTCCAAAAAAAATTAGCAAGCTAATATTTATTATGACCAT 5070

SG-7 5041 TAAAATAATCAACTATATGAATAACACGTTaaaaaaaaGGCAATTTTCACATATAGCAAA 5100

||||||||||||||||||||||||||||||||||||||||||||||||||||||||||||

TS-165 5071 TAAAATAATCAACTATATGAATAACACGTTAAAAAAAAGGCAATTTTCACATATAGCAAA 5130

SG-7 5101 CaaaaaaaTCATATTTGTATAATATAACAAACTTTGCATAATTGCGCTCCATAGCAAAAA 5160

|||||||||||||||||||||||||||||||||||||||||||||||||||||||||| |

TS-165 5131 CAAAAAAATCATATTTGTATAATATAACAAACTTTGCATAATTGCGCTCCATAGCAAACA 5190

SG-7 5161 TAAAAACTGTATAATTCGCTATACATATAAAAGTGTATAATTCGCTGGCCTAAATTGTAT 5220

||||||||||||||||||||||||||||||||||||||||||||||||||||||||||||

TS-165 5191 TAAAAACTGTATAATTCGCTATACATATAAAAGTGTATAATTCGCTGGCCTAAATTGTAT 5250

SG-7 5221 AATTCGCTGGCCTATTTCGCTGCAATTGTATAATTAGCTTTGCATACAGTTAAATCGAAT 5280

||||||||||||||||||||||||||||||||||||||||||||||||||||||||||||

TS-165 5251 AATTCGCTGGCCTATTTCGCTGCAATTGTATAATTAGCTTTGCATACAGTTAAATCGAAT 5310

SG-7 5281 TAAAATGTATGTATATTGCATAATTATAAGTGTATAGCAAGAAGATATATGTTTCACAAT 5340

||||||||||||||||||||||||||||||||||||||||||||||||||||||||||||

TS-165 5311 TAAAATGTATGTATATTGCATAATTATAAGTGTATAGCAAGAAGATATATGTTTCACAAT 5370

SG-7 5341 ATATACACTTCTGTTGTATAAAGCTAGAAAAAATTGTATTTCACTGCAATTGTATAATTC 5400

||||||||||||||||||||||||||||||||||||||||||||||||||||||||||||

TS-165 5371 ATATACACTTCTGTTGTATAAAGCTAGAAAAAATTGTATTTCACTGCAATTGTATAATTC 5430

SG-7 5401 TTTGCCCTTTTTCTCTGCAATATTTGAAGTAAAATGTTTGTAAATTATATAATTAAGTGT 5460

||||||||||||||||||||||||||||||||||||||||||||||||||||||||||||

TS-165 5431 TTTGCCCTTTTTCTCTGCAATATTTGAAGTAAAATGTTTGTAAATTATATAATTAAGTGT 5490

SG-7 5461 ATAACACGAAGATATACATTTTTGCATGTGGATATACAATTTTCTCTCGCTTTATACAAA 5520

||||||||||||||||||||||||||||||||||||||||||||||||||||||||||||

TS-165 5491 ATAACACGAAGATATACATTTTTGCATGTGGATATACAATTTTCTCTCGCTTTATACAAA 5550

SG-7 5521 AACAGAAACAGAAATTATACACTTCTGTGTATAAAgcgagagaggcgagaatgggagagt 5580

||||||||||||||||||||||||||||||||||||||||||||||||||||||||||||

TS-165 5551 AACAGAAACAGAAATTATACACTTCTGTGTATAAAGCGAGAGAGGCGAGAATGGGAGAGT 5610

SG-7 5581 ggcgagcgagacttctgggagagagaCGCCTGACAAATTTTTGCCAACGTTTGCTATGGG 5640

||||||||||||||||||||||||||||||||||||||||||||||||||||||||||||

TS-165 5611 GGCGAGCGAGACTTCTGGGAGAGAGACGCCTGACAAATTTTTGCCAACGTTTGCTATGGG 5670

SG-7 5641 GCACAATTAAATCAAACCCTAACTATTCCATTTAATTTAGGTTATTAGTTTGCTATTTTA 5700

||||||||||||||||||||||||||||||||||||||||||||||||||||||||||||

TS-165 5671 GCACAATTAAATCAAACCCTAACTATTCCATTTAATTTAGGTTATTAGTTTGCTATTTTA 5730

SG-7 5701 TACAATTTTCCCTtaaaaaaataccaatagctaaaaataataaaaataaaatgataaaGG 5760

||||||||||||||||||||||||||||||||||||||||||||||||||||||||||||

TS-165 5731 TACAATTTTCCCTTAAAAAAATACCAATAGCTAAAAATAATAAAAATAAAATGATAAAGG 5790

SG-7 5761 TAATTAAGTAATTGGTATTCAATATGATTAAGTATTTAAGTAATAGGTTATCTAAGTTAt 5820

||||||||||||||||||||||||||||||||||||||||||||||||||||||||||||

TS-165 5791 TAATTAAGTAATTGGTATTCAATATGATTAAGTATTTAAGTAATAGGTTATCTAAGTTAT 5850

SG-7 5821 tttttttaaaaaaaaaaagaaactaaaaaaaTTGAAATGTGTATTGATGGTGCGTTGTaa 5880

|||||| |||||||||||||||||||||||||||||||||||||||||||||||||||||

TS-165 5851 TTTTTTAAAAAAAAAAAAGAAACTAAAAAAATTGAAATGTGTATTGATGGTGCGTTGTAA 5910

SG-7 5881 aaaaaCTATGCAGAGAAATTAATTACAAATAATAATCACTGCCTTGCCACCAAAGATAAC 5940

||||||||||||||||||||||||||||||||||||||||||||||||||||||||||||

TS-165 5911 AAAAACTATGCAGAGAAATTAATTACAAATAATAATCACTGCCTTGCCACCAAAGATAAC 5970

SG-7 5941 AATTAACAAGAATTTAAGGAGATAAAATTATCAAGCAGAAAAACTCACAGTGCttttttt 6000

||||||||||||||||||||||||||||||||||||||||||||||||||||||||||||

TS-165 5971 AATTAACAAGAATTTAAGGAGATAAAATTATCAAGCAGAAAAACTCACAGTGCTTTTTTT 6030

SG-7 6001 ttttACATTCATTAAATATTTGATTTGTTGTAGATTAATACATATTTAAAAGTAAATCGA 6060

||||||||||||||||||||||||||||||||||||||||||||||||||||||||||||

TS-165 6031 TTTTACATTCATTAAATATTTGATTTGTTGTAGATTAATACATATTTAAAAGTAAATCGA 6090

SG-7 6061 TAGTGATAGATATATTATTTACTATTTTAACACATATATAATTAATACACCATAACTTAT 6120

||||||||||||||||||||||||||||||||||||||||||||||||||||||||||||

TS-165 6091 TAGTGATAGATATATTATTTACTATTTTAACACATATATAATTAATACACCATAACTTAT 6150

SG-7 6121 TCCATTTTTCATCCCGCATAAGTTATATATAAATTTTTCATAAGTTATACAAATATTAAA 6180

||||||||||||||||||||||||||||||||||||||||||||||||||||||||||||

TS-165 6151 TCCATTTTTCATCCCGCATAAGTTATATATAAATTTTTCATAAGTTATACAAATATTAAA 6210

SG-7 6181 TATTGATTATGTAGAATTACAAAAAGCGCAAATAAACACCGTATGAAATTAATACATGAA 6240

||||||||||||||||||||||||||||||||||||||||||||||||||||||||||||

TS-165 6211 TATTGATTATGTAGAATTACAAAAAGCGCAAATAAACACCGTATGAAATTAATACATGAA 6270

SG-7 6241 TAATTTTTATATAATATTTAAAAAACTACCAACCAAACATTGTATAAAATTAATACATAA 6300

||||||||||||||||||||||||||||||||||||||||||||||||||||||||||||

TS-165 6271 TAATTTTTATATAATATTTAAAAAACTACCAACCAAACATTGTATAAAATTAATACATAA 6330

SG-7 6301 ATAACTTTTATATAATATTTGAAAACTTACCAACCAAACGTTAAAATTAATTCATGAATA 6360

||||||||||||||||||||||||||||||||||||||||||||||||||||||||||||

TS-165 6331 ATAACTTTTATATAATATTTGAAAACTTACCAACCAAACGTTAAAATTAATTCATGAATA 6390

SG-7 6361 ACTTTTGATTCTAGTTGCAAAACAAACATTATATAAAAATAACACATAAATAACAAAGTA 6420

||||||||||||||||||||||||||||||||||||||||||||||||||||||||||||

TS-165 6391 ACTTTTGATTCTAGTTGCAAAACAAACATTATATAAAAATAACACATAAATAACAAAGTA 6450

SG-7 6421 ATTCATATATTAGATGTAGCTTAAGCCTGCTATCAAATGGCCCTTAATGTCTCCTTAAAA 6480

||||||||||||||||||||||||||||||||||||||||||||||||||||||||||||

TS-165 6451 ATTCATATATTAGATGTAGCTTAAGCCTGCTATCAAATGGCCCTTAATGTCTCCTTAAAA 6510

SG-7 6481 TAAAGATATGAACCTTAAAATGGCCTCATTTtatatatatatatatatatatatatGTTG 6540

||||||||||||||||||||||||||||||||||||||||||||||||||||||||||||

TS-165 6511 TAAAGATATGAACCTTAAAATGGCCTCATTTTATATATATATATATATATATATATGTTG 6570

SG-7 6541 GAACCAATAAATTTGTGGTGGAGTGATATATTTTTCTTTTATGTGTAATTAAAGATCTCA 6600

||||||||||||||||||||||||||||||||||||||||||||||||||||||||||||

TS-165 6571 GAACCAATAAATTTGTGGTGGAGTGATATATTTTTCTTTTATGTGTAATTAAAGATCTCA 6630

SG-7 6601 AGTTTGAGTTCAAGTTTCTCGATGTATGAAGTTGTAGTTACTTGTTTCAATTTTTGCCAT 6660

||||||||||||||||||||||||||||||||||||||||||||||||||||||||||||

TS-165 6631 AGTTTGAGTTCAAGTTTCTCGATGTATGAAGTTGTAGTTACTTGTTTCAATTTTTGCCAT 6690

SG-7 6661 TAAATCAATGACTCACCTTGTTAGAAATTTTCAATAAATATCTTTTATATATTTGATGAA 6720

||||||||||||||||||||||||||||||||||||||||||||||||||||||||||||

TS-165 6691 TAAATCAATGACTCACCTTGTTAGAAATTTTCAATAAATATCTTTTATATATTTGATGAA 6750

SG-7 6721 ATTTTCAATATAAATATAAAATCTACAGAAAAGACATTAATTTTCCAAAAATTCATAACC 6780

||||||||||||||||||||||||||||||||||||||||||||||||||||||||||||

TS-165 6751 ATTTTCAATATAAATATAAAATCTACAGAAAAGACATTAATTTTCCAAAAATTCATAACC 6810

SG-7 6781 ACTTGGATCCGCCCTTGCCTCATAGTTTTGCTAAGTTATTATAATAGTATTATTAACTTA 6840

||||||||||||||||||||||||||||||||||||||||||||||||||||||||||||

TS-165 6811 ACTTGGATCCGCCCTTGCCTCATAGTTTTGCTAAGTTATTATAATAGTATTATTAACTTA 6870

SG-7 6841 TATCTATACTTAATAAAAATTCATAAAATTCAAACTCCAAATCCATCCTAAAATCATATG 6900

||||||||||||||||||||||||||||||||||||||||||||||||||||||||||||

TS-165 6871 TATCTATACTTAATAAAAATTCATAAAATTCAAACTCCAAATCCATCCTAAAATCATATG 6930

SG-7 6901 CTAAATGGAGATCATAGAGGAGGAAACTTGGTGAGGAGACTAATAATTGGGAAGAAGGCA 6960

||||||||||||||||||||||||||||||||||||||||||||||||||||||||||||

TS-165 6931 CTAAATGGAGATCATAGAGGAGGAAACTTGGTGAGGAGACTAATAATTGGGAAGAAGGCA 6990

SG-7 6961 AGATTGTTTTAGAATGAATGAATGAAGTAAAAAGGATTAGGTGTTTAGTTTTTGTCCACA 7020

||||||||||||||||||||||||||||||||||||||||||||||||||||||||||||

TS-165 6991 AGATTGTTTTAGAATGAATGAATGAAGTAAAAAGGATTAGGTGTTTAGTTTTTGTCCACA 7050

SG-7 7021 ACATGAAAGACCAATATGAAGTACAGTGAGGTGCAATGCCACaaaaaaaaTAAATTGAGT 7080

||||||||||||||||||||||||||||||||||||||||||||||||||||||||||||

TS-165 7051 ACATGAAAGACCAATATGAAGTACAGTGAGGTGCAATGCCACAAAAAAAATAAATTGAGT 7110

SG-7 7081 ACTCACTTATTTCCAGCTAGGAGCTTTAATGCCATTTGTACTTATATTCCATCAATCATT 7140

||||||||||||||||||||||||||||||||||||||||||||||||||||||||||||

TS-165 7111 ACTCACTTATTTCCAGCTAGGAGCTTTAATGCCATTTGTACTTATATTCCATCAATCATT 7170

SG-7 7141 TCTACCTACTTCAACACTTATAATGAAACTTGCAACCTGTTTTCTTGTCTACAGGATGTT 7200

||||||||||||||||||||||||||||||||||||||||||||||||||||||||||||

TS-165 7171 TCTACCTACTTCAACACTTATAATGAAACTTGCAACCTGTTTTCTTGTCTACAGGATGTT 7230

SG-7 7201 TCTTTGGCTTTTCCAATTCATTTATATGACATTTTGTTACAAAAAACTACACAAGAATCA 7260

||||||||||||||||||||||||||||||||||||||||||||||||||||||||||||

TS-165 7231 TCTTTGGCTTTTCCAATTCATTTATATGACATTTTGTTACAAAAAACTACACAAGAATCA 7290

SG-7 7261 TATGCACAGAGTAAAAGAACTAGGTCATTTGCAAACAACTGAAAAGATAATTGTGAAGCA 7320

||||||||||||||||||||||||||||||||||||||||||||||||||||||||||||

TS-165 7291 TATGCACAGAGTAAAAGAACTAGGTCATTTGCAAACAACTGAAAAGATAATTGTGAAGCA 7350

SG-7 7321 AGTTTCAAGTGAATTTACTATGAGATAAACACATGAATTGATAAATACAAATTCATCATG 7380

||||||||||||||||||||||||||||||||||||||||||||||||||||||||||||

TS-165 7351 AGTTTCAAGTGAATTTACTATGAGATAAACACATGAATTGATAAATACAAATTCATCATG 7410

SG-7 7381 GCAATATTGTTATGCTTGCACAAAAGGAAAGAACTTTGAAATTAGGGAAAATTGTGTGAA 7440

||||||||||||||||||||||||||||||||||||||||||||||||||||||||||||

TS-165 7411 GCAATATTGTTATGCTTGCACAAAAGGAAAGAACTTTGAAATTAGGGAAAATTGTGTGAA 7470

SG-7 7441 AAATCGAAGTAAATAAAGGAGTCTTAGTCTTATTAAATTCGTTGACCAAATTTCAATGAG 7500

||||||||||||||||||||||||||||||||||||||||||||||||||||||||||||

TS-165 7471 AAATCGAAGTAAATAAAGGAGTCTTAGTCTTATTAAATTCGTTGACCAAATTTCAATGAG 7530

SG-7 7501 AGTTGGAGCAAGTCAACAGAATCACAATCATCACTACTCCTATATAAAACATCATTTTGA 7560

||||||||||||||||||||||||||||||||||||||||||||||||||||||||||||

TS-165 7531 AGTTGGAGCAAGTCAACAGAATCACAATCATCACTACTCCTATATAAAACATCATTTTGA 7590

SG-7 7561 TGCTACTAAACACATGTTATTTCACACAAACAAGGACTTGATCTTGGCAATAACAATTAT 7620

||||||||||||||||||||||||||||||||||||||||||||||||||||||||||||

TS-165 7591 TGCTACTAAACACATGTTATTTCACACAAACAAGGACTTGATCTTGGCAATAACAATTAT 7650

SG-7 7621 TGTGAGAGATAGAGCATAATAATTCAAACTCATCAACAAGTTGTTGAGGTTGTTGAATCC 7680

||||||||||||||||||||||||||||||||||||||||||||||||||||||||||||

TS-165 7651 TGTGAGAGATAGAGCATAATAATTCAAACTCATCAACAAGTTGTTGAGGTTGTTGAATCC 7710

SG-7 7681 ATCCCATTTTTTAGTCAACTTTAACTATTCAATTGGCATAAACAATTGTTAAAAAGGAAT 7740

||||||||||||||||||||||||||||||||||||||||||||||||||||||||||||

TS-165 7711 ATCCCATTTTTTAGTCAACTTTAACTATTCAATTGGCATAAACAATTGTTAAAAAGGAAT 7770

SG-7 7741 AGTAATTGGAGTTGAAAAATAAAATATTTGGTAGTTGACAAATTGGTAAGGTTTGCAACA 7800

||||||||||||||||||||||||||||||||||||||||||||||||||||||||||||

TS-165 7771 AGTAATTGGAGTTGAAAAATAAAATATTTGGTAGTTGACAAATTGGTAAGGTTTGCAACA 7830

SG-7 7801 ATTCTTGACTTTGTTATATTTACATATGTCATTAGTGACATAAGCATGGTTTTATTCTTC 7860

||||||||||||||||||||||||||||||||||||||||||||||||||||||||||||

TS-165 7831 ATTCTTGACTTTGTTATATTTACATATGTCATTAGTGACATAAGCATGGTTTTATTCTTC 7890

SG-7 7861 TATAAATAGAGCATTCTTGCTTATTTGTAGAACACACCAAGTTAGAGAGAAAAATCATTT 7920

||||||||||||||||||||||||||||||||||||||||||||||||||||||||||||

TS-165 7891 TATAAATAGAGCATTCTTGCTTATTTGTAGAACACACCAAGTTAGAGAGAAAAATCATTT 7950

SG-7 7921 TGAAAACAAAGTGAGGTATTCCATAGATTATACAAATAAATAGTCTATGaaaaaaaaTAA 7980

||||||||||||||||||||||||||||||||||||||||||||||||||||||||||||

TS-165 7951 TGAAAACAAAGTGAGGTATTCCATAGATTATACAAATAAATAGTCTATGAAAAAAAATAA 8010

SG-7 7981 AATGTGAGCGATATTTTAGTAAGATGGAAATCAAAAGAGTACTGAACTTTTTGAGAGTCT 8040

||||||||||||||||||||||||||||||||||||||||||||||||||||||||||||

TS-165 8011 AATGTGAGCGATATTTTAGTAAGATGGAAATCAAAAGAGTACTGAACTTTTTGAGAGTCT 8070

SG-7 8041 TACCTTCTAGAATAGGAGGAATCATGTTGTCTTGAAAAAGGTTGATGCAATTGGTTAAAT 8100

||||||||||||||||||||||||||||||||||||||||||||||||||||||||||||

TS-165 8071 TACCTTCTAGAATAGGAGGAATCATGTTGTCTTGAAAAAGGTTGATGCAATTGGTTAAAT 8130

SG-7 8101 TGTAAAGGTAGTCTCTTTGATTATAAAGTTAATGACTTCAATTTAAGTGAATTTTAGAAG 8160

||||||||||||||||||||||||||||||||||||||||||||||||||||||||||||

TS-165 8131 TGTAAAGGTAGTCTCTTTGATTATAAAGTTAATGACTTCAATTTAAGTGAATTTTAGAAG 8190

SG-7 8161 TAAATCTTAAGAAAGTAAGTTGTAATTTATACTTTCATAAGTAAAGTAttttttttatca 8220

||||||||||||||||||||||||||||||||||||||||||||||||||||||||||||

TS-165 8191 TAAATCTTAAGAAAGTAAGTTGTAATTTATACTTTCATAAGTAAAGTATTTTTTTTATCA 8250

SG-7 8221 taaagtttcttgtgttatttatatttcataatttatttattattttGTGACGGAAGAGTC 8280

||||||||||||||||||||||||||||||||||||||||||||||||||||||||||||

TS-165 8251 TAAAGTTTCTTGTGTTATTTATATTTCATAATTTATTTATTATTTTGTGACGGAAGAGTC 8310

SG-7 8281 CAAtttttttttttAGCCCACTGAGTGGTTGCTAAAATAACAATATGTTTCCCAACTTGT 8340

||||||||||||||||||||||||||||||||||||||||||||||||||||||||||||

TS-165 8311 CAATTTTTTTTTTTAGCCCACTGAGTGGTTGCTAAAATAACAATATGTTTCCCAACTTGT 8370

SG-7 8341 TGATTAATTTTATTTTCATTTCTAGAGTTGTTAATGTGAACTATCATATTTTATCCAAGT 8400

||||||||||||||||||||||||||||||||||||||||||||||||||||||||||||

TS-165 8371 TGATTAATTTTATTTTCATTTCTAGAGTTGTTAATGTGAACTATCATATTTTATCCAAGT 8430

SG-7 8401 TAGTTTATACATGTTTTAAAATATGATGATTATGTCAAGCTGGTTCATGTTTTTGTATGT 8460

||||||||||||||||||||||||||||||||||||||||||||||||||||||||||||

TS-165 8431 TAGTTTATACATGTTTTAAAATATGATGATTATGTCAAGCTGGTTCATGTTTTTGTATGT 8490

SG-7 8461 CAGAAAATAGTCGGTCTATCTCATCAGTATGTGGATTGTGGGCTATGTCAtttttttaaa 8520

||||||||||||||||||||||||||||||||||||||||||||||||||||||||||||

TS-165 8491 CAGAAAATAGTCGGTCTATCTCATCAGTATGTGGATTGTGGGCTATGTCATTTTTTTAAA 8550

SG-7 8521 aaaaCATatattttttaattaaattattaacgtaaatatcgataagatattttcatttta 8580

||||||||||||||||||||||||||||||||||||||||||||||||||||||||||||

TS-165 8551 AAAACATATATTTTTTAATTAAATTATTAACGTAAATATCGATAAGATATTTTCATTTTA 8610

SG-7 8581 atctatattttataTGCAAATTCAATTATTTAAACATTAAAAATACCTAAATTTAAGAAC 8640

||||||||||||||||||||||||||||||||||||||||||||||||||||||||||||

TS-165 8611 ATCTATATTTTATATGCAAATTCAATTATTTAAACATTAAAAATACCTAAATTTAAGAAC 8670

SG-7 8641 TACATATAATAATTAAATGTACACAACAAATATTAATAAGTTTGAGAAAAATCATATCAC 8700

||||||||||||||||||||||||||||||||||||||||||||||||||||||||| ||

TS-165 8671 TACATATAATAATTAAATGTACACAACAAATATTAATAAGTTTGAGAAAAATCATATTAC 8730

SG-7 8701 CGGTTCCGAGTACCTACAAATATTTGATTATTTTCATGATGAATGCATGGTAAGAACACA 8760

||||||||||||||||||||||||||||||||||||||||||||||||||||||||||||

TS-165 8731 CGGTTCCGAGTACCTACAAATATTTGATTATTTTCATGATGAATGCATGGTAAGAACACA 8790

SG-7 8761 TCTACCAATGCTGAATGATGATTCTGATGATACAATAGTAATAGAAATAGGTACATCACG 8820

||||||||||||||||||||||||||||||||||||||||||||||||||||||||||||

TS-165 8791 TCTACCAATGCTGAATGATGATTCTGATGATACAATAGTAATAGAAATAGGTACATCACG 8850

SG-7 8821 TTTGATGATTTTAAATTTCAATATTTCAAACttttaatagcggttgagtttgaacttttg 8880

||||||||||||||||||||||||||||||||||||||||||||||||||||||||||||

TS-165 8851 TTTGATGATTTTAAATTTCAATATTTCAAACTTTTAATAGCGGTTGAGTTTGAACTTTTG 8910

SG-7 8881 caatttttaatagtttattgtatttttaattttaatcatctctttatttaGCTCACAGAC 8940

||||||||||||||||||||||||||||||||||||||||||||||||||||||||||||

TS-165 8911 CAATTTTTAATAGTTTATTGTATTTTTAATTTTAATCATCTCTTTATTTAGCTCACAGAC 8970

SG-7 8941 CAACTCAACTCATATTACTTAACGCACACACACCAACATAATTATTTGGGTTGAATTAAA 9000

||||||||||||||||||||||||||||||||||||||||||||||||||||||||||||

TS-165 8971 CAACTCAACTCATATTACTTAACGCACACACACCAACATAATTATTTGGGTTGAATTAAA 9030

SG-7 9001 AAGTTTCtttttttAAATGAATTCCCAAATTTTAGATCAATCCTATTGAATCACGAGTTA 9060

||||||||||||||||||||||||||||||||||||||||||||||||||||||||||||

TS-165 9031 AAGTTTCTTTTTTTAAATGAATTCCCAAATTTTAGATCAATCCTATTGAATCACGAGTTA 9090

SG-7 9061 GATCAGATCAATCCAACATATCTTTCCTTTCATATTTATACTGACATATTTCCCCTCCAC 9120

||||||||||||||||||||||||||||||||||||||||||||||||||||||||||||

TS-165 9091 GATCAGATCAATCCAACATATCTTTCCTTTCATATTTATACTGACATATTTCCCCTCCAC 9150

SG-7 9121 TTTAACTTAATTATAAAATTAAATTTTGTTAAATGACATAATCATAAAAGACCTATATCG 9180

||||||||||||||||||||||||||||||||||||||||||||||||||||||||||||

TS-165 9151 TTTAACTTAATTATAAAATTAAATTTTGTTAAATGACATAATCATAAAAGACCTATATCG 9210

SG-7 9181 GTCGGCCCCATACGAAGACATATTTAATGCCACACGCATACCCACCCCTCCGTTTCTTGT 9240

||||||||||||||||||||||||||||||||||||||||||||||||||||||||||||

TS-165 9211 GTCGGCCCCATACGAAGACATATTTAATGCCACACGCATACCCACCCCTCCGTTTCTTGT 9270

SG-7 9241 TTGCGGAAAGGCAAATATTTTTGGTAAAAAACGTAACTAATTTTCTCCCTTTCTTTATCC 9300

||||||||||||||||||||||||||||||||||||||||||||||||||||||||||||

TS-165 9271 TTGCGGAAAGGCAAATATTTTTGGTAAAAAACGTAACTAATTTTCTCCCTTTCTTTATCC 9330

SG-7 9301 AATTTAGGCCACACCCACATTTAAGAAAACTCCATGGGTATATGTACTTCATTTTTCTCA 9360

||||||||||||||||||||||||||||||||||||||||||||||||||||||||||||

TS-165 9331 AATTTAGGCCACACCCACATTTAAGAAAACTCCATGGGTATATGTACTTCATTTTTCTCA 9390

SG-7 9361 AAAGGGATACGTGTACAATAACATACCAAATAATGCATAAAATGTATTGATAAAAATAGT 9420

||||||||||||||||||||||||||||||||||||||||||||||||||||||||||||

TS-165 9391 AAAGGGATACGTGTACAATAACATACCAAATAATGCATAAAATGTATTGATAAAAATAGT 9450

SG-7 9421 ATTTTAAGCCAAAATAACATAAACGTAACAAAAATAAGAAAATAACAAGATGATACACAC 9480

||||||||||||||||||||||||||||||||||||||||||||||||||||||||||||

TS-165 9451 ATTTTAAGCCAAAATAACATAAACGTAACAAAAATAAGAAAATAACAAGATGATACACAC 9510

SG-7 9481 GTGTCGCATCCGTAGGTCCGTTTGTCGTCACGTGCCTTATGGCGTTCCCCCCACGCTTTT 9540

||||||||||||||||||||||||||||||||||||||||||||||||||||||||||||

TS-165 9511 GTGTCGCATCCGTAGGTCCGTTTGTCGTCACGTGCCTTATGGCGTTCCCCCCACGCTTTT 9570

SG-7 9541 GTCCCTCAAAGGACGCCGTTCACTACTTTTTGTCttttttttCCAACAATTTTGGCAATA 9600

||||||||||||||||||||||||||||||||||||||||||||||||||||||||||||

TS-165 9571 GTCCCTCAAAGGACGCCGTTCACTACTTTTTGTCTTTTTTTTCCAACAATTTTGGCAATA 9630

SG-7 9601 AATCAACACTCTCGGGGCCATCTCATGAATACCCCATACTTTAAGTCCTCATCCCCGAAA 9660

||||||||||||||||||||||||||||||||||||||||||||||||||||||||||||

TS-165 9631 AATCAACACTCTCGGGGCCATCTCATGAATACCCCATACTTTAAGTCCTCATCCCCGAAA 9690

SG-7 9661 CCTAGCAATTTGCTAAATATTACTATCTGGGTCCTACATTCCTTACCCAAAACTTTATTA 9720

||||||||||||||||||||||||||||||||||||||||||||||||||||||||||||

TS-165 9691 CCTAGCAATTTGCTAAATATTACTATCTGGGTCCTACATTCCTTACCCAAAACTTTATTA 9750

SG-7 9721 CTCCTTTAACCCCTCAATTTGTCGTTTATCTACTCTACCACCCTGTGTGTACTTTAGTGT 9780

||||||||||||||||||||||||||||||||||||||||||||||||||||||||||||

TS-165 9751 CTCCTTTAACCCCTCAATTTGTCGTTTATCTACTCTACCACCCTGTGTGTACTTTAGTGT 9810

SG-7 9781 TCGCCCCTTTCTCATTGCTATTCTGCGCCTCGAATCCTTATCCTTCACCTCCCCCTTCAT 9840

||||||||||||||||||||||||||||||||||||||||||||||||||||||||||||

TS-165 9811 TCGCCCCTTTCTCATTGCTATTCTGCGCCTCGAATCCTTATCCTTCACCTCCCCCTTCAT 9870

SG-7 9841 TTTCTTGTTGGTTCATACCCATTTATTTCTCTCCTTGTTGTTAATGAGCTGAAGGAACAG 9900

||||||||||||||||||||||||||||||||||||||||||||||||||||||||||||

TS-165 9871 TTTCTTGTTGGTTCATACCCATTTATTTCTCTCCTTGTTGTTAATGAGCTGAAGGAACAG 9930

SG-7 9901 TGTGTCGAGAGGGAGATGAAAGTACTACTGACAAAGAAAGAAGCTTTATTGTTGTTGGGG 9960

||||||||||||||||||||||||||||||||||||||||||||||||||||||||||||

TS-165 9931 TGTGTCGAGAGGGAGATGAAAGTACTACTGACAAAGAAAGAAGCTTTATTGTTGTTGGGG 9990

SG-7 9961 TTTTTCATGGAAATGTTCTTTCTTGGTTTTGTTTAATCGCTTGTGCtttttttttttttt 10020

||||||||||||||||||||||||||||||||||||||||||||||||||||||||||||

TS-165 9991 TTTTTCATGGAAATGTTCTTTCTTGGTTTTGTTTAATCGCTTGTGCTTTTTTTTTTTTTT 10050

SG-7 10021 tGCTTTTCACCAAATCAAGAAAAAGGAGAAGCGGCTGTCT**ATG**GCAAGAAATAATGAAGC 10080

||||||||||||||||||||||||||||||||||||||||**|||**|||||||||||||||||

TS-165 10051 TGCTTTTCACCAAATCAAGAAAAAGGAGAAGCGGCTGTCT**ATG**GCAAGAAATAATGAAGC 10110

SG-7 10081 TGTTGCGAATGAATCCAAGAGTGAATCTAAGGTACTCCGCTTTTTTCCTGTTTTCTTTCT 10140

||||||||||||||||||||||||||||||||||||||||||||||||||||||||||||

TS-165 10111 TGTTGCGAATGAATCCAAGAGTGAATCTAAGGTACTCCGCTTTTTTCCTGTTTTCTTTCT 10170

SG-7 10141 CTGATCATCAATTTGATTTCTGTTGATTTATGGGTTGGCTTCAAGATTTTGGGTTTTACA 10200

||||||||||||||||||||||||||||||||||||||||||||||||||||||||||||

TS-165 10171 CTGATCATCAATTTGATTTCTGTTGATTTATGGGTTGGCTTCAAGATTTTGGGTTTTACA 10230

SG-7 10201 GTATAGCTATGGTTTGGAGTGATTTTGTCATGCAGATTTTGAGGGGTAGGGTTGTGTTTT 10260

||||||||||||||||||||||||||||||||||||||||||||||||||||||||||||

TS-165 10231 GTATAGCTATGGTTTGGAGTGATTTTGTCATGCAGATTTTGAGGGGTAGGGTTGTGTTTT 10290

SG-7 10261 ACTCTGAATTTTGAAATTTGGTACTTTTGAAAGGATGtttttttttGCTTCTATTTTTGG 10320

||||||||||||||||||||||||||||||||||||||||||||||||||||||||||||

TS-165 10291 ACTCTGAATTTTGAAATTTGGTACTTTTGAAAGGATGTTTTTTTTTGCTTCTATTTTTGG 10350

SG-7 10321 TCTATCAGTGCCCTGTACCCCACAAATTTTACTTGTTTTTACAGGAAAAATGAGTGCCTA 10380

||||||||||||||||||||||||||||||||||||||||||||||||||||||||||||

TS-165 10351 TCTATCAGTGCCCTGTACCCCACAAATTTTACTTGTTTTTACAGGAAAAATGAGTGCCTA 10410

SG-7 10381 TTTGTGTAGATTTTGGATATGTATACCTTGGTGGTGTGGGTCTGGAGTTGCTCTAATTGT 10440

||||||||||||||||||||||||||||||||||||||||||||||||||||||||||||

TS-165 10411 TTTGTGTAGATTTTGGATATGTATACCTTGGTGGTGTGGGTCTGGAGTTGCTCTAATTGT 10470

SG-7 10441 TCCCCTAAAATTTGAAAACTTGCCCTTTTGTATGATGGGTCTGAATTATGTTTAGCTATT 10500

||||||||||||||||||||||||||||||||||||||||||||||||||||||||||||

TS-165 10471 TCCCCTAAAATTTGAAAACTTGCCCTTTTGTATGATGGGTCTGAATTATGTTTAGCTATT 10530

SG-7 10501 CTTCTTTTAGTATGCTGCACTGTCTTGTGTTTCATGGCATATGTATAATAGTTTGGCTGA 10560

||||||||||||||||||||||||||||||||||||||||||||||||||||||||||||

TS-165 10531 CTTCTTTTAGTATGCTGCACTGTCTTGTGTTTCATGGCATATGTATAATAGTTTGGCTGA 10590

SG-7 10561 TAGCAGCACGTTCTAGTGTTATTTACAGTAGTTCTGCTGTGTGTGTTAGATGTAATTATG 10620

||||||||||||||||||||||||||||||||||||||||||||||||||||||||||||

TS-165 10591 TAGCAGCACGTTCTAGTGTTATTTACAGTAGTTCTGCTGTGTGTGTTAGATGTAATTATG 10650

SG-7 10621 TAAATTAAGGTTATCGTTATGCATATATCATGGCTTTTATCACTTCAAATTGTACAATTT 10680

||||||||||||||||||||||||||||||||||||||||||||||||||||||||||||

TS-165 10651 TAAATTAAGGTTATCGTTATGCATATATCATGGCTTTTATCACTTCAAATTGTACAATTT 10710

SG-7 10681 CTGAAATTTTACCTCTGTTTTCTTATAGAGAGTGGTTCCGCTCAATACATGGATCCTAAT 10740

||||||||||||||||||||||||||||||||||||||||||||||||||||||||||||

TS-165 10711 CTGAAATTTTACCTCTGTTTTCTTATAGAGAGTGGTTCCGCTCAATACATGGATCCTAAT 10770

SG-7 10741 TTCAAACTTCAAGTTGTCTTACAATCTTCTCCGTCGCCCTGATGGGACTTTCAATCGTCA 10800

||||||||||||||||||||||||||||||||||||||||||||||||||||||||||||

TS-165 10771 TTCAAACTTCAAGTTGTCTTACAATCTTCTCCGTCGCCCTGATGGGACTTTCAATCGTCA 10830

SG-7 10801 CTTGGCAGAGTTCCTTGACCGCAAGGTTCCAGCGAATGCAAATCCAGTTGATGGAGTTTT 10860

||||||||||||||||||||||||||||||||||||||||||||||||||||||||||||

TS-165 10831 CTTGGCAGAGTTCCTTGACCGCAAGGTTCCAGCGAATGCAAATCCAGTTGATGGAGTTTT 10890

SG-7 10861 CTCTTTTGATGTTCTCATTGATCGTGAAATAGGCCTACTTAGCCGTGTCTATCGGCCTTC 10920

||||||||||||||||||||||||||||||||||||||||||| ||||||||||||||||

TS-165 10891 CTCTTTTGATGTTCTCATTGATCGTGAAATAGGCCTACTTAGCTGTGTCTATCGGCCTTC 10950

SG-7 10921 TTTTGAGGATGGAGCTTCACCGAATATGGCTGAACTTGAAAAGCCTGTGACTGCTGATGT 10980

||||||||||||||||||||||||||||||||||||||||||||||||||||||||||||

TS-165 10951 TTTTGAGGATGGAGCTTCACCGAATATGGCTGAACTTGAAAAGCCTGTGACTGCTGATGT 11010

SG-7 10981 TGTACCTGTCATAATTTTCTTCCATGGTGGAAGTTTTGCACACTCTTCTTTCAATAGTGC 11040

||||||||||||||||||||||||||||||||||||||||||||||||||||||||||||

TS-165 11011 TGTACCTGTCATAATTTTCTTCCATGGTGGAAGTTTTGCACACTCTTCTTTCAATAGTGC 11070

SG-7 11041 CATCTATGACACACTTTGTCGCCGCCTTGTTGGCATTTGCAAGGCAGTTGTTGTGTCAGT 11100

||||||||||||||||||||||||||||||||||||||||||||||||||||||||||||

TS-165 11071 CATCTATGACACACTTTGTCGCCGCCTTGTTGGCATTTGCAAGGCAGTTGTTGTGTCAGT 11130

SG-7 11101 TAATTACAGGCGAGCTCCTGAAAACCGTTATCCTTGTGCTTATAATGATGGATGGACAGT 11160

||||||||||||||||||||||||||||||||||||||||||||||||||||||||||||

TS-165 11131 TAATTACAGGCGAGCTCCTGAAAACCGTTATCCTTGTGCTTATAATGATGGATGGACAGT 11190

SG-7 11161 TCTTGAGTGGGTTAACTCAAGGGAATGGCTGCGGAGCAAAAAGGACTCGAAGGCTCACAT 11220

||||||||||||||||||||||||||||||||||||||||||||||||||||||||||||

TS-165 11191 TCTTGAGTGGGTTAACTCAAGGGAATGGCTGCGGAGCAAAAAGGACTCGAAGGCTCACAT 11250

SG-7 11221 ATACTTAGCTGGAGATAGCTCTGGTGGTAATATTGTTCATAATGTGGCTTTCAGGGCAGT 11280

||||||||||||||||||||||||||||||||||||||||||||||||||||||||||||

TS-165 11251 ATACTTAGCTGGAGATAGCTCTGGTGGTAATATTGTTCATAATGTGGCTTTCAGGGCAGT 11310

SG-7 11281 AGAATCCAACATAGAAGTGTTGGGAAATATACTGCTGAACCCTATGTTTGGTGGACAAGA 11340

||||||||||||||||||||||||||||||||||||||||||||||||||||||||||||

TS-165 11311 AGAATCCAACATAGAAGTGTTGGGAAATATACTGCTGAACCCTATGTTTGGTGGACAAGA 11370

SG-7 11341 GAGAACAGAATCAGAGAAGCGATTGGATGGCAAATATTTTGTCACACTTCAAGACCGAGA 11400

||||||||||||||||||||||||||||||||||||||||||||||||||||||||||||

TS-165 11371 GAGAACAGAATCAGAGAAGCGATTGGATGGCAAATATTTTGTCACACTTCAAGACCGAGA 11430

SG-7 11401 CTGGTATTGGAGAGCTTATCTTCCTGAAGATTCAGACAGGGACCATCCTGCATGCAACCC 11460

||||||||||||||||||||||||||||||||||||||||||||||||||||||||||||

TS-165 11431 CTGGTATTGGAGAGCTTATCTTCCTGAAGATTCAGACAGGGACCATCCTGCATGCAACCC 11490

SG-7 11461 TTTTGGTCCAAATGGTATAAACCTCAAAGGCGTCAAGTTCCCAAAGAATCTTGTTGTTGT 11520

||||||||||||||||||||||||||||||||||||||||||||||||||||||||||||

TS-165 11491 TTTTGGTCCAAATGGTATAAACCTCAAAGGCGTCAAGTTCCCAAAGAATCTTGTTGTTGT 11550

SG-7 11521 CGCAGGTTTGGACCTTGTTCAGGATTGGCAGTTGGCTTATGCTGATGGGCTTAAGAAGGC 11580

||||||||||||||||||||||||||||||||||||||||||||||||||||||||||||

TS-165 11551 CGCAGGTTTGGACCTTGTTCAGGATTGGCAGTTGGCTTATGCTGATGGGCTTAAGAAGGC 11610

SG-7 11581 TGGACAAGAGGTTAACCTGATATATTTGGAGAAGGCAACAATAGGGTTCTACCTGTTGCC 11640

||||||||||||||||||||||||||||||||||||||||||||||||||||||||||||

TS-165 11611 TGGACAAGAGGTTAACCTGATATATTTGGAGAAGGCAACAATAGGGTTCTACCTGTTGCC 11670

SG-7 11641 AAATAATGAACACTTCTACACTGTCATGGATGAGATAAGTAGCTTCGTGAGTTCTGACTC 11700

||||||||||||||||||||||||||||||||||||||||||||||||||||||||||||

TS-165 11671 AAATAATGAACACTTCTACACTGTCATGGATGAGATAAGTAGCTTCGTGAGTTCTGACTC 11730

SG-7 11701 TCAG**TAG**ATTTAACCTTGTCAAAAGTAGGATATGCTTTGAAGACGTTTGATGTTTTGTTG 11760

||||**|||**|||||||||||||||||||||||||||||||||||||||||||||||||||||

TS-165 11731 TCAG**TAG**ATTTAACCTTGTCAAAAGTAGGATATGCTTTGAAGACGTTTGATGTTTTGTTG 11790

SG-7 11761 AAGTTAGTTCCTAGCCTGTCAACCGTTTGAAAGATTGTATAGCATCATCAATTACTTCCT 11820

||||||||||||||||||||||||||||||||||||||||||||||||||||||||||||

TS-165 11791 AAGTTAGTTCCTAGCCTGTCAACCGTTTGAAAGATTGTATAGCATCATCAATTACTTCCT 11850

SG-7 11821 TATTGATCATGCTATTGCTTGGATTCTGCTCCATTGGCTGGGTTGGTTATTGGTGGCGGA 11880

||||||||||||||||||||||||||||||||||||||||||||||||||||||||||||

TS-165 11851 TATTGATCATGCTATTGCTTGGATTCTGCTCCATTGGCTGGGTTGGTTATTGGTGGCGGA 11910

SG-7 11881 AAACCTCAATCATGTAGCTGGATCTGTGTTATATTTATTCCAGGTACAGGGAGTGTCTGG 11940

||||||||||||||||||||||||||||||||||||||||||||||||||||||||||||

TS-165 11911 AAACCTCAATCATGTAGCTGGATCTGTGTTATATTTATTCCAGGTACAGGGAGTGTCTGG 11970

SG-7 11941 ATATTGGTTGTATATTTTGGTAGCTTGCGCCAAAGGTTATAATCATCTTGTCTTCGTCTA 12000

||||||||||||||||||||||||||||||||||||||||||||||||||||||||||||

TS-165 11971 ATATTGGTTGTATATTTTGGTAGCTTGCGCCAAAGGTTATAATCATCTTGTCTTCGTCTA 12030

SG-7 12001 GCAGATATGCTGATTTACAGCTAACCTTTCCCATGTTAGCCTCCGTAGTAGAGGGGGTAA 12060

||||||||||||||||||||||||||||||||||||||||||||||||||||||||||||

TS-165 12031 GCAGATATGCTGATTTACAGCTAACCTTTCCCATGTTAGCCTCCGTAGTAGAGGGGGTAA 12090

SG-7 12061 GTCTGGTGCTGATCCGTCAGGGGACCAGTGCCTTGCTAATGTTATATAGCTGATCATACT 12120

||||||||||||||||||||||||||||||||||||||||||||||||||||||||||||

TS-165 12091 GTCTGGTGCTGATCCGTCAGGGGACCAGTGCCTTGCTAATGTTATATAGCTGATCATACT 12150

SG-7 12121 ATTATGCTTCACGGATGAACATCCTAAATTGTGAAGTTGTATAATATATCTATAATTATA 12180

||||||||||||||||||||||||||||||||||||||||||||||||||||||||||||

TS-165 12151 ATTATGCTTCACGGATGAACATCCTAAATTGTGAAGTTGTATAATATATCTATAATTATA 12210

SG-7 12181 TAGAACTATGTTTGCTTCCTCGCGTTATCATGGTGTGTCGCCCTTTGGTTTGTTTTCTTT 12240

||||||||||||||||||||||||||||||||||||||||||||||||||||||||||||

TS-165 12211 TAGAACTATGTTTGCTTCCTCGCGTTATCATGGTGTGTCGCCCTTTGGTTTGTTTTCTTT 12270

SG-7 12241 TTTATGTTCTTTGTCACTTCCTTGACCTTCAATATGTACATCCTTAGATCACTTATATTT 12300

||||||||||||||||||||||||||||||||||||||||||||||||||||||||||||

TS-165 12271 TTTATGTTCTTTGTCACTTCCTTGACCTTCAATATGTACATCCTTAGATCACTTATATTT 12330

SG-7 12301 AGAAGATATGTTTGCAAGTACTGTTTCTTTTCTCAGGGATGCACAATATGAACATATGTA 12360

||||||||||||||||||||||||||||||||||||||||||||||||||||||||||||

TS-165 12331 AGAAGATATGTTTGCAAGTACTGTTTCTTTTCTCAGGGATGCACAATATGAACATATGTA 12390

SG-7 12361 ATTCAACATGCTTCAATTGTTATAAGTGGAATTGTTGTTCTCTTGCACATGAACTGATGT 12420

||||||||||||||||||||||||||||||||||||||||||||||||||||||||||||

TS-165 12391 ATTCAACATGCTTCAATTGTTATAAGTGGAATTGTTGTTCTCTTGCACATGAACTGATGT 12450

SG-7 12421 GCTATGAGGTCTTTCTATATTGCAATGTTGGCACTGTCTAATACATGAGGTGACTTTTTT 12480

||||||||||||||||||||||||||||||||||||||||||||||||||||||||||||

TS-165 12451 GCTATGAGGTCTTTCTATATTGCAATGTTGGCACTGTCTAATACATGAGGTGACTTTTTT 12510

SG-7 12481 GTTGGTCAACTGGAAAGTGTTACTTATTATGATTAGTGGAGTGTAGTTCTGAAAAAATTA 12540

||||||||||||||||||||||||||||||||||||||||||||||||||||||||||||

TS-165 12511 GTTGGTCAACTGGAAAGTGTTACTTATTATGATTAGTGGAGTGTAGTTCTGAAAAAATTA 12570

SG-7 12541 TAGTATTTGAATGTAAGTAGCGCGAAGGTGGAACCTATTCATCAATTAAATGAGTGAAAA 12600

||||||||||||||||||||||||||||||||||||||||||||||||||||||||||||

TS-165 12571 TAGTATTTGAATGTAAGTAGCGCGAAGGTGGAACCTATTCATCAATTAAATGAGTGAAAA 12630

SG-7 12601 TCATACTAAACTATATGTTTGGCCACAGATTTTGCCTCAACCAAAAAGATCTCAAAGAAG 12660

||||||||||||||||||||||||||||||||||||||||||||||||||||||||||||

TS-165 12631 TCATACTAAACTATATGTTTGGCCACAGATTTTGCCTCAACCAAAAAGATCTCAAAGAAG 12690

SG-7 12661 TGTTTGTCTATCAAATTAAACCATTATTTCAAAATATCGTTGGACAGGTTCAAGTCGAGG 12720

||||||||||||||||||||||||||||||||||||||||||||||||||||||||||||

TS-165 12691 TGTTTGTCTATCAAATTAAACCATTATTTCAAAATATCGTTGGACAGGTTCAAGTCGAGG 12750

SG-7 12721 TTTGTGTAAGCTGATATGTCCTCAACAGaaaaaagaaaaagaaaaaaaaGAAGCTTAAAT 12780

||||||||||||||||||||||||||||||||||||||||||||||||||||||||||||

TS-165 12751 TTTGTGTAAGCTGATATGTCCTCAACAGAAAAAAGAAAAAGAAAAAAAAGAAGCTTAAAT 12810

SG-7 12781 ACATGTGTAGAAGCAGTGGTGATTTGAGAATAGAGACAAAATAAGCTGTCTGTCTTTTGG 12840

||||||||||||||||||||||||||||||||||||||||||||||||||||||||||||

TS-165 12811 ACATGTGTAGAAGCAGTGGTGATTTGAGAATAGAGACAAAATAAGCTGTCTGTCTTTTGG 12870

SG-7 12841 TGACCTTTATGAATCTAGTCCTATAGTGTCAAACTAGTGAAGAACTCACTGGCAAAACTG 12900

||||||||||||||||||||||||||||||||||||||||||||||||||||||||||||

TS-165 12871 TGACCTTTATGAATCTAGTCCTATAGTGTCAAACTAGTGAAGAACTCACTGGCAAAACTG 12930

SG-7 12901 CTTCTTTGCTCCCTTCCCCTTTCTATTTAGTTTAAATTTAAATAAATTATATATGGCTCA 12960

||||||||||||||||||||||||||||||||||||||||||||||||||||||||||||

TS-165 12931 CTTCTTTGCTCCCTTCCCCTTTCTATTTAGTTTAAATTTAAATAAATTATATATGGCTCA 12990

SG-7 12961 AGATAGTGGCGTTTTCTTTTATATAATTTGAGTAAAACAAAAAGATTTTGCAAATATGTC 13020

||||||||||||||||||||||||||||||||||||||||||||||||||||||||||||

TS-165 12991 AGATAGTGGCGTTTTCTTTTATATAATTTGAGTAAAACAAAAAGATTTTGCAAATATGTC 13050

SG-7 13021 ATGTTTAAAAAATGCTTAAGAGGGATTTAACaaaaaaaaaacaaaaaCGAATTGACTGGT 13080

||||||||||||||||||||||||||||||||||||||||||||||||||||||||||||

TS-165 13051 ATGTTTAAAAAATGCTTAAGAGGGATTTAACAAAAAAAAAACAAAAACGAATTGACTGGT 13110

SG-7 13081 CACAGACATAGATTTTGGAAATATGGATACAGATATTGACTTGTTAGGAGAAAGAGTCCA 13140

||||||||||||||||||||||||||||||||||||||||||||||||||||||||||||

TS-165 13111 CACAGACATAGATTTTGGAAATATGGATACAGATATTGACTTGTTAGGAGAAAGAGTCCA 13170

SG-7 13141 GTTGGCGGTGATTGCTATTTGGATGTATTGAAGGGGACCATTTTTCAAAACCAACAAAAT 13200

||||||||||||||||||||||||||||||||||||||||||||||||||||||||||||

TS-165 13171 GTTGGCGGTGATTGCTATTTGGATGTATTGAAGGGGACCATTTTTCAAAACCAACAAAAT 13230

SG-7 13201 CAATCAGGTGAACttttgttttttcttttattttCTTAGAGACCAAAGTGGGTGAGCTCA 13260

||||||||||||||||||||||||||||||||||||||||||||||||||||||||||||

TS-165 13231 CAATCAGGTGAACTTTTGTTTTTTCTTTTATTTTCTTAGAGACCAAAGTGGGTGAGCTCA 13290

SG-7 13261 CTATTTCTTTAAGaaaaaaaaaaaCGTGAATCTGTACGCGAATCTTACTCTTATCTTAGT 13320

||||||||||||||||||||||||||||||||||||||||||||||||||||||||||||

TS-165 13291 CTATTTCTTTAAGAAAAAAAAAAACGTGAATCTGTACGCGAATCTTACTCTTATCTTAGT 13350

SG-7 13321 GAAGCTAGAGAGGTGATTAAGAAAAATCATGCATCAAAACTCGTATTTGTGTCTAGTCAA 13380

||||||||||||||||||||||||||||||||||||||||||||||||||||||||||||

TS-165 13351 GAAGCTAGAGAGGTGATTAAGAAAAATCATGCATCAAAACTCGTATTTGTGTCTAGTCAA 13410

SG-7 13381 ACAATGTCATAACACATAATACTCATATTTGTTTTGTCTATTTTTGTGGGTGTCATTTCA 13440

||||||||||||||||||||||||||||||||||||||||||||||||||||||||||||

TS-165 13411 ACAATGTCATAACACATAATACTCATATTTGTTTTGTCTATTTTTGTGGGTGTCATTTCA 13470

SG-7 13441 TGAGGTAAATGTAAATGAATGAAAATTACAGTTTGTAAGAGTGGAAGTCATTGTCATATG 13500

||||||||||||||||||||||||||||||||||||||||||||||||||||||||||||

TS-165 13471 TGAGGTAAATGTAAATGAATGAAAATTACAGTTTGTAAGAGTGGAAGTCATTGTCATATG 13530

SG-7 13501 GAAGAGAAAAGGTGTCTAATCATGGATAAGGGAATTCGAGTGCTTTTGCGTtaaaataaa 13560

||||||||||||||||||||||||||||||||||||||||||||||||||||||||||||

TS-165 13531 GAAGAGAAAAGGTGTCTAATCATGGATAAGGGAATTCGAGTGCTTTTGCGTTAAAATAAA 13590

SG-7 13561 gggaataaataatattctgactatagaaaaatgtttgaaattagaaattgaaaaacagaa 13620

||||||||||||||||||||||||||||||||||||||||||||||||||||||||||||

TS-165 13591 GGGAATAAATAATATTCTGACTATAGAAAAATGTTTGAAATTAGAAATTGAAAAACAGAA 13650

SG-7 13621 taatattactataaaaataaaaaaaGAACAATATTTGCTACTTCATGAATCATGAACAAT 13680

||||||||||||||||||||||||||||||||||||||||||||||||||||||||||||

TS-165 13651 TAATATTACTATAAAAATAAAAAAAGAACAATATTTGCTACTTCATGAATCATGAACAAT 13710

SG-7 13681 AATATATATAACATATAGTATCAAGTTCATTTGATTGAATTGTACAATTTGCCCTTCAAA 13740

||||||||||||||||||||||||||||||||||||||||||||||||||||||||||||

TS-165 13711 AATATATATAACATATAGTATCAAGTTCATTTGATTGAATTGTACAATTTGCCCTTCAAA 13770

SG-7 13741 TTGGTTGATGTTTAATTCTTGCCCTTAGCAATCGAGACATAAGTTCTTTAAAAGCGCGCA 13800

||||||||||||||||||||||||||||||||||||||||||||||||||||||||||||

TS-165 13771 TTGGTTGATGTTTAATTCTTGCCCTTAGCAATCGAGACATAAGTTCTTTAAAAGCGCGCA 13830

SG-7 13801 ATAGAACAAGTGGGGAATGACTTTACAAAATCGTGACTATTTTTCAATTATAGACACGTA 13860

||||||||||||||||||||||||||||||||||||||||||||||||||||||||||||

TS-165 13831 ATAGAACAAGTGGGGAATGACTTTACAAAATCGTGACTATTTTTCAATTATAGACACGTA 13890

SG-7 13861 TCGTATTTTTACCCTTTTTCTGGAATGTGGAGTATTTGGGCTGTGAAGATGTGGCCCAAG 13920

||||||||||||||||||||||||||||||||||||||||||||||||||||||||||||

TS-165 13891 TCGTATTTTTACCCTTTTTCTGGAATGTGGAGTATTTGGGCTGTGAAGATGTGGCCCAAG 13950

SG-7 13921 TATCCTCCACTACATGGTCCTAACTAGATCTAGGCCCAATTCAAAACGCATGAATAAAGG 13980

||||||||||||||||||||||||||||||||||||||||||||||||||||||||||||

TS-165 13951 TATCCTCCACTACATGGTCCTAACTAGATCTAGGCCCAATTCAAAACGCATGAATAAAGG 14010

SG-7 13981 AGAAAATTGTGtatatataatcaatgaaaaataaatcaatttataatgaattttcataat 14040

||||||||||||||||||||||||||||||||||||||||||||||||||||||||||||

TS-165 14011 AGAAAATTGTGTATATATAATCAATGAAAAATAAATCAATTTATAATGAATTTTCATAAT 14070

SG-7 14041 acatattaatataCTCATATATCACATGTTATACCAACATGCATGCAGGTATGACGAAAA 14100

||||||||||||||||||||||||||||||||||||||||||||||||||||||||||||

TS-165 14071 ACATATTAATATACTCATATATCACATGTTATACCAACATGCATGCAGGTATGACGAAAA 14130

SG-7 14101 TGACAAAAGGGTTCCATCAATATTCACACTTATATGTAtaaaaaataaataaataagata 14160

||||||||||||||||||||||||||||||||||||||||||||||||||||||||||||

TS-165 14131 TGACAAAAGGGTTCCATCAATATTCACACTTATATGTATAAAAAATAAATAAATAAGATA 14190

SG-7 14161 atttcactaacatcaatgttcagatgtatacaaaataaaaaaaaaGGCCAACAAAGTCAT 14220

||||||||||||||||||||||||||||||||||||||||||||||||||||||||||||

TS-165 14191 ATTTCACTAACATCAATGTTCAGATGTATACAAAATAAAAAAAAAGGCCAACAAAGTCAT 14250

SG-7 14221 CATTGTTTTGTTATTATTGTTGTTGTTATCGTTCTCCCAGAAAATTATACTAATAGTATA 14280

||||||||||||||||||||||||||||||||||||||||||||||||||||||||||||

TS-165 14251 CATTGTTTTGTTATTATTGTTGTTGTTATCGTTCTCCCAGAAAATTATACTAATAGTATA 14310

SG-7 14281 ATAGGTAATGGATCACATGCAATTAATATTAGAAAATATATTGTTTGGCTGTACTTTGCA 14340

||||||||||||||||||||||||||||||||||||||||||||||||||||||||||||

TS-165 14311 ATAGGTAATGGATCACATGCAATTAATATTAGAAAATATATTGTTTGGCTGTACTTTGCA 14370

SG-7 14341 CACACACCTAATAGTAGAGTAAGAAATCATCAATAGCCAAATCATAACTTAGTTGACCTT 14400

||||||||||||||||||||||||||||||||||||||||||||||||||||||||||||

TS-165 14371 CACACACCTAATAGTAGAGTAAGAAATCATCAATAGCCAAATCATAACTTAGTTGACCTT 14430

SG-7 14401 CAATTTTGAAACCACATATGTTCAATGAACCCACAAATAAATAAATAAATAAATATCAAC 14460

||||||||||||||||||||||||||||||||||||||||||||||||||||||||||||

TS-165 14431 CAATTTTGAAACCACATATGTTCAATGAACCCACAAATAAATAAATAAATAAATATCAAC 14490

SG-7 14461 TTTATTTATTTACTTTTAAATAATTTGGAGTAGCAAAATTCAAAGATAAAGAAAAAGAAA 14520

||||||||||||||||||||||||||||||||||||||||||||||||||||||||||||

TS-165 14491 TTTATTTATTTACTTTTAAATAATTTGGAGTAGCAAAATTCAAAGATAAAGAAAAAGAAA 14550

SG-7 14521 GTAGTGTTTCCATGAGTACAAGTGGTCCTAAGAAAAAGACAACATAACTCATAATCGTAA 14580

||||||||||||||||||||||||||||||||||||||||||||||||||||||||||||

TS-165 14551 GTAGTGTTTCCATGAGTACAAGTGGTCCTAAGAAAAAGACAACATAACTCATAATCGTAA 14610

SG-7 14581 AGTAGAAGAATTAAATTTTATAGTAATGCCAAGGAGCAAGGACGGTGCATCAAATCGAGA 14640

||||||||||||||||||||||||||||||||||||||||||||||||||||||||||||

TS-165 14611 AGTAGAAGAATTAAATTTTATAGTAATGCCAAGGAGCAAGGACGGTGCATCAAATCGAGA 14670

SG-7 14641 ATATATCTAATTTATAGCTTGATTTCTCACATTTTAACTGTAATTTTAGCTTAAAAAACT 14700

||||||||||||||||||||||||||||||||||||||||||||||||||||||||||||

TS-165 14671 ATATATCTAATTTATAGCTTGATTTCTCACATTTTAACTGTAATTTTAGCTTAAAAAACT 14730

SG-7 14701 TATTTATAAGTTGATAGTTGTTTAATTATATGCTTAGCACTACAGATTTACAGACAATAT 14760

||||||||||||||||||||||||||||||||||||||||||||||||||||||||||||

TS-165 14731 TATTTATAAGTTGATAGTTGTTTAATTATATGCTTAGCACTACAGATTTACAGACAATAT 14790

SG-7 14761 TTAAAGAAGATAAAATAATCTCTCTTTAGACTTTAATAAATAAGATTACTGAAGACATAT 14820

||||||||||||||||||||||||||||||||||||||||||||||||||||||||||||

TS-165 14791 TTAAAGAAGATAAAATAATCTCTCTTTAGACTTTAATAAATAAGATTACTGAAGACATAT 14850

SG-7 14821 ACTATATCTTATATTTGTCTTCGAACTTGGTTAAGGATATGGTGCACACAATTTTATATT 14880

||||||||||||||||||||||||||||||||||||||||||||||||||||||||||||

TS-165 14851 ACTATATCTTATATTTGTCTTCGAACTTGGTTAAGGATATGGTGCACACAATTTTATATT 14910

SG-7 14881 TTGTGTTTAACTAtttttttAGAGATGCGGTTTGTTTCTTGATAGAATAATTCGAAAGAT 14940

||||||||||||||||||||||||||||||||||||||||||||||||||||||||||||

TS-165 14911 TTGTGTTTAACTATTTTTTTAGAGATGCGGTTTGTTTCTTGATAGAATAATTCGAAAGAT 14970

SG-7 14941 TATTATACATCTATTTTAATTTGACATCTCTAACATTTGAAACTATTCAAAAATTTTAGT 15000

||||||||||||||||||||||||||||||||||||||||||||||||||||||||||||

TS-165 14971 TATTATACATCTATTTTAATTTGACATCTCTAACATTTGAAACTATTCAAAAATTTTAGT 15030

SG-7 15001 GACATCTTAATTCGATATTAAATGTACATTGATCATGCATATAATCAAGCATTATTAAGT 15060

||||||||||||||||||||||||||||||||||||||||||||||||||||||||||||

TS-165 15031 GACATCTTAATTCGATATTAAATGTACATTGATCATGCATATAATCAAGCATTATTAAGT 15090

SG-7 15061 AGAGATTTAACTTATTTACAATAATTCTAACAAAATTAAAGATGATAGCTACACATAAAC 15120

||||||||||||||||||||||||||||||||||||||||||||||||||||||||||||

TS-165 15091 AGAGATTTAACTTATTTACAATAATTCTAACAAAATTAAAGATGATAGCTACACATAAAC 15150

SG-7 15121 TACATTAATGAAGTCCTTCTCATCTTCACTTTCATTGCTTATACATTTGAAGCCAATATC 15180

||||||||||||||||||||||||||||||||||||||||||||||||||||||||||||

TS-165 15151 TACATTAATGAAGTCCTTCTCATCTTCACTTTCATTGCTTATACATTTGAAGCCAATATC 15210

SG-7 15181 ATGAACACAATCTAAATTAACATATCCAAATAACTTCTAATTAAAGTTGCCATTAATTAA 15240

||||||||||||||||||||||||||||||||||||||||||||||||||||||||||||

TS-165 15211 ATGAACACAATCTAAATTAACATATCCAAATAACTTCTAATTAAAGTTGCCATTAATTAA 15270

SG-7 15241 ACATAATTAAATTCCATATATCTCATCAATCAATATTCACACCACTGGAAATGATCCTCC 15300

||||||||||||||||||||||||||||||||||||||||||||||||||||||||||||

TS-165 15271 ACATAATTAAATTCCATATATCTCATCAATCAATATTCACACCACTGGAAATGATCCTCC 15330

SG-7 15301 GGCATCCACGAATTGTTTCTCCAGCTACAAATTAATCAATTAATTGCAAAAATGAGTCCA 15360

||||||||||||||||||||||||||||||||||||||||||||||||||||||||||||

TS-165 15331 GGCATCCACGAATTGTTTCTCCAGCTACAAATTAATCAATTAATTGCAAAAATGAGTCCA 15390

SG-7 15361 AAACACCTCTTCCTCACTTGTTATACAATTAATTAATCATATATAGATGAAAAATGAAAA 15420

||||||||||||||||||||||||||||||||||||||||||||||||||||||||||||

TS-165 15391 AAACACCTCTTCCTCACTTGTTATACAATTAATTAATCATATATAGATGAAAAATGAAAA 15450

SG-7 15421 TCACCTTAAATAAAGTTTCAAGCTTGTTCTTGACAAGGGTGTACTCAATTTTCACTTGTT 15480

||||||||||||||||||||||||||||||||||||||||||||||||||||||||||||

TS-165 15451 TCACCTTAAATAAAGTTTCAAGCTTGTTCTTGACAAGGGTGTACTCAATTTTCACTTGTT 15510

SG-7 15481 GCAAACATTTTAAGCACCTGCATTCAAATGATACTATTTAGCTTTATTAATTTCAGTAAC 15540

||||||||||||||||||||||||||||||||||||||||||||||||||||||||||||

TS-165 15511 GCAAACATTTTAAGCACCTGCATTCAAATGATACTATTTAGCTTTATTAATTTCAGTAAC 15570

SG-7 15541 AACGTATCTAATACAATTTCATAAATTGAGTCCGCAGAGTGCGATATAAACAGATTTTAC 15600

||||||||||||||||||||||||||||||||||||||||||||||||||||||||||||

TS-165 15571 AACGTATCTAATACAATTTCATAAATTGAGTCCGCAGAGTGCGATATAAACAGATTTTAC 15630

SG-7 15601 TTTTAGATTATATaaaaaattgaaacgaaaacaccaaaaaaaaaaaTTAATTTAAAACCT 15660

||||||||||||||||||||||||||||||||||||||||||||||||||||||||||||

TS-165 15631 TTTTAGATTATATAAAAAATTGAAACGAAAACACCAAAAAAAAAAATTAATTTAAAACCT 15690

SG-7 15661 ACCCTTCAACATTGCGTTCATCACAATAATTACGGAAGGAAATGCACACTCTTTGAACTC 15720

||||||||||||||||||||||||||||||||||||||||||||||||||||||||||||

TS-165 15691 ACCCTTCAACATTGCGTTCATCACAATAATTACGGAAGGAAATGCACACTCTTTGAACTC 15750

SG-7 15721 TTTGTGCCCCAATGCTGTAAATTAAAAATAAGAGTTTAAATTATGTACCCTCAACTAATT 15780

||||||||||||||||||||||||||||||||||||||||||||||||||||||||||||

TS-165 15751 TTTGTGCCCCAATGCTGTAAATTAAAAATAAGAGTTTAAATTATGTACCCTCAACTAATT 15810

SG-7 15781 AGTTAATATATaaaaaaaTTATATTATCAAATTTTATCTAATAGATAATTAGAGATACCG 15840

||||||||||||||||||||||||||||||||||||||||||||||||||||||||||||

TS-165 15811 AGTTAATATATAAAAAAATTATATTATCAAATTTTATCTAATAGATAATTAGAGATACCG 15870

SG-7 15841 TGAGTTAAAATAGGTTTAATAATAGTaaaaaaaaaaaaagaaaatcttaatatacaaaaa 15900

||||||||||||||||||||||||||||||| ||||||||||||||||||||||||||||

TS-165 15871 TGAGTTAAAATAGGTTTAATAATAGTAAAAAGAAAAAAAGAAAATCTTAATATACAAAAA 15930

SG-7 15901 aaaaTATTTACCTAGAGCTGCTACCTTTCAGCTGATGAACATGAGCATCCAGTTTCTTGA 15960

||||||||||||||||||||||||||||||||||||||||||||||||||||||||||||

TS-165 15931 AAAATATTTACCTAGAGCTGCTACCTTTCAGCTGATGAACATGAGCATCCAGTTTCTTGA 15990

SG-7 15961 AGTCTACATTAGACTGATTGCTATTTTTACAAACAAAAATTGAAATATTATGTGAGCTTG 16020

||||||||||||||||||||||||||||||||||||||||||||||||||||||||||||

TS-165 15991 AGTCTACATTAGACTGATTGCTATTTTTACAAACAAAAATTGAAATATTATGTGAGCTTG 16050

SG-7 16021 CTTATATTGTCGATCTTAAATACAAATAAATGCGAAAGCTTACATCTAGCGTAAAACTTG 16080

||||||||||||||||||||||||||||||||||||||||||||||||||||||||||||

TS-165 16051 CTTATATTGTCGATCTTAAATACAAATAAATGCGAAAGCTTACATCTAGCGTAAAACTTG 16110

SG-7 16081 TCAGTTCATAATAAAACAGAGATAATAAGTTCATAAATATGTTTTTCCACCTACATCTCT 16140

||||||||||||||||||||||||||||||||||||||||||||||||||||||||||||

TS-165 16111 TCAGTTCATAATAAAACAGAGATAATAAGTTCATAAATATGTTTTTCCACCTACATCTCT 16170

SG-7 16141 TCCAGATTTGTATGTAAAAAGGGGAAATTAAACAAATTAATTACTCACAGAGCTTTTGCA 16200

||||||||||||||||||||||||||||||||||||||||||||||||||||||||||||

TS-165 16171 TCCAGATTTGTATGTAAAAAGGGGAAATTAAACAAATTAATTACTCACAGAGCTTTTGCA 16230

SG-7 16201 AGTTCATTAAGAAGCCTTTCAGAATCTTCAAAGAAAAGGGATACAACTTCCACCACAAAA 16260

||||||||||||||||||||||||||||||||||||||||||||||||||||||||||||

TS-165 16231 AGTTCATTAAGAAGCCTTTCAGAATCTTCAAAGAAAAGGGATACAACTTCCACCACAAAA 16290

SG-7 16261 TCAGGATTGCTCTCATCTTGCAGTTGCTGAAGTTGTATAAACTGTTCATCCAGAATTTTC 16320

||||||||||||||||||||||||||||||||||||||||||||||||||||||||||||

TS-165 16291 TCAGGATTGCTCTCATCTTGCAGTTGCTGAAGTTGTATAAACTGTTCATCCAGAATTTTC 16350

SG-7 16321 TTCTCACACCCaaaaaattaaaaaaaaaaaatcaacaaatcataataaaacaaaaaatTC 16380

||||||||||||||||||||||||||||||||||||||||||||||||||||||||||||

TS-165 16351 TTCTCACACCCAAAAAATTAAAAAAAAAAAATCAACAAATCATAATAAAACAAAAAATTC 16410

SG-7 16381 AATGCACAGAACATTTCGCACATTGTTTTTTGAAAAGGGAAGTAGTTAAAACAATTTATT 16440

||||||||||||||||||||||||||||||||||||||||||||||||||||||||||||

TS-165 16411 AATGCACAGAACATTTCGCACATTGTTTTTTGAAAAGGGAAGTAGTTAAAACAATTTATT 16470

SG-7 16441 TTACTGTTCGAACTCGTAACCTATAGTTCACTCAATCATATGATAAAAAGTTTTATTTAC 16500

||||||||||||||||||||||||||||||||||||||||||||||||||||||||||||

TS-165 16471 TTACTGTTCGAACTCGTAACCTATAGTTCACTCAATCATATGATAAAAAGTTTTATTTAC 16530

SG-7 16501 AAATTTAGAGGTTAAGATTATTTTACCTCATTGTACAAGGAAGCAGTGTACTCAGCTAGT 16560

||||||||||||||||||||||||||||||||||||||||||||||||||||||||||||

TS-165 16531 AAATTTAGAGGTTAAGATTATTTTACCTCATTGTACAAGGAAGCAGTGTACTCAGCTAGT 16590

SG-7 16561 TTTCTTTGGATTTGTTCCATGATTTTGCTAAATTCAAGAGTGACTTTCACAAATTAACAG 16620

||||||||||||||||||||||||||||||||||||||||||||||||||||||||||||

TS-165 16591 TTTCTTTGGATTTGTTCCATGATTTTGCTAAATTCAAGAGTGACTTTCACAAATTAACAG 16650

SG-7 16621 CTTGCTATGCTATTAATGAAAATTGAGAAGTTATATATACTCCTTATTTGTGCATGGAGT 16680

||||||||||||||||||||||||||||||||||||||||||||||||||||||||||||

TS-165 16651 CTTGCTATGCTATTAATGAAAATTGAGAAGTTATATATACTCCTTATTTGTGCATGGAGT 16710

SG-7 16681 AAAtttttttttCCTTGGAAAAGCTACAGCAGGGGCACTCTTTTCTATAACAGAGAAGAC 16740

||||||||||||||||||||||||||||||||||||||||||||||||||||||||||||

TS-165 16711 AAATTTTTTTTTCCTTGGAAAAGCTACAGCAGGGGCACTCTTTTCTATAACAGAGAAGAC 16770

SG-7 16741 TAATATTCAATGAATTTTGCGCATTGATTCACTGTATTTATATTGTCACACTTTATTTTA 16800

||||||||||||||||||||||||||||||||||||||||||||||||||||||||||||

TS-165 16771 TAATATTCAATGAATTTTGCGCATTGATTCACTGTATTTATATTGTCACACTTTATTTTA 16830

SG-7 16801 GTAAttttttttCACCCCCTCACTTTCTGCAATTGTGCTTTTGTTCCTTGTGCATCCTAA 16860

||||||||||||||||||||||||||||||||||||||||||||||||||||||||||||

TS-165 16831 GTAATTTTTTTTCACCCCCTCACTTTCTGCAATTGTGCTTTTGTTCCTTGTGCATCCTAA 16890

SG-7 16861 ATGTCTCCCCTTATTGTTGtttttttGGCAGTTATCTATTTCATTAAAAATATAGTAAAA 16920

||||||||||||||||||||||||||||||||||||||||||||||||||||||||||||

TS-165 16891 ATGTCTCCCCTTATTGTTGTTTTTTTGGCAGTTATCTATTTCATTAAAAATATAGTAAAA 16950

SG-7 16921 GCTGTCACAAGATCCAAAAtattttttaccataccctatttatttgtttcttctttagtc 16980

||||||||||||||||||||||||||||||||||||||||||||||||||||||||||||

TS-165 16951 GCTGTCACAAGATCCAAAATATTTTTTACCATACCCTATTTATTTGTTTCTTCTTTAGTC 17010

SG-7 16981 ctttttgttttatACTAATTCAATCTCGTATTTCACAGCTTAACTTATTTGAAGTTGAAA 17040

||||||||||||||||||||||||||||||||||||||||||||||||||||||||||||

TS-165 17011 CTTTTTGTTTTATACTAATTCAATCTCGTATTTCACAGCTTAACTTATTTGAAGTTGAAA 17070

SG-7 17041 TTaaaaaaaaaaTCGAGAGTACGTCAAGTGAAACATAGTGAGATAATTTAATATTATTAA 17100

||||||||||||||||||||||||||||||||||||||||||||||||||||||||||||

TS-165 17071 TTAAAAAAAAAATCGAGAGTACGTCAAGTGAAACATAGTGAGATAATTTAATATTATTAA 17130

SG-7 17101 GTGAAAATCTACAATCAAATACAAatttcagattttttattcttttttctatttcaattt 17160

||||||||||||||||||||||||||||||||||||||||||||||||||||||||||||

TS-165 17131 GTGAAAATCTACAATCAAATACAAATTTCAGATTTTTTATTCTTTTTTCTATTTCAATTT 17190

SG-7 17161 tAAGAGGGTTGATCCTGTTTGTTATTAATTTCTTTATAATTTTAATTAACTATCGAACAA 17220

||||||||||||||||||||||||||||||||||||||||||||||||||||||||||||

TS-165 17191 TAAGAGGGTTGATCCTGTTTGTTATTAATTTCTTTATAATTTTAATTAACTATCGAACAA 17250

SG-7 17221 CTATCGAACAACTTCTATGTATTTATTTATGTGTATGTTTAGTATTGCTCGATTTGAGAC 17280

||||||||||||||||||||||||||||||||||||||||||||||||||||||||||||

TS-165 17251 CTATCGAACAACTTCTATGTATTTATTTATGTGTATGTTTAGTATTGCTCGATTTGAGAC 17310

SG-7 17281 TAATGAGAACGTCTTATATGTACCATCCAAAATTATGGTGAAATAGACAGAATCCCATTT 17340

||||||||||||||||||||||||||||||||||||||||||||||||||||||||||||

TS-165 17311 TAATGAGAACGTCTTATATGTACCATCCAAAATTATGGTGAAATAGACAGAATCCCATTT 17370

SG-7 17341 ATAACTATCTTTTTTGATGAATCTGAACAATTTGTAAAACATGTACATAGTAAACTATGG 17400

||||||||||||||||||||||||||||||||||||||||||||||||||||||||||||

TS-165 17371 ATAACTATCTTTTTTGATGAATCTGAACAATTTGTAAAACATGTACATAGTAAACTATGG 17430

SG-7 17401 ATGAATAAATTATTGAAATATGATAAAAGGAAAACTTATTTATTACATATGAAAGTGTAG 17460

||||||||||||||||||||||||||||||||||||||||||||||||||||||||||||

TS-165 17431 ATGAATAAATTATTGAAATATGATAAAAGGAAAACTTATTTATTACATATGAAAGTGTAG 17490

SG-7 17461 CAGAATCCAATGTTGCACTGCAAATTGGAATATGTTTAGTTTTTAAGATGGAAAGGTTGC 17520

||||||||||||||||||||||||||||||||||||||||||||||||||||||||||||

TS-165 17491 CAGAATCCAATGTTGCACTGCAAATTGGAATATGTTTAGTTTTTAAGATGGAAAGGTTGC 17550

SG-7 17521 AAGTATGGGCAGGTCAGTATAAGGGGTTGGGGACAGAGAGGTGAAGCTGACCACCCATGT 17580

||||||||||||||||||||||||||||||||||||||||||||||||||||||||||||

TS-165 17551 AAGTATGGGCAGGTCAGTATAAGGGGTTGGGGACAGAGAGGTGAAGCTGACCACCCATGT 17610

SG-7 17581 TACATCAATATCTCCAAGATTTAGTTGGTAGATTCTCTCTGCATATTCTCAACTACTATC 17640

||||||||||||||||||||||||||||||||||||||||||||||||||||||||||||

TS-165 17611 TACATCAATATCTCCAAGATTTAGTTGGTAGATTCTCTCTGCATATTCTCAACTACTATC 17670

SG-7 17641 AATTCTTCGATATTTGaaatttactgatttgattaataaaaatatattatattaaattta 17700

||||||||||||||||||||||||||||||||||||||||||||||||||||||||||||

TS-165 17671 AATTCTTCGATATTTGAAATTTACTGATTTGATTAATAAAAATATATTATATTAAATTTA 17730

SG-7 17701 tttacattcaaataaaaaatttatgatttatGATCCTAATCTGCGTCATTTGAAATAATA 17760

||||||||||||||||||||||||||||||||||||||||||||||||||||||||||||

TS-165 17731 TTTACATTCAAATAAAAAATTTATGATTTATGATCCTAATCTGCGTCATTTGAAATAATA 17790

SG-7 17761 TACTATATTGTGATGATATTGAAAGTTTAAAGTAGCGTGAAATAGCGTACTGTGACAACA 17820

||||||||||||||||||||||||||||||||||||||||||||||||||||||||||||

TS-165 17791 TACTATATTGTGATGATATTGAAAGTTTAAAGTAGCGTGAAATAGCGTACTGTGACAACA 17850

SG-7 17821 AATGTGTCGTGTTTGACATGTGAAAGTGAAAGCCAAATGAAACCATCTTATCTTGGGATC 17880

||||||||||||||||||||||||||||||||||||||||||||||||||||||||||||

TS-165 17851 AATGTGTCGTGTTTGACATGTGAAAGTGAAAGCCAAATGAAACCATCTTATCTTGGGATC 17910

SG-7 17881 AACTTGTTTCTAGCTAGGTAAAAGTTAAACAACAATCTTATCTAAATCCAAATGAAATAT 17940

||||||||||||||||||||||||||||||||||||||||||||||||||||||||||||

TS-165 17911 AACTTGTTTCTAGCTAGGTAAAAGTTAAACAACAATCTTATCTAAATCCAAATGAAATAT 17970

SG-7 17941 CCCTTACCTACTCttttcttttttgttcttttttAATTATTAATATTCCCTCCGTCCAAT 18000

||||||||||||||||||||||||||||||||||||||||||||||||||||||||||||

TS-165 17971 CCCTTACCTACTCTTTTCTTTTTTGTTCTTTTTTAATTATTAATATTCCCTCCGTCCAAT 18030

SG-7 18001 ATTATTTATCATGTTACGCTTTTTAAAAGTTAATTTGATTAATTTTCACGTTAAATTAGA 18060

||||||||||||||||||||||||||||||||||||||||||||||||||||||||||||

TS-165 18031 ATTATTTATCATGTTACGCTTTTTAAAAGTTAATTTGATTAATTTTCACGTTAAATTAGA 18090

SG-7 18061 TTGCATTAATTCGATATTTTAGATaaaaaaaaaTAGACATTATATGAAAAATACTATAAA 18120

||||||||||||||||||||||||||||||||||||||||||||||||||||||||||||

TS-165 18091 TTGCATTAATTCGATATTTTAGATAAAAAAAAATAGACATTATATGAAAAATACTATAAA 18150

SG-7 18121 TTACAAATTTTTTACATGTTAATATGATaaaaaaaTACATCTTAAAATATTAATTAAAGT 18180

||||||||||||||||||||||||||||||||||||||||||||||||||||||||||||

TS-165 18151 TTACAAATTTTTTACATGTTAATATGATAAAAAAATACATCTTAAAATATTAATTAAAGT 18210

SG-7 18181 TTTTATAATTTCACTCTAAAAATGAAAATCATGACAAACAATATCAGACGGATAGAATAC 18240

||||||||||||||||||||||||||||||||||||||||||||||||||||||||||||

TS-165 18211 TTTTATAATTTCACTCTAAAAATGAAAATCATGACAAACAATATCAGACGGATAGAATAC 18270

SG-7 18241 TAAATAGAGGTGGTGATATAACTATATTAACCTTTGATATATTTCCTGGGCGCAATTTTA 18300

||||||||||||||||||||||||||||||||||||||||||||||||||||||||||||

TS-165 18271 TAAATAGAGGTGGTGATATAACTATATTAACCTTTGATATATTTCCTGGGCGCAATTTTA 18330

SG-7 18301 CTTATTCAATATTGATCCAGCACATTCTCTGAGGAGTAACAAATAGTAGAATGGCAATTG 18360

||||||||||||||||||||||||||||||||||||||||||||||||||||||||||||

TS-165 18331 CTTATTCAATATTGATCCAGCACATTCTCTGAGGAGTAACAAATAGTAGAATGGCAATTG 18390

SG-7 18361 TATTAACTAATGTTTGAGTAGAAAAAGATCAATATATTAAGAAAATAGTATAATTAAGGG 18420

||||||||||||||||||||||||||||||||||||||||||||||||||||||||||||

TS-165 18391 TATTAACTAATGTTTGAGTAGAAAAAGATCAATATATTAAGAAAATAGTATAATTAAGGG 18450

SG-7 18421 ATAATGCATAAGTATCCCCTCGACCTATACCCGAAATCTCAGAGACACACTTATATAATA 18480

||||||||||||||||||||||||||||||||||||||||||||||||||||||||||||

TS-165 18451 ATAATGCATAAGTATCCCCTCGACCTATACCCGAAATCTCAGAGACACACTTATATAATA 18510

SG-7 18481 CTAACGTCCTATTACCCTCCTGAACTTATTTTATTAATATTTTTCTACCCCTTTTCGACT 18540

||||||||||||||||||||||||||||||||||||||||||||||||||||||||||||

TS-165 18511 CTAACGTCCTATTACCCTCCTGAACTTATTTTATTAATATTTTTCTACCCCTTTTCGACT 18570

SG-7 18541 TACATGGCACTATCTTGTGGGTCCAATGCTAGTTGACttttttttCAAGTTAGTGCCACG 18600

||||||||||||||||||||||||||||||||||||||||||||||||||||||||||||

TS-165 18571 TACATGGCACTATCTTGTGGGTCCAATGCTAGTTGACTTTTTTTTCAAGTTAGTGCCACG 18630

SG-7 18601 TAGGACGAAAAGGGTAGAAAATTACTTCTAAAATAAGTTCAGGGGGAGAATAAGACCTTA 18660

||||||||||||||||||||||||||||||||||||||||||||||||||||||||||||

TS-165 18631 TAGGACGAAAAGGGTAGAAAATTACTTCTAAAATAAGTTCAGGGGGAGAATAAGACCTTA 18690

SG-7 18661 GTATAGTATAAGTGTGTCTCTGAAATTTCGAACACAGATTGAGGGGGTAATTGTGCATTT 18720

||||||||||||||||||||||||||||||||||||||||||| ||||||||||||||||

TS-165 18691 GTATAGTATAAGTGTGTCTCTGAAATTTCGAACACAGATTGAGAGGGTAATTGTGCATTT 18750

SG-7 18721 TCCTataattaataataagaataaattaaaaacaaagtaataaatttaattaTCACTAGA 18780

||||||||||||||||||||||||||||||||||||||||||||||||||||||||||||

TS-165 18751 TCCTATAATTAATAATAAGAATAAATTAAAAACAAAGTAATAAATTTAATTATCACTAGA 18810

SG-7 18781 TTTTTTGAACTGGATAAGTAAAAGACAAATATTTATTTTTGGAATATAAAAGATTGGGAG 18840

||||||||||||||||||||||||||||||||||||||||||||||||||||||||||||

TS-165 18811 TTTTTTGAACTGGATAAGTAAAAGACAAATATTTATTTTTGGAATATAAAAGATTGGGAG 18870

SG-7 18841 TAGTACATTCGTATTTTTGGAATGATAATGGTAAATAATAAAGATAAATTGAAAAGTAAA 18900

||||||||||||||||||||||||||||||||||||||||||||||||||||||||||||

TS-165 18871 TAGTACATTCGTATTTTTGGAATGATAATGGTAAATAATAAAGATAAATTGAAAAGTAAA 18930

SG-7 18901 TGATGattaaactatttcttatttttaattaaataataataaattataatatatcaacaa 18960

||||||||||||||||||||||||||||||||||||||||||||||||||||||||||||

TS-165 18931 TGATGATTAAACTATTTCTTATTTTTAATTAAATAATAATAAATTATAATATATCAACAA 18990

W1P9

SG-7 18961 ctatataaggattaaaaGGGAATCTGTTTCTGGTCTTTTTGTTTTGTTATGTTGTTGATA 19020

|||||||||||||||||||||||||| |||||||||||||||||||||||||||||||||

TS-165 18991 CTATATAAGGATTAAAAGGGAATCTGCTTCTGGTCTTTTTGTTTTGTTATGTTGTTGATA 19050

SG-7 19021 TTTGGTTGGTTGGATCATTGTTTACTAACTTATTGTTATTAAAATTATCTTCGAAAGGGA 19080

||||||||||||||||||||||||||||||||||||||||||||||||||||||||||||

TS-165 19051 TTTGGTTGGTTGGATCATTGTTTACTAACTTATTGTTATTAAAATTATCTTCGAAAGGGA 19110

SG-7 19081 TGATTATAATTGGTCCCATATAACTATATGATTGTTGCCTCCACAACAATAAGAAGTTAA 19140

||||||||||||||||||||||||||||||||||||||||||||||||||||||||||||

TS-165 19111 TGATTATAATTGGTCCCATATAACTATATGATTGTTGCCTCCACAACAATAAGAAGTTAA 19170

SG-7 19141 AATAATTACCTCTCTTTCTAATATAGTAACCCTCCCCACATAT 19183

|||||||||||||||||||||||||||||||||||||||||||

TS-165 19171 AATAATTACCTCTCTTTCTAATATAGTAACCCTCCCCACATAT 19213

**Partial mRNA sequence of *SlGID1a* gene in SG-7**

The coding sequence of *SlGID1a* gene was highlighted in purple color, the yellow highlighted 247th nucleotide of the coding sequence was C in SG-7

CTGAAGGAACAGTGTGTCGAGAGGGAGATGAAAGTACTACTGACAAAGAAAGAAGCTTTATTGTTGTTGGGGTTTTTCATGGAAATGTTCTTTCTTGGTTTTGTTTAATCGCTTGTGCTTTTTTTTTTTTTTTGCTTTTCACCAAATCAAGAAAAAGGAGAAGCGGCTGTCT**ATG**GCAAGAAATAATGAAGCTGTTGCGAATGAATCCAAGAGTGAATCTAAGAGAGTGGTTCCGCTCAATACATGGATCCTAATTTCAAACTTCAAGTTGTCTTACAATCTTCTCCGTCGCCCTGATGGGACTTTCAATCGTCACTTGGCAGAGTTCCTTGACCGCAAGGTTCCAGCGAATGCAAATCCAGTTGATGGAGTTTTCTCTTTTGATGTTCTCATTGATCGTGAAATAGGCCTACTTAGCCGTGTCTATCGGCCTTCTTTTGAGGATGGAGCTTCACCGAATATGGCTGAACTTGAAAAGCCTGTGACTGCTGATGTTGTACCTGTCATAATTTTCTTCCATGGTGGAAGTTTTGCACACTCTTCTTTCAATAGTGCCATCTATGACACACTTTGTCGCCGCCTTGTTGGCATTTGCAAGGCAGTTGTTGTGTCAGTTAATTACAGGCGAGCTCCTGAAAACCGTTATCCTTGTGCTTATAATGATGGATGGACAGTTCTTGAGTGGGTTAACTCAAGGGAATGGCTGCGGAGCAAAAAGGACTCGAAGGCTCACATATACTTAGCTGGAGATAGCTCTGGTGGTAATATTGTTCATAATGTGGCTTTCAGGGCAGTAGAATCCAACATAGAAGTGTTGGGAAATATACTGCTGAACCCTATGTTTGGTGGACAAGAGAGAACAGAATCAGAGAAGCGATTGGATGGCAAATATTTTGTCACACTTCAAGACCGAGACTGGTATTGGAGAGCTTATCTTCCTGAAGATTCAGACAGGGACCATCCTGCATGCAACCCTTTTGGTCCAAATGGTATAAACCTCAAAGGCGTCAAGTTCCCAAAGAATCTTGTTGTTGTCGCAGGTTTGGACCTTGTTCAGGATTGGCAGTTGGCTTATGCTGATGGGCTTAAGAAGGCTGGACAAGAGGTTAACCTGATATATTTGGAGAAGGCAACAATAGGGTTCTACCTGTTGCCAAATAATGAACACTTCTACACTGTCATGGATGAGATAAGTAGCTTCGTGAGTTCTGACTCTCAG**TAG**ATTTAACCTTGTCAAAAGTAGGATATGCTTTGAAGACGTTTGATGTTTTGTTGAAGTTAGTTCCTAGCCTGTCAACCGTTTGAAAGATTGTATAGCATCATCAATTACTTCCTTATTGATCATGCTATTGCTTGGATTCTGCTCCATTGGCTGGGTTGGTTATTGGTGGCGGAAAACCTCAATCATGTAGCTGGATCTGTGT

**Partial mRNA sequence of *SlGID1a* gene in TS-165**

The coding sequence of *SlGID1a* gene was highlighted in purple color, the yellow highlighted 247th nucleotide of the coding sequence was T in TS-165

CTGAAGGAACAGTGTGTCGAGAGGGAGATGAAAGTACTACTGACAAAGAAAGAAGCTTTATTGTTGTTGGGGTTTTTCATGGAAATGTTCTTTCTTGGTTTTGTTTAATCGCTTGTGCTTTTTTTTTTTTTTTGCTTTTCACCAAATCAAGAAAAAGGAGAAGCGGCTGTCT**ATG**GCAAGAAATAATGAAGCTGTTGCGAATGAATCCAAGAGTGAATCTAAGAGAGTGGTTCCGCTCAATACATGGATCCTAATTTCAAACTTCAAGTTGTCTTACAATCTTCTCCGTCGCCCTGATGGGACTTTCAATCGTCACTTGGCAGAGTTCCTTGACCGCAAGGTTCCAGCGAATGCAAATCCAGTTGATGGAGTTTTCTCTTTTGATGTTCTCATTGATCGTGAAATAGGCCTACTTAGCTGTGTCTATCGGCCTTCTTTTGAGGATGGAGCTTCACCGAATATGGCTGAACTTGAAAAGCCTGTGACTGCTGATGTTGTACCTGTCATAATTTTCTTCCATGGTGGAAGTTTTGCACACTCTTCTTTCAATAGTGCCATCTATGACACACTTTGTCGCCGCCTTGTTGGCATTTGCAAGGCAGTTGTTGTGTCAGTTAATTACAGGCGAGCTCCTGAAAACCGTTATCCTTGTGCTTATAATGATGGATGGACAGTTCTTGAGTGGGTTAACTCAAGGGAATGGCTGCGGAGCAAAAAGGACTCGAAGGCTCACATATACTTAGCTGGAGATAGCTCTGGTGGTAATATTGTTCATAATGTGGCTTTCAGGGCAGTAGAATCCAACATAGAAGTGTTGGGAAATATACTGCTGAACCCTATGTTTGGTGGACAAGAGAGAACAGAATCAGAGAAGCGATTGGATGGCAAATATTTTGTCACACTTCAAGACCGAGACTGGTATTGGAGAGCTTATCTTCCTGAAGATTCAGACAGGGACCATCCTGCATGCAACCCTTTTGGTCCAAATGGTATAAACCTCAAAGGCGTCAAGTTCCCAAAGAATCTTGTTGTTGTCGCAGGTTTGGACCTTGTTCAGGATTGGCAGTTGGCTTATGCTGATGGGCTTAAGAAGGCTGGACAAGAGGTTAACCTGATATATTTGGAGAAGGCAACAATAGGGTTCTACCTGTTGCCAAATAATGAACACTTCTACACTGTCATGGATGAGATAAGTAGCTTCGTGAGTTCTGACTCTCAG**TAG**ATTTAACCTTGTCAAAAGTAGGATATGCTTTGAAGACGTTTGATGTTTTGTTGAAGTTAGTTCCTAGCCTGTCAACCGTTTGAAAGATTGTATAGCATCATCAATTACTTCCTTATTGATCATGCTATTGCTTGGATTCTGCTCCATTGGCTGGGTTGGTTATTGGTGGCGGAAAACCTCAATCATGTAGCTGGATCTGTGT

**Alignment of the partial mRNA sequence of *SlGID1a* gene in SG-7 and TS-165**

The coding sequence and mutation of *SlGID1a* gene were highlighted in purple and yellow color respectively

SG-7 1 CTGAAGGAACAGTGTGTCGAGAGGGAGATGAAAGTACTACTGACAAAGAAAGAAGCTTTA 60

||||||||||||||||||||||||||||||||||||||||||||||||||||||||||||

TS-165 1 CTGAAGGAACAGTGTGTCGAGAGGGAGATGAAAGTACTACTGACAAAGAAAGAAGCTTTA 60

SG-7 61 TTGTTGTTGGGGTTTTTCATGGAAATGTTCTTTCTTGGTTTTGTTTAATCGCTTGTGCtt 120

||||||||||||||||||||||||||||||||||||||||||||||||||||||||||||

TS-165 61 TTGTTGTTGGGGTTTTTCATGGAAATGTTCTTTCTTGGTTTTGTTTAATCGCTTGTGCTT 120

SG-7 121 tttttttttttttGCTTTTCACCAAATCAAGAAAAAGGAGAAGCGGCTGTCT**ATG**GCAAG 180

||||||||||||||||||||||||||||||||||||||||||||||||||||**|||**|||||

TS-165 121 TTTTTTTTTTTTTGCTTTTCACCAAATCAAGAAAAAGGAGAAGCGGCTGTCT**ATG**GCAAG 180

SG-7 181 AAATAATGAAGCTGTTGCGAATGAATCCAAGAGTGAATCTAAGAGAGTGGTTCCGCTCAA 240

||||||||||||||||||||||||||||||||||||||||||||||||||||||||||||

TS-165 181 AAATAATGAAGCTGTTGCGAATGAATCCAAGAGTGAATCTAAGAGAGTGGTTCCGCTCAA 240

SG-7 241 TACATGGATCCTAATTTCAAACTTCAAGTTGTCTTACAATCTTCTCCGTCGCCCTGATGG 300

||||||||||||||||||||||||||||||||||||||||||||||||||||||||||||

TS-165 241 TACATGGATCCTAATTTCAAACTTCAAGTTGTCTTACAATCTTCTCCGTCGCCCTGATGG 300

SG-7 301 GACTTTCAATCGTCACTTGGCAGAGTTCCTTGACCGCAAGGTTCCAGCGAATGCAAATCC 360

||||||||||||||||||||||||||||||||||||||||||||||||||||||||||||

TS-165 301 GACTTTCAATCGTCACTTGGCAGAGTTCCTTGACCGCAAGGTTCCAGCGAATGCAAATCC 360

SG-7 361 AGTTGATGGAGTTTTCTCTTTTGATGTTCTCATTGATCGTGAAATAGGCCTACTTAGCCG 420

|||||||||||||||||||||||||||||||||||||||||||||||||||||||||| |

TS-165 361 AGTTGATGGAGTTTTCTCTTTTGATGTTCTCATTGATCGTGAAATAGGCCTACTTAGCTG 420

SG-7 421 TGTCTATCGGCCTTCTTTTGAGGATGGAGCTTCACCGAATATGGCTGAACTTGAAAAGCC 480

||||||||||||||||||||||||||||||||||||||||||||||||||||||||||||

TS-165 421 TGTCTATCGGCCTTCTTTTGAGGATGGAGCTTCACCGAATATGGCTGAACTTGAAAAGCC 480

SG-7 481 TGTGACTGCTGATGTTGTACCTGTCATAATTTTCTTCCATGGTGGAAGTTTTGCACACTC 540

||||||||||||||||||||||||||||||||||||||||||||||||||||||||||||

TS-165 481 TGTGACTGCTGATGTTGTACCTGTCATAATTTTCTTCCATGGTGGAAGTTTTGCACACTC 540

SG-7 541 TTCTTTCAATAGTGCCATCTATGACACACTTTGTCGCCGCCTTGTTGGCATTTGCAAGGC 600

||||||||||||||||||||||||||||||||||||||||||||||||||||||||||||

TS-165 541 TTCTTTCAATAGTGCCATCTATGACACACTTTGTCGCCGCCTTGTTGGCATTTGCAAGGC 600

SG-7 601 AGTTGTTGTGTCAGTTAATTACAGGCGAGCTCCTGAAAACCGTTATCCTTGTGCTTATAA 660

||||||||||||||||||||||||||||||||||||||||||||||||||||||||||||

TS-165 601 AGTTGTTGTGTCAGTTAATTACAGGCGAGCTCCTGAAAACCGTTATCCTTGTGCTTATAA 660

SG-7 661 TGATGGATGGACAGTTCTTGAGTGGGTTAACTCAAGGGAATGGCTGCGGAGCAAAAAGGA 720

||||||||||||||||||||||||||||||||||||||||||||||||||||||||||||

TS-165 661 TGATGGATGGACAGTTCTTGAGTGGGTTAACTCAAGGGAATGGCTGCGGAGCAAAAAGGA 720

SG-7 721 CTCGAAGGCTCACATATACTTAGCTGGAGATAGCTCTGGTGGTAATATTGTTCATAATGT 780

||||||||||||||||||||||||||||||||||||||||||||||||||||||||||||

TS-165 721 CTCGAAGGCTCACATATACTTAGCTGGAGATAGCTCTGGTGGTAATATTGTTCATAATGT 780

SG-7 781 GGCTTTCAGGGCAGTAGAATCCAACATAGAAGTGTTGGGAAATATACTGCTGAACCCTAT 840

||||||||||||||||||||||||||||||||||||||||||||||||||||||||||||

TS-165 781 GGCTTTCAGGGCAGTAGAATCCAACATAGAAGTGTTGGGAAATATACTGCTGAACCCTAT 840

SG-7 841 GTTTGGTGGACAAGAGAGAACAGAATCAGAGAAGCGATTGGATGGCAAATATTTTGTCAC 900

||||||||||||||||||||||||||||||||||||||||||||||||||||||||||||

TS-165 841 GTTTGGTGGACAAGAGAGAACAGAATCAGAGAAGCGATTGGATGGCAAATATTTTGTCAC 900

SG-7 901 ACTTCAAGACCGAGACTGGTATTGGAGAGCTTATCTTCCTGAAGATTCAGACAGGGACCA 960

||||||||||||||||||||||||||||||||||||||||||||||||||||||||||||

TS-165 901 ACTTCAAGACCGAGACTGGTATTGGAGAGCTTATCTTCCTGAAGATTCAGACAGGGACCA 960

SG-7 961 TCCTGCATGCAACCCTTTTGGTCCAAATGGTATAAACCTCAAAGGCGTCAAGTTCCCAAA 1020

||||||||||||||||||||||||||||||||||||||||||||||||||||||||||||

TS-165 961 TCCTGCATGCAACCCTTTTGGTCCAAATGGTATAAACCTCAAAGGCGTCAAGTTCCCAAA 1020

SG-7 1021 GAATCTTGTTGTTGTCGCAGGTTTGGACCTTGTTCAGGATTGGCAGTTGGCTTATGCTGA 1080

||||||||||||||||||||||||||||||||||||||||||||||||||||||||||||

TS-165 1021 GAATCTTGTTGTTGTCGCAGGTTTGGACCTTGTTCAGGATTGGCAGTTGGCTTATGCTGA 1080

SG-7 1081 TGGGCTTAAGAAGGCTGGACAAGAGGTTAACCTGATATATTTGGAGAAGGCAACAATAGG 1140

||||||||||||||||||||||||||||||||||||||||||||||||||||||||||||

TS-165 1081 TGGGCTTAAGAAGGCTGGACAAGAGGTTAACCTGATATATTTGGAGAAGGCAACAATAGG 1140

SG-7 1141 GTTCTACCTGTTGCCAAATAATGAACACTTCTACACTGTCATGGATGAGATAAGTAGCTT 1200

||||||||||||||||||||||||||||||||||||||||||||||||||||||||||||

TS-165 1141 GTTCTACCTGTTGCCAAATAATGAACACTTCTACACTGTCATGGATGAGATAAGTAGCTT 1200

SG-7 1201 CGTGAGTTCTGACTCTCAG**TAG**ATTTAACCTTGTCAAAAGTAGGATATGCTTTGAAGACG 1260

|||||||||||||||||||**|||**||||||||||||||||||||||||||||||||||||||

TS-165 1201 CGTGAGTTCTGACTCTCAG**TAG**ATTTAACCTTGTCAAAAGTAGGATATGCTTTGAAGACG 1260

SG-7 1261 TTTGATGTTTTGTTGAAGTTAGTTCCTAGCCTGTCAACCGTTTGAAAGATTGTATAGCAT 1320

||||||||||||||||||||||||||||||||||||||||||||||||||||||||||||

TS-165 1261 TTTGATGTTTTGTTGAAGTTAGTTCCTAGCCTGTCAACCGTTTGAAAGATTGTATAGCAT 1320

SG-7 1321 CATCAATTACTTCCTTATTGATCATGCTATTGCTTGGATTCTGCTCCATTGGCTGGGTTG 1380

||||||||||||||||||||||||||||||||||||||||||||||||||||||||||||

TS-165 1321 CATCAATTACTTCCTTATTGATCATGCTATTGCTTGGATTCTGCTCCATTGGCTGGGTTG 1380

SG-7 1381 GTTATTGGTGGCGGAAAACCTCAATCATGTAGCTGGATCTGTGT 1424

||||||||||||||||||||||||||||||||||||||||||||

TS-165 1381 GTTATTGGTGGCGGAAAACCTCAATCATGTAGCTGGATCTGTGT 1424

**Predicted protein sequence of SlGID1a in SG-7**

The yellow highlighted 83rd amino acid residue in the predicted protein sequence was Arginine (R) in SG-7

MARNNEAVANESKSESKRVVPLNTWILISNFKLSYNLLRRPDGTFNRHLAEFLDRKVPANANPVDGVFSFDVLIDREIGLLSRVYRPSFEDGASPNMAELEKPVTADVVPVIIFFHGGSFAHSSFNSAIYDTLCRRLVGICKAVVVSVNYRRAPENRYPCAYNDGWTVLEWVNSREWLRSKKDSKAHIYLAGDSSGGNIVHNVAFRAVESNIEVLGNILLNPMFGGQERTESEKRLDGKYFVTLQDRDWYWRAYLPEDSDRDHPACNPFGPNGINLKGVKFPKNLVVVAGLDLVQDWQLAYADGLKKAGQEVNLIYLEKATIGFYLLPNNEHFYTVMDEISSFVSSDSQ

**Predicted protein sequence of SlGID1a in TS-165**

The yellow highlighted 83rd amino acid residue in the predicted protein sequence was Cysteine (C) in TS-165

MARNNEAVANESKSESKRVVPLNTWILISNFKLSYNLLRRPDGTFNRHLAEFLDRKVPANANPVDGVFSFDVLIDREIGLLSCVYRPSFEDGASPNMAELEKPVTADVVPVIIFFHGGSFAHSSFNSAIYDTLCRRLVGICKAVVVSVNYRRAPENRYPCAYNDGWTVLEWVNSREWLRSKKDSKAHIYLAGDSSGGNIVHNVAFRAVESNIEVLGNILLNPMFGGQERTESEKRLDGKYFVTLQDRDWYWRAYLPEDSDRDHPACNPFGPNGINLKGVKFPKNLVVVAGLDLVQDWQLAYADGLKKAGQEVNLIYLEKATIGFYLLPNNEHFYTVMDEISSFVSSDSQ

SlGID1a MARNNEAVANESKSESKRVVPLNTWILISNFKLSYNLLRRPDGTFNRHLAEFLDRKVPANANPV 64
SlGID1a^R83C^ MARNNEAVANESKSESKRVVPLNTWILISNFKLSYNLLRRPDGTFNRHLAEFLDRKVPANANPV 64
AtGID1A MAASDEVNLIESRT----VVPLNTWVLISNFKVAYNILRRPDGTFNRHLAEYLDRKVTANANPV 60
AtGID1C MAGSEEVNLIESKT----VVPLNTWVLISNFKLAYNLLRRPDGTFNRHLAEFLDRKVPANANPV 60
SlGID1b1 MVDTKEINTNESKR----VVPLNTWILISNFKLAYNMLRRSDGTFNRDLAEFLERKVGANSIPV 60
SlGID1b2 MAGSNEINANESKR----VVPLNTWILISNFKLSYNMLRRPDGTFDRDLAEFIDRKVPTNSIPV 60
AtGID1B MAGGNEVNLNECKR----IVPLNTWVLISNFKLAYKVLRRPDGSFNRDLAEFLDRKVPANSFPL 60
OsGID1 MAGSDEVNRNECKT----VVPLHTWVLISNFKLSYNILRRADGTFERDLGEYLDRRVPANARPL 60

SlGID1a DGVFSFDVLIDREIGLLSRVYRPSFED------G-ASPNMAELEKPVTA-DVVPVIIFFHGGSF 120
SlGID1a^R83C^ DGVFSFDVLIDREIGLLSCVYRPSFED------G-ASPNMAELEKPVTA-DVVPVIIFFHGGSF 120
AtGID1A DGVFSFDVLIDRRINLLSRVYRPAYAD------QEQPPSILDLEKPVDG-DIVPVILFFHGGSF 117
AtGID1C NGVFSFDVIIDRQTNLLSRVYRPADAG--------TSPSITDLQNPVDG-EIVPVIVFFHGGSF 115
SlGID1b1 DGVYSFDV-VDRCTSLLNRVYKPAPK-------NECDWGKIDLDTPLSTSEIVPVIIFFHGGSF 116
SlGID1b2 DGVYSFDV-FDRVTSLLNRIYRPAPE-------NEADWGKIELEKPLSTTEIVPVIIYFHGGSF 116
AtGID1B DGVFSFDH-VDSTTNLLTRIYQPASLL------HQTRHGTLELTKPLSTTEIVPVLIFFHGGSF 117
OsGID1 EGVSSFDHIIDQSVGLEVRIYRAAAEGDAEEGAAAVTRPILEFLTDAPAAEPFPVIIFFHGGSF 124

SlGID1a AHSSFNSAIYDTLCRRLVGICKAVVVSVNYRRAPENRYPCAYNDGWTVLEWVNSREWLRSKKDS 184
SlGID1a^R83C^ AHSSFNSAIYDTLCRRLVGICKAVVVSVNYRRAPENRYPCAYNDGWTVLEWVNSREWLRSKKDS 184
AtGID1A AHSSANSAIYDTLCRRLVGLCKCVVVSVNYRRAPENPYPCAYDDGWIALNWVNSRSWLKSKKDS 181
AtGID1C AHSSANSAIYDTLCRRLVGLCGAVVVSVNYRRAPENRYPCAYDDGWAVLKWVNSSSWLRSKKDS 179
SlGID1b1 THSSANSAIYDTFCRRLVSICKAVVVSVNYRRSPENRYPCAYDDGWAALQWVKSRAWLQSGEDL 180
SlGID1b2 THSSANSAIYDTFCRRLVKICKAVVVSVNYRRSPEHRYPCAYDDGWAALKWVKSRTWLQSGKDS 180
AtGID1B THSSANSAIYDTFCRRLVTICGVVVVSVDYRRSPEHRYPCAYDDGWNALNWVKSRVWLQSGKDS 181
OsGID1 VHSSASSTIYDSLCRRFVKLSKGVVVSVNYRRAPEHRYPCAYDDGWTALKWVMSQPFMRSGGDA 188

SlGID1a KAHIYLAGDSSGGNIVHNVAFRAVESNIEVLGNILLNPMFGGQERTESEKRLDGKYFVTLQDRD 248
SlGID1a^R83C^ KAHIYLAGDSSGGNIVHNVAFRAVESNIEVLGNILLNPMFGGQERTESEKRLDGKYFVTLQDRD 248
AtGID1A KVHIFLAGDSSGGNIAHNVALRAGESGIDVLGNILLNPMFGGNERTESEKSLDGKYFVTVRDRD 245
AtGID1C KVRIFLAGDSSGGNIVHNVAVRAVESRIDVLGNILLNPMFGGTERTESEKRLDGKYFVTVRDRD 243
SlGID1b1 KVHVYMSGDSSGGNIAHHVAVQAAESGVEVLGNILLHPMFGGQNRTESESRLDGKYFVTVQDRD 244
SlGID1b2 KVHVYMAGDSSGGNIAHHVAVRAAEAGVEVLGNIHLHPMFGGQNRTESEKRLDGKYFVTVQDRD 244
AtGID1B NVYVYLAGDSSGGNIAHNVAVRATNEGVKVLGNILLHPMFGGQERTQSEKTLDGKYFVTIQDRD 245
OsGID1 QARVFLSGDSSGGNIAHHVAVRAADEGVKVCGNILLNAMFGGTERTESERRLDGKYFVTLQDRD 252

SlGID1a WYWRAYLPEDSDRDHPACNPFGPNGINLKGVKFPKNLVVVAGLDLVQDWQLAYADGLKKAGQEV 312
SlGID1a^R83C^ WYWRAYLPEDSDRDHPACNPFGPNGINLKGVKFPKNLVVVAGLDLVQDWQLAYADGLKKAGQEV 312
AtGID1A WYWKAFLPEGEDREHPACNPFSPRGKSLEGVSFPKSLVVVAGLDLIRDWQLAYAEGLKKAGQEV 309
AtGID1C WYWRAFLPEGEDREHPACSPFGPRSKSLEGLSFPKSLVVVAGLDLIQDWQLKYAEGLKKAGQEV 307
SlGID1b1 WYWRAYLPVGEDRDHPACNIFGPRGKTLQGLKFPKSLVVVAGLDLVQDWQLNYVEGLKKSGHEV 308
SlGID1b2 WYWRAYLPEGEDRDHPACNIFGSRSRSLKGLKFPKSLVVVAGLDLSQDWQLAYVDGLKNSGHEV 308
AtGID1B WYWRAYLPEGEDRDHPACNPFGPRGQSLKGVNFPKSLVVVAGLDLVQDWQLAYVDGLKKTGLEV 309
OsGID1 WYWKAYLPEDADRDHPACNPFGPNGRRLGGLPFAKSLIIVSGLDLTCDRQLAYADALREDGHHV 316

SlGID1a NLIYLEKATIGFYLLPNNEHFYTVMDEISSFVSSDSQ------------ 349
SlGID1a^R83C^ NLIYLEKATIGFYLLPNNEHFYTVMDEISSFVSSDSQ------------ 349
AtGID1A KLMHLEKATVGFYLLPNNNHFHNVMDEISAFVNAEC------------- 345
AtGID1C KLLYLEQATIGFYLLPNNNHFHTVMDEIAAFVNAECQ------------ 344
SlGID1b1 NLLYLKQATIGFYFLPNNDHFRCLMEEITSFIHPNHS------------ 345
SlGID1b2 NLLYLKQATIGFYFLPNNDHFPCLMEEITNFIHPNCS------------ 345
AtGID1B NLLYLKQATIGFYFLPNNDHFHCLMEELNKFVHSIEDSQSKSSPVLLTP 358
OsGID1 KVVQCENATVGFYLLPNTVHYHEVMEEISDFLNANLYY----------- 354

**Supplementary Figure 3.** Multiple sequence alignment of GID1s based on full-length protein. Entire protein sequences were aligned by using ClustalX2 (Larkin et al., 2007) with default settings, and the conserved amino acids were shaded by using GeneDoc (2.6) (Nicholas et al., 1997). The arrow points the amino acid alteration between SlGID1a and SlGID1a^R83C^. The sequences of SlGID1a, SlGID1b1, SlGID1b2 were downloaded from SGN (Solanaceae Genomics Network, https://solgenomics.net/) using the accession numbers Solyc01g098390, Solyc09g074270, Solyc06g008870, respectively. The sequences of AtGID1A, AtGID1C, AtGID1B were downloaded from TAIR (The Arabidopsis Information Resource, https://www.arabidopsis.org/) using the accession numbers AT3G05120.1, AT3G63010.1, AT5G27320.1, respectively. The sequence of OsGID1 was downloaded from NCBI (National Center for Biotechnology Information, https://www.ncbi.nlm.nih.gov/) using the accession number XP_015639961.

**Reference**

Larkin, M. A., Blackshields, G., Brown, N. P., Chenna, R., McGettigan, P. A., McWilliam, H., et al. (2007). Clustal W and Clustal X version 2.0. *Bioinformatics* 23, 2947-2948. doi: 10.1093/bioinformatics/btm404

Nicholas, K. B., Nicholas, H. B. J., Deerfield, and D. W. I. (1997). GeneDoc: analysis and visualization of genetic variation. *EMBNEW. NEWS* 4, 14.
